# Slowed aging during reproductive dormancy is reflected in genome-wide transcriptome changes in *Drosophila melanogaster*

**DOI:** 10.1186/s12864-016-2383-1

**Published:** 2016-01-13

**Authors:** Lucie Kučerová, Olga I. Kubrak, Jonas M. Bengtsson, Hynek Strnad, Sören Nylin, Ulrich Theopold, Dick R. Nässel

**Affiliations:** Department of Molecular Biosciences, Wenner-Gren Institute, Stockholm, Sweden; Department of Zoology, Stockholm University, S-106 91 Stockholm, Sweden; Institute of Molecular Genetics, Academy of Sciences of the Czech Republic, Prague, Czech Republic

## Abstract

**Background:**

In models extensively used in studies of aging and extended lifespan, such as *C. elegans* and *Drosophila,* adult senescence is regulated by gene networks that are likely to be similar to ones that underlie lifespan extension during dormancy. These include the evolutionarily conserved insulin/IGF, TOR and germ line-signaling pathways. Dormancy, also known as dauer stage in the larval worm or adult diapause in the fly, is triggered by adverse environmental conditions, and results in drastically extended lifespan with negligible senescence. It is furthermore characterized by increased stress resistance and somatic maintenance, developmental arrest and reallocated energy resources. In the fly *Drosophila melanogaster* adult reproductive diapause is additionally manifested in arrested ovary development, improved immune defense and altered metabolism. However, the molecular mechanisms behind this adaptive lifespan extension are not well understood.

**Results:**

A genome wide analysis of transcript changes in diapausing *D. melanogaster* revealed a differential regulation of more than 4600 genes. Gene ontology (GO) and KEGG pathway analysis reveal that many of these genes are part of signaling pathways that regulate metabolism, stress responses, detoxification, immunity, protein synthesis and processes during aging. More specifically, gene readouts and detailed mapping of the pathways indicate downregulation of insulin-IGF (IIS), target of rapamycin (TOR) and MAP kinase signaling, whereas Toll-dependent immune signaling, Jun-N-terminal kinase (JNK) and Janus kinase/signal transducer and activator of transcription (JAK/STAT) pathways are upregulated during diapause. Furthermore, we detected transcriptional regulation of a large number of genes specifically associated with aging and longevity.

**Conclusions:**

We find that many affected genes and signal pathways are shared between dormancy, aging and lifespan extension, including IIS, TOR, JAK/STAT and JNK. A substantial fraction of the genes affected by diapause have also been found to alter their expression in response to starvation and cold exposure in *D. melanogaster*, and the pathways overlap those reported in GO analysis of other invertebrates in dormancy or even hibernating mammals. Our study, thus, shows that *D. melanogaster* is a genetically tractable model for dormancy in other organisms and effects of dormancy on aging and lifespan.

**Electronic supplementary material:**

The online version of this article (doi:10.1186/s12864-016-2383-1) contains supplementary material, which is available to authorized users.

## Background

Aging and adult senescence in the fly *Drosophila* and the worm *Caenorhabditis elegans* are regulated by gene networks that may overlap with ones that underlie lifespan extension during dormancy in these organisms [1–7]. Among the regulatory networks likely to be shared are the evolutionarily conserved insulin/IGF, TOR and germ line-signaling pathways [1–3, 8]. This hypothesis is supported by the phenotype characteristic of dormancy, which includes developmental arrest, increased stress resistance and somatic maintenance, reallocated energy resources, accompanied by a drastically extended lifespan with negligible senescence [8–10]. Dormancy, in insects also known as diapause, is an adaptive shift in life history that can be triggered by particularly adverse environmental challenges [8, 11–15].

Diapause appears to be part of a spectrum of general and partially conserved stress syndromes in animals, with a physiological shift from a reproduction mode to extended survival [9, 14, 16]. In insects, this dormancy, with its accompanying suite of co-adapted traits, is preprogrammed and can occur at different stages of the life cycle, but almost always in a single specific stage in each species [12]. The fruitfly *Drosophila melanogaster* can enter a reproductive diapause in the adult stage that is facultative, shallow, and rapidly reversible [9, 17–20]. In the laboratory diapause is induced by exposing female flies to low temperature and short photoperiod soon after the adults have eclosed, and is characterized by attenuated production of vitellogenic eggs, increased nutrient stores and diminished senescence [9, 10, 20]. Since the molecular mechanisms behind dormancy and its links to slowed aging are not well understood in any organism, we embarked on an analysis of genes involved in diapause of the genetic model insect *D. melanogaster*.

In a recent paper we reported in detail the dynamic phenotypic changes associated with reproductive diapause in *D. melanogaster* [20]. Diapausing flies display reduced food intake, increased stores of carbohydrates and lipids, activated immune genes, altered expression of genes related to insulin- and glucagon-like signaling, display low mortality and overall a drastically extended lifespan [20]. It was furthermore shown that flies with loss of function mutations in insulin-like peptide genes *dilp2-3* and *dilp5* are more prone to enter diapause than wild type flies [20]. This is in agreement with several reports on the role of insulin signaling in insect diapause and other forms of stress responses [9, 21–27]. Insulin/IGF signaling (IIS) is also known to be critical in regulation of fecundity, metabolism, stress resistance and longevity [28–32], all of which are affected by the quiescence during diapause [8, 10, 27]. Thus, to further investigate the role of IIS and other signaling pathways in slowed aging during diapause, we performed a genome-wide transcriptome analysis of flies kept for three weeks in diapause. The resultant data set indicates a broad effect of diapause on gene regulation, with over 4500 genes showing at least twofold up- or downregulation, compared to flies kept at normal conditions. Our analysis reveals that a large fraction of the genes associated with aging and longevity in *Drosophila* [2, 3, 5, 33] are affected by diapause. Furthermore, we show that several relevant signaling pathways associated with increased stress tolerance and extended lifespan were differentially regulated at the transcript level. These include the IIS and target of rapamycin (TOR) pathways, as well as Toll, JAK/STAT, JNK and MAP kinase signaling, and there are also effects on peptide hormone, juvenile hormone and ecdysone signaling components. Importantly, we identified a large number of altered genes that are in common with other genome-wide searches for adaptive changes in life history traits in *D. melanogaster*, for instance cold resistance and starvation responses, indicating the modular usage of stress signaling pathways. Our study clearly shows that *D. melanogaster* is a powerful organism for modeling genetic regulation of dormancy and slowed aging across a range of species.

## Results and discussion

### Experimental design

In a previous study of *D. melanogaster* we showed that vitellogenesis is at its most reduced level after 3 weeks in diapause-inducing conditions at 11 °C and short photoperiod (10 L:14D) [20]. These conditions were also established as optimal for reproductive diapause by Saunders et al. [18], and we, thus, did not test the effects of low temperature only. More than 90 % of the virgin female flies (*Canton S* strain) exhibit previtellogenic ovaries after this period of induction. This state of ovary maturation is commonly used as a marker of reproductive diapause [9, 18]. Additional physiological traits also display a strong phenotype after three weeks [20]. Hence, in our present genome-wide transcription study we compared flies (Canton S) kept for 3 weeks in diapause conditions (3wD) with flies kept in non-diapause conditions (25 °C, 12 L:12D). Since senescence appears reduced during diapause [9, 20], we chose two time points for control flies to determine how aging of flies at 25 °C would influence gene expression. Thus, we collected sibling flies that were three weeks (3wN) and one week old (1wN) as controls.

### Bioinformatics analysis

Samples of virgin female flies were collected in four biological replicates and purified RNA was hybridized to Affymetrix GeneChip *Drosophila* Genome 2.0 arrays. A heat map of the 100 most variable probes (Additional file 1: Figure S1) indicates that the majority of the differences detected in our microarrays are due to transcriptome changes in diapause samples, whereas the age of our control flies (1wN or 3wN) only has a minor effect. Principle Component Analysis, (PCA; Additional file 2: Figure S2), revealed distinct transcriptional profiles for diapause samples versus controls, but failed to distinguish between 1wN and 3wN transcriptional profiles. Thus, for further bioinformatics analysis we focused on comparing our diapause samples primarily with 1wN control flies. The transcriptome data for all three comparisons can be found in the Additional file 3: Dataset S1.

**Fig. 1:**
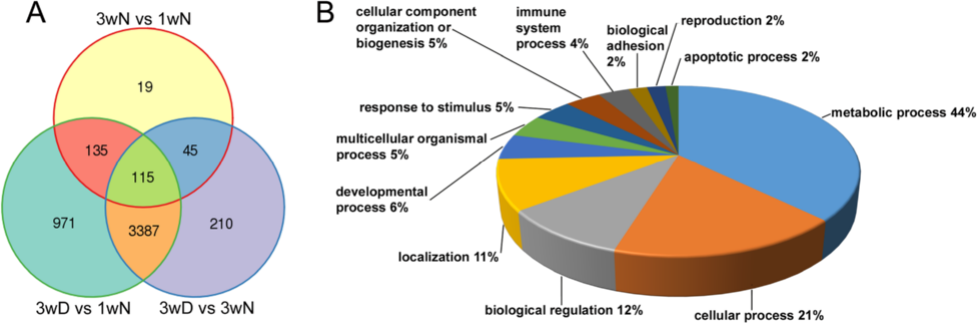
Diagrams summarizing differentially expressed transcripts under diapause and control conditions, and their functional classification based on gene ontology (GO) terms. **a** Out of 4624 differentially expressed transcripts, 3387 are detected in flies diapausing for 3 weeks (3wD) compared to both 1 (1wN) and 3 (3wN) week controls. Very few transcripts (19) differ due to the age difference between control flies. The transcripts shown display at least an absolute two-fold change and q < 0.05. **b** The functional classification of GO terms reveals that the majority of the affected genes are involved in metabolic (44 %) and cellular processes (21 %)

We identified 4624 differentially expressed transcripts (absolute fold change ≥ 2, q < 0.05), of which 2412 were upregulated and 2212 were downregulated in diapausing females. The Venn diagram in Fig. 1a shows that almost 80 % of these was also differentially regulated in comparison with the 3wN control. As verified by functional classification based on Gene Ontology (GO) terms (Fig. 1b) the diapause-regulated genes are mostly involved in metabolic processes (44 %) and cellular processes (21 %), which includes genes mainly associated with cell communication and the cell cycle. Significant changes were also observed in sets of genes regulating developmental processes (6 %), such as cell death and morphogenesis, as well as neurological system processes (5 %), and response to stimulus (5 %), where stimuli can be stress, abiotic or other external stimuli, pheromones, immune or toxic agents. About 4 % of the genes represent immune genes, and only a small subset of the genes (2 %) is involved in reproduction and gamete generation.

**Fig. 2:**
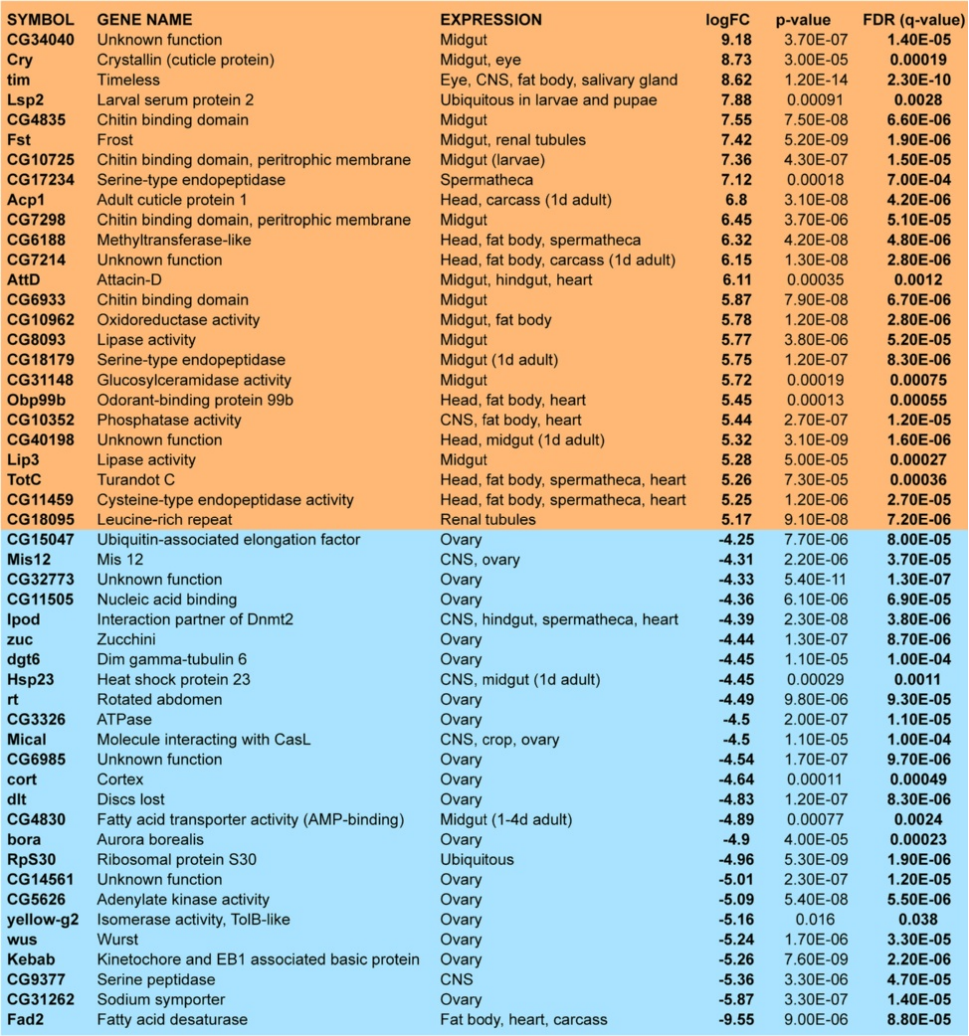
The 25 most up- and downregulated transcripts in diapausing flies. Flies diapausing for three weeks (3wD) were compared to one week old flies (1wN) kept at normal conditions. Upregulated transcripts are predominantly associated with the intestinal structures, fat body, spermatheca and heart, while the most downregulated ones are expressed in the ovaries intestinal structures and fat body (only Fad2). The changes are given as logarithmic fold change (logFC). For a complete list of altered gene expression see Additional file 3: Dataset S1

Among the top 25 most upregulated genes there is an enrichment of genes predominantly expressed in midgut and fat body (Fig. 2). In contrast the majority of the 25 most downregulated genes display enriched expression in the ovaries (Fig. 2). This apparent tissue distribution of the most strongly altered genes is in line with morphogenetic changes occurring in diapausing flies, as described earlier [20].

Gene set enrichment analysis (GSEA) of KEGG (Kyoto Encyclopedia of Genes and Genomes) pathways (Additional file 4: Table S1) confirms the functional GO classification analysis and adds additional information. The altered transcripts belong to 126 different KEGG pathways. From those GSEA identified 48 to be significantly enriched in our study. Of these, 30 KEGG pathways are mostly enriched for upregulated genes and 18 for downregulated genes (Additional file 4: Table S1). As in the GO classification, we detected strong enrichment of upregulated genes associated with different metabolic pathways (mostly basic energetic and storage metabolism), but also drug metabolism and detoxification with cytochrome P450. We also found enhanced Extracellular Matrix (ECM) receptor interactions, many genes involved in the lysosome pathway, and enrichment of genes involved in circadian rhythm. GSEA identified a number of significantly downregulated genes involved in DNA replication, homologous recombination, nucleotide excision repair, transcription, translation and protein processing (Additional file 4: Table S1). This is likely due to general downregulation or silencing of cell division and protein synthesis during dormancy and low cellular activity at low temperature.

However, from GSEA alone we cannot determine how expression of specific genes influences signaling in a particular pathway, since the genes can represent both inhibitors and positive regulators. To circumvent this we implemented Signal pathway impact analysis (SPIA) [34]. We used the KEGG pathway database for search with SPIA and found 4 pathways that are significantly influenced (Additional file 5: Table S2); all of them were previously identified in the GSEA. The SPIA confirmed that ECM receptor interactions are activated during diapause. Furthermore suppression of the circadian rhythm pathway in diapause was seen. In this context we curiosly found an increased expression of the clock genes *timeless* (*tim*), *period* (*per*), *shaggy* (*sgg*), and *vrille* (*vri*), which are among the top 100 most strongly induced genes in our study, and slightly (twofold) down-regulated *cycle* (*Cyc*) (Additional file 3: Dataset S1). It should be noted that clock genes are also expressed outside of the bona fide clock neurons of the brain, and since our analysis is performed on whole animals we cannot exclude that transcript changes are in other tissues. The KEGG pathways with ID numbers 3460 and 4914 are defined only on the basis of sequence similarity of *Drosophila* genes with those of humans and other mammals, and therefore their relevance in *Drosophila* is uncertain. However, since KEGG number 4914 (*Progesterone-mediated oocyte maturation*) partially overlaps with insulin and MAPK signaling pathways and was identified by SPIA to be significantly downregulated, we decided to analyze selected pathways closer by manual annotation.

### Confirmation of altered gene expression by qPCR

**Fig. 3:**
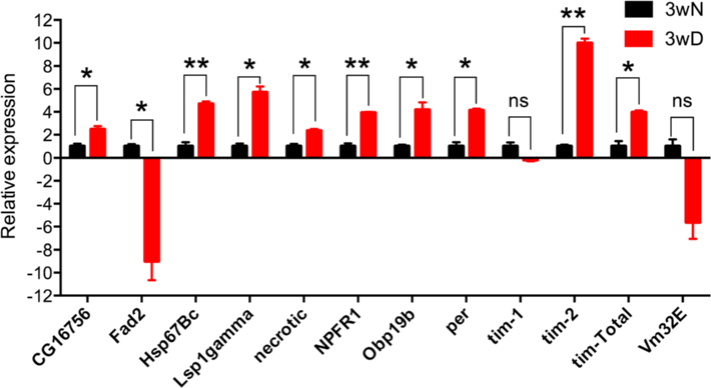
Quantitative real-time PCR confirms transcript changes in diapausing flies. Estimate of relative gene expression, comparing (ΔΔCt) three week old diapausing flies (3wD) with three week old flies kept under normal conditions (3wN). Directionality and relative magnitude of change matches microarray data in all cases, except Vm32E. We used Welch’s unpaired *t*-test with corrections for non-equal variances, comparing expression at 3wD and 3wN for each gene, ns, not significant; *, *p* < 0.05; **, *p* < 0.01. Error bars show standard error of the mean, *n* = 4. Acronyms used: *FAD2* - *Fatty Acid Desaturase 2, HS67Bc - Heat Shock 67 Bc, LSPgamma - Larval Serum Protein gamma, NeurpepRecF - Neuropeptide receptor F, OBP19b - Odorant Binding Protein 19 b, Per – Period, Tim-1 - Timeless,* (targets all Timeless isoforms except N and O)*, Tim-2 - Timeless*, (targets N and O isoforms of *Timeless*), *Tim-Total* - *Timeless*, (targets all *Timeless* isoforms), *VME32e* - Vitelline Membrane 32e

**Fig. 4:**
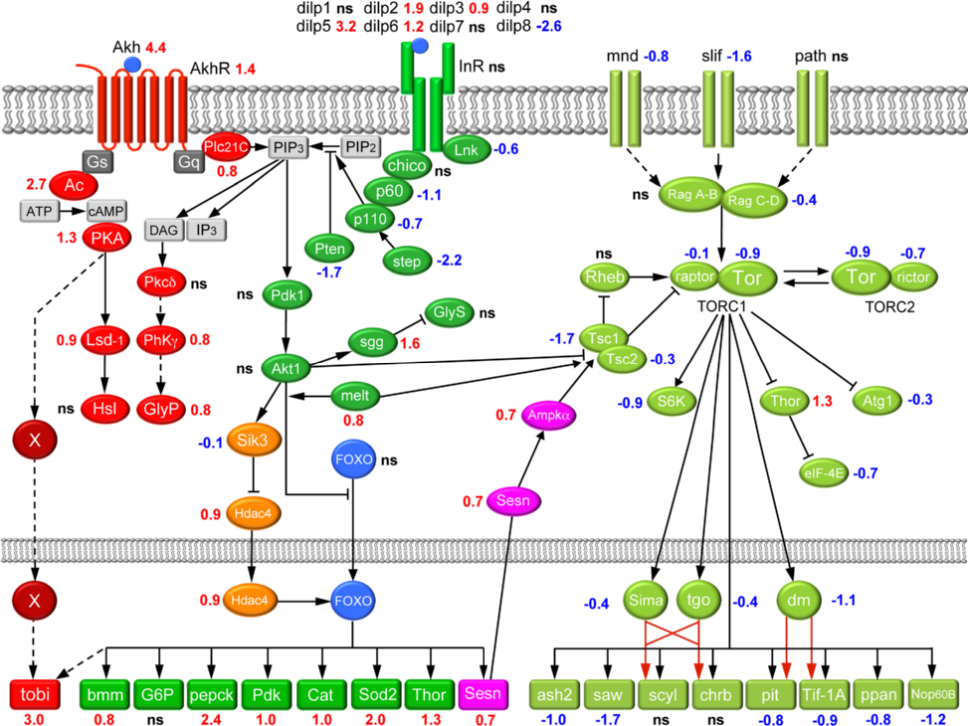
Altered gene expression in AKH, insulin/IGF- and TOR-signaling pathways in diapausing flies. The scheme displays a generalized assembly of relevant genes (regardless tissue-specificity) in the three signal pathways. Transcript levels (logarithmic fold change, LogFC) are given in red for upregulated, blue for downregulated and black for no significant change (ns; LogFC close to 0). The acronyms are listed in Additional file 6: Table S3 where also references and details of gene/protein functions are given. The AKH signaling is generally upregulated and the expression of the target gene *tobi* increased (X depicts an unidentified transcription factor). The Insulin/IGF signaling is generally downregulated and as a result several of the FOXO-regulated target genes are upregulated. The TOR-signaling is also downregulated, together with a number of its target genes. The AKH signaling scheme was compiled after [68, 171], the insulin/IGF signaling based on elements from [29, 36] and TOR signaling assembled after [35, 57]

We chose 12 genes from the microarray for confirmation by qPCR. Analysis of RNA extracted from flies kept at 3wN and 3wD showed that the qPCR data for 11 of these are in agreement with the changes of transcript levels seen in the microarray (Fig. 3). One gene, the *Vm32E* (*Vitelline membrane 32E*) gene, exhibits a large decrease in the microarray, but low statistical significance. With qPCR we confirmed the variability in expression of *Vm32E* in 3wD samples, which explains the incongruence of statistical results (Fig. 3). The variability in expression of this gene may reflect that most, but not all, of the *Canton S* females suppress vitellogenesis in diapause; we always observed a small percentage of escapers [20]. Two of the genes tested (*fatty acid desaturase, Fad2*, and *timeless*, *tim*) are among the top three differentially regulated transcripts in the microarray, and this is confirmed by qPCR. For *tim*, microarray probes differentiate between subsets of the 11 known isoforms of the corresponding mRNA. Microarray data indicated that either one or both of the isoforms N and O were strongly upregulated during diapause, while other isoforms were not. Data from qPCR corroborates this pattern, with strong upregulation of N and/or O, and little change for other isoforms. Some gene transcripts had been assayed earlier from 1wN and 3wD flies [20] and the data are in agreement with the present microarray results. These (see Fig. 4) are genes encoding peptide hormones (*dilps2, 3, 5, 6*, and *Akh),* and metabolic read-out genes (*4ebp, tobi, bmm* and *pepck*).

### Insulin/IGF, TOR and AKH signaling is altered during diapause

*Drosophila* responds to environmental challenges and maintains metabolic and energy homeostasis by evolutionarily conserved signaling pathways, such as the insulin/IGF signaling (IIS) and target of rapamycin (TOR) pathways [31, 35–38]. These signaling pathways display crosstalk in regulation of metabolism, homeostasis, growth, development and longevity [32, 35, 36]. Previous studies have indicated that the IIS pathway may be central in diapause regulation in flies and mosquitos [9, 21, 22, 24, 39], whereas, to our knowledge, there is no experimental data on TOR signaling (reviewed in [8, 23]).

To identify whether a pathway is generally up- or downregulated in diapausing flies we assembled the transcript data into their presumed functional context in signaling pathways. We particularly focused on readouts from specific transcriptional targets that are important in metabolism, stress responses, development and growth. Fig. 4 shows an assembly of transcript changes in the IIS, adipokinetic hormone (AKH) and TOR pathways based on the comparison of 3wD and 1wN flies. Note that due to the large number of genes discussed for these pathways and the ones in the following sections we have assembled a set of tables (Additional file 6: Table S3) where details are given for each gene, including relevant literature references.

#### Insulin/IGF signaling is downregulated

Based on transcriptional changes in several known IIS and FOXO target genes and the downregulation of several core IIS pathway components, our analysis suggests that IIS generally is downregulated during diapause (Fig. 4).

In diapausing flies we noted an up-regulation of *dilp2, 3, 5 and 6,* similar to a previous study [20], but a strong down-regulation of *dilp8,* a known coordinator of growth and development [40, 41]. We failed to observe any change in expression of the insulin receptor, *InR,* or the receptor substrate *chico*. However, *Lnk* (SH2B family of adaptor molecules; [42]), as well as the catalytic (*Dp110*) and adaptor (*p60*) subunits of PI3K, together with *Steppke* (*step*), an upstream regulator of PI3K [43] were down-regulated. *Akt1* and *Pdk1 (phosphatidylinositol-dependent kinase 1*) expression was unaffected by diapause. Taken together, these data suggest attenuated IIS signaling during diapause, leading to reduced FOXO phosphorylation and thus increased FOXO translocation into the nucleus. This is supported by an increase in transcription of HDAC4, which may activate FOXO [44] in diapausing flies via increased deacetylation. The downregulation of IIS is further supported by the decreased expression of salt-inducible kinase 3 (*Sik3*), responsible for the phosphorylation and inactivation of HDAC4 [44]. Increased translocation of unphosphorylated HDAC4 into the nucleus could result in FOXO deacetylation and increased DNA-binding activity [44, 45]. The increased FOXO action is likely to increase activation of transcription of catabolic enzymes and other FOXO targets, including *phosphoenolpyruvate carboxykinase (pepck)* and *brummer* (*bmm*) TAG lipase [44], as observed in diapausing *D. melanogaster*. The enhanced transcription of *bmm*, the fly homolog of adipose triglyceride lipase (ATGL) [46], indicates an activation of lipolysis. Increased *pepck* transcription points towards an enhancement of both glyconeogenesis and glyceroneogenesis [47] and was previously recorded in diapausing larvae of the mosquito *Wyeomyia smithii* [48]. Furthermore, our data indicate an upregulation of *pyruvate dehydrogenase kinase* (*Pdk*), responsible for a re-direction of pyruvate into anaerobic oxidation [49]. Activation of gluconeogenesis and other anaerobic metabolic pathways under diapause is a universal mechanism found in different insects [8, 10].

Among other transcriptional targets of FOXO, we detected increased expression of the antioxidant enzymes, catalase (*Cat*) and manganese superoxide dismutase (*Sod2*) in diapausing flies. This supports an enhanced stress resistance during diapause, consistent with earlier findings in *Drosophila* [9, 15]. Moreover, diapause triggered an increase in *Thor* transcript, encoding the eukaryotic initiation factor 4 binding protein (4EBP), an inhibitor of translation [50] that might be responsible for the diminished protein synthesis in diapausing flies. Finally, we observed an activation of *shaggy* (*sgg*), a GSK homolog. GSK3 is responsible for glycogen synthase (*GlyS*) inhibition [51], suggesting diminished glycogen synthesis in diapausing flies. In summary, our data suggest that the IIS pathway is significantly downregulated during diapause and that altered gene transcription may lead to increases in lipolysis, glycogenolysis, gluconeogenesis, glyceroneogenesis and stress resistance, and results in lowered aerobic pyruvate oxidation and glycogen synthesis (see Additional file 7: Figure S3). Importantly, downregulated IIS is known to extend lifespan in *Drosophila* and *C. elegans* [32, 52–54].

#### TOR signaling is downregulated

The transcript changes in diapausing flies reveal effects on TOR pathway components responsible for ribosome and protein biosynthesis, including AMPK-mediated growth suppression, as well as hypoxia-responsive transcription, and suggest a general downregulation of TOR signaling. Nutrient dependent TOR signaling is involved in the regulation of growth and metabolism and interacts with IIS, also in aging [32, 33, 36, 55, 56].

The diapause-induced downregulation of the rapamycin-sensitive TOR partners *raptor* and *Tor* in the TORC1 complex could indicate diminished translation and ribosome biosynthesis, possibly via phosphorylation of downstream targets such as S6 kinase (*S6K*) and 4E-BP (*Thor*) [57]. Downregulation of Rag GTPases (*RagC-D*, but not *RagA-B*) excludes stimulation of the TORC1 complex in diapausing flies. Concomitantly, but independently, the TORC1 complex is negatively regulated by the tuberous sclerosis complex (TSC), which inactivate the GTPase Rheb [58]. In diapausing flies the expression of the TORC1 suppressors *Tsc1* and *Tsc2* was decreased, whereas the TORC1 activators, *RagA-B, RagC-D* and *Rheb* were either not changed or decreased as well (Fig. 4).

The TORC1 decrease noted during diapause may be mediated by another mechanism involving AMPK (AMP-activated protein kinase), which can phosphorylate TSC2, and lead to TORC1 inhibition [59]. A further inhibitory effect of AMPK on TORC1 through phosphorylation of Raptor [60] cannot be excluded, while there is an energy deficit during diapause that requires AMPK-mediated suppression of growth and biosynthetic processes [8]. Moreover, the inhibitory effect of AMPK on TOR signaling during diapause could be enhanced by elevated *sestrin* (*Sesn*) expression, since *Sesn* transcription in *Drosophila* is under FOXO control and leads to AMPK activation and in turn an enhancement of Tsc1/Tsc2 inhibition of TORC1 [61]. The latter mechanism may be general in diapause regulation and has been suggested earlier for flesh flies and apple maggot flies [39, 62].

We find that decreased TOR signaling in diapausing flies is accompanied by a downregulation of S6 kinase (S6K), which together with downregulated Pol I transcription factor *Tif-IA* and transcription factor *diminutive* (*dm*), a *Drosophila* Myc homolog, may explain the decreased ribosome biogenesis (see above and [57, 58]). The enhanced *Thor* expression in our study is accompanied by a decreased level of eIF-4E transcript, indicating a suppression of transcription [50] during diapause. An enhanced transcription of the *Thor* gene, via FOXO, and lack of inhibitory phosphorylation of 4EBP protein from suppressed TORC1, was previously found to provide an adaptive decrease in energy-consuming translation during nutrient restriction with decreased IIS and TOR signaling [36, 50]. The diminished biosynthetic processes in diapausing flies was accompanied by a lower expression of a number of TOR readout genes, the growth regulators (*saw* and *ash2*), as well as *pitchoune* (*pit*), *peter pan* (*ppan*) and *Nop60B*/*minifly* (*Nop60B/mnf*), indicating arrested differentiation and development together with lowered fertility [63]. In addition we observed downregulation of the main hypoxia-inducible transcription factors Similar (*Sima*) and Tango (*tgo/Arnt*). These findings suggest reduced hypoxia-responsive transcription during diapause. In summary, our analysis indicates a general downregulation of the TOR signaling cascade during diapause. Furthermore, activators of TOR signaling, including nutrient sensing through amino acid transporters, like *slimfast* (*slif*) [64], or Akt-mediated stimulation of the TORC1 complex [65], are repressed in diapausing flies.

#### AKH signaling is upregulated

Our data suggest that AKH signaling may be upregulated during diapause. In *Drosophila* and other insects AKH signaling mobilizes stored energy reserves [66–70]. AKH signaling furthermore plays a role under stressful conditions, to potentiate rapid production of energy for survival of the organism [68]. A recent study indicates that upregulated AKH signaling extends lifespan, although the mechanisms are yet to be determined [71]. We find upregulated expression of both neuropeptide (*Akh*) and AKH receptor (*AkhR/GRHR*) transcripts, and several components of the two possible second messenger cascades downstream the receptor (Fig. 4, Table 1). These pathways act via phospholipase C (PLC; *Plc-21C*) or adenylyl cyclase (*Ac*) [68, 70, 72, 73]. The activation of PLC via the AKH receptor may lead to stimulation of glycogenolysis during diapause (see [69]). Elevation of cAMP, via *Ac,* and activation of protein kinase A (*Pka*), may suggest enhanced lipolysis in diapausing flies (see [74]). Furthermore, diapausing flies display elevated expression of *target of brain insulin* (*tobi*), which was postulated to be under control of AKH signaling through an unknown transcription factor [75]. Thus, it seems that in diapausing flies AKH signaling enhances glycogenolysis, either via activation of glycogen phosphorylase (*GlyP*) or stimulation of *tobi* expression, or both pathways.

**Table 1:** Neuropeptides, peptide hormones and receptors affected by diapause

Neuropeptide and/or GPCR^a^	Expression^b^	CG number	Log FC
Adipokinetic hormone (AKH)	Corpora cardiaca	CG1171	4.4
AKH receptor (AKHR)	Fat body, spermatheca	CG11325	1.4
Allatostatin A (Ast-A)	CNS, midgut	CG13633	1.9
Allatostatin B (Ast-B or MIP^c^)	CNS	CG6456	1.1
Allatostatin C (Ast-C)	CNS, midgut	CG14919	1.7
CAPA receptor (capaR)	Renal tubules	CG14575	1.3
CCHamide 2	Midgut, fat body	CG14375	2.1
Corazonin (CRZ)	CNS	CG3302	1.3
CRZ receptor (CrzR)	Fat body, heart	CG10698	2.0
DH_31_ receptor 1 (DH_31_–R1)	CNS, gut, renal tubules	CG32843	2.3
Diuretic hormone 44 (DH_44_)	CNS	CG8348	3.4
DH_44_ receptor 2 (DH_44_–R2)	CNS, gut, renal tubules	CG12370	2.5
Insulin-like peptide 2 (DILP2)	Brain	CG8167	1.9
DILP5	Brain, ovary	CG33273	3.2
DILP6	Fat body	CG14049	1.2
DILP8	Ovary	CG14059	−2.6
DLGR1^d^	Hindgut, salivary gland	CG7665	3.1
Dromyosuppressin (DMS)	Brain, heart	CG6440	1.5
Drosulfakinin (DSK)	Brain	CG18090	1.4
Ion transport peptide (ITP)	CNS, PNS	CG13586	1.2
ITG (*Apis*-ITG-like)	CNS	CG8216	3.0
Leucokinin (LK)	CNS	CG13480	2.1
LK receptor (LK-R)	CNS, renal tubules	CG10626	1.3
Neuropeptide F receptor (NPFR)	CNS, renal tubules	CG1147	2.4
NPLP1^e^	CNS	CG3441	2.4
NPLP3	Head, carcass	CG13061	2.1
NPLP4	Fat body (larva), Eye	CG15361	1.3
Orcokinin	CNS, gut	CG13565	1.6
Proctolin	CNS	CG7105	2.2
PTTH^f^	CNS, Renal tubules?	CG13687	1.1
Short neuropeptide F (sNPF)	CNS	CG13968	1.5
Tachykinin (DTK)	CNS, midgut	CG14734	2.5
Torso^g^	Ovary	CG1389	−3.3

*Notes*: The transcripts are sorted alphabetically (significantly >2 fold up- or downregulated; LogFC >1)

^a^Acronyms used are those for the proteins/peptides

^b^Based on FlyAtlas and/or modENCODE [116, 117], as well as research papers (summarized in [115]). Expression data are for adult flies, except NPLP4, where expression is most prominent in larvae

^c^MIP, myoinhibitory peptide

^d^Leucine-rich repeat-containing G protein-coupled receptor 1. Ligand is the dimeric GPA2/GPB5 protein

^e^Neuropeptide-like precursor 1

^f^Prothoracicotropic hormone

^g^Receptor tyrosine kinase, can be activated by PTTH [132]

### JAK/STAT signaling is affected in a tissue-specific manner

**Fig. 5:**
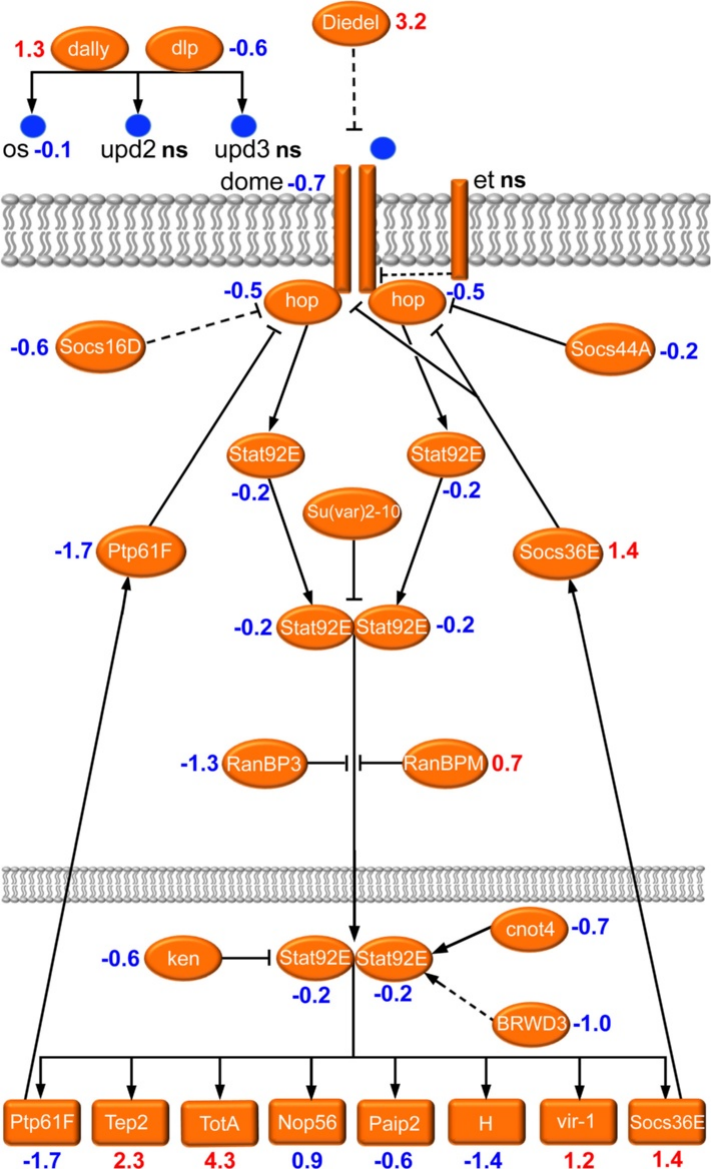
Altered gene expression in the JAK/STAT signaling pathway during diapause. This scheme displays a generalized assembly (regardless tissue specificity) of relevant genes in the JAK/STAT signal pathway. Transcript levels (logarithmic fold change, LogFC) are given in red for upregulated, blue for downregulated and black for no significant change (ns; LogFC close to 0). The acronyms are listed in Additional file 6: Table S3 where also references and details of gene/protein functions are given. At the whole organism level we observed a decreased expression of the core components of the JAK/STAT signaling and some read-out genes in diapausing flies. Some exceptions are seen in certain genes, involved in humoral immune response and stress resistance, which were upregulated (probably tissue-specific). The scheme is based partly on [172] and [80]. Further details are given in Additional file 6: Table S3 and text

Janus kinase/signal transducer and activator of transcription (JAK/STAT) signaling coordinates important biological processes in *Drosophila* during development and in adult physiology, such as cell proliferation and growth [76], organ formation [77], stem cell maintenance [78, 79] and immune response [80, 81] important during aging. At the whole organism level we observed a decreased expression of the core components of the JAK/STAT signaling and some read-out genes in diapausing flies (Fig. 5). The downregulated genes in the signaling pathway include *domeless* (*dome*) encoding a cytokine-like receptor, and *hopscotch* (hop) encoding *Drosophila* Janus kinase (JAK), as well as a single *Drosophila* STAT gene *Stat92E* [76, 81, 82]. Further downregulated genes are shown in Fig. 5 and details on gene function are given in Additional file 6: Table S3. Notable exceptions are the upregulated Stat92E targets *Turandot A* (*TotA*), known to play important roles in stress tolerance and immune response [83, 84], *vir-1* (*virus-induced RNA-1*), required for antiviral protection [85] and *Tep2* (Thioester-containing protein 2) that is part of the humoral immune response [86, 87]. These findings suggest that diapause triggers a tissue-specific upregulation of JAK/STAT signaling.

Other genes used as readout of the JAK/STAT pathway, including *dome* [88] and *Ptp61F* [89] displayed decreased expressions, whereas *Socs36E* [90] and *Turandot A* (*TotA*) [83] expression was increased. Most JAK/STAT target genes with Stat92E binding sites [91], including growth regulator *Nop56*, *Juvenile hormone epoxide hydrolase* (*Jheh2),* zinc finger transcription factor *ftz-f1,* negative regulator of Notch pathway *Hairless* (*H)* and negative regulator of translation *polyA-binding protein interacting protein 2 (Paip2)* were downregulated.

**Fig. 6:**
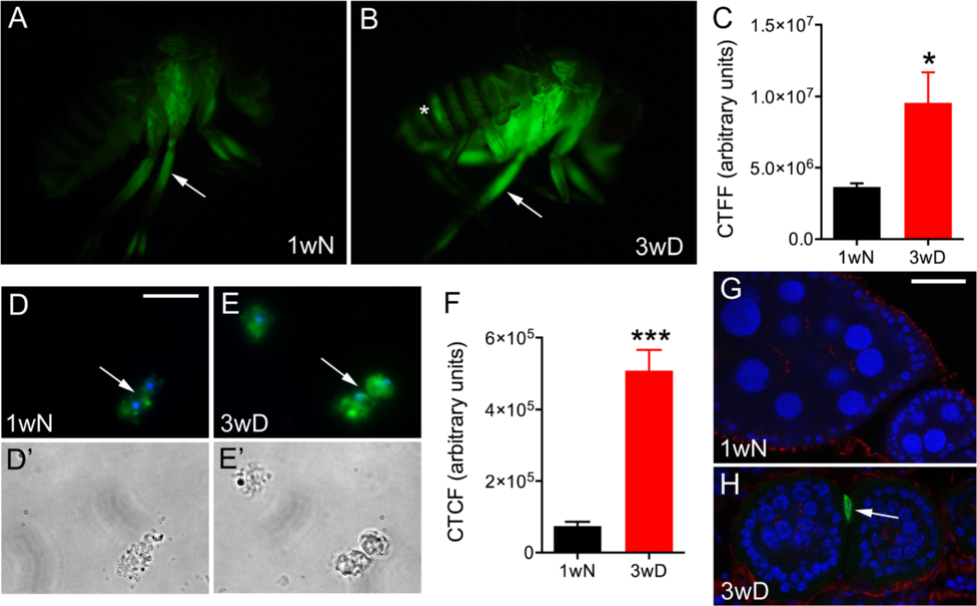
Tissue specific JAK/STAT signaling in diapausing flies. The Stat92E reporter *10xStat92E-GFP* expression in the whole body is compared between control flies (1wN) (**a**) and flies in diapause conditions (3wD) (**b**) and shows an increased fluorescence in 3wD, as quantified in (**c**) with corrected total fly fluorescence (CTFF). The statistical significance was verified by Welch’s unpaired *t*-test, * *p* < 0.05, *n* = 6 (error bars show standard error of the mean). In (**a**) and (**b**) arrows point to hemocytes that accumulated in the legs of the fly, which largely contributes to the fluorescence signal, and the asterisk in B indicates GFP signal in the ovaries. **d** and **e** Hemocytes exhibit a strong accumulation of the *10xStat92E-GFP* signal upon diapause, 3wD (**e**) compared to controls, 1wN (**d**). In these figures green label is *10xStat92E-GFP*, and blue label hemocyte nuclei stained with DAPI. **E**´ and **D**´ The corresponding hemocytes displayed in bright field. The scale bar corresponds to 20 μm. **f** Corrected total cell fluorescence (CTCF) for diapause and control hemocytes is calculated as an average from *n* = 15, statistical significance verified by Welch’s unpaired *t*-test, ***, *p* < 0.001. Error bars show standard error of the mean. **g** and **h** Portions of ovaries from control (1wN) and diapausing (3wD) flies. In diapausing flies there is an increased expression of the *10xStat92E-GFP* reporter (green) in polar cells of ovaries (arrow in **h**), which is not detectable in directly developing ovaries of control flies (**g**). Blue label, nuclei stained with DAPI; red label phalloidin-TRITC. Scale bars: D, E 50 μm, G, H 25 μm

The upregulation of *TotA, vir-1* and *Tep2* during diapause suggest a possible role of JAK/STAT in enhancing the stress and immune defense. This is supported by the upregulation of other Turandot genes, including *TotA*, *TotC*, *TotM*, and *TotX* (see Additional file 6: Table S3). Taken together, our findings indicate that the JAK/STAT pathway may regulate different target genes in a tissue-specific manner in diapausing flies. To test this hypothesis we kept flies expressing a *10xStat92E-GFP* reporter [76] under diapause and normal conditions and screened for tissue-specific activation of JAK/STAT signaling. Strong GFP fluorescence was detected in specific thoracic regions, including thorax, legs, and ovaries of diapausing flies (Fig. 6a,b). The overall GFP expression increased significantly during diapause (Fig. 6c)

The strong GFP signal of the *10xStat92E-GFP* reporter in the whole fly body appears to derive mainly from hemocytes (Fig. 6d-f). Adult hemocytes are mostly sessile and tend to accumulate in legs and thorax close to the halteres [92]. This immune tissue specific regulation might explain why we detected predominantly stress-related and immune-response targets of JAK/STAT in our microarray. It has been shown that JAK/STAT-signaling regulates humoral defense factors, including several ones produced by hemocytes [81]. We noted an upregulation of *Tep2, 3* and *4* (see Fig. 5), which resemble both the vertebrate complement factors and members of the α-macroglobulin family of protease inhibitors, and are predominantly expressed by hemocytes [81, 87]. A more detailed analysis of the ovaries revealed that the GFP signal is localized to specialized follicle cells (polar cells) and close to stem cells (Fig. 6g, h). It is known that JAK/STAT signaling is critically important for early differentiation of follicle cells and proper germ line cell encapsulation during *Drosophila* oogenesis [88, 93, 94], as well as subsequent reduction of polar cell number [95] and border cell migration which occurs during stage 9 [96]. Oogenesis in diapausing females is in most cases arrested at previtellogenic stages (stage 7 and earlier) [20], when strong JAK/STAT signaling in polar cells is active and necessary to keep egg chamber morphology stable.

### Innate immunity: the Toll pathway is activated

**Fig. 7:**
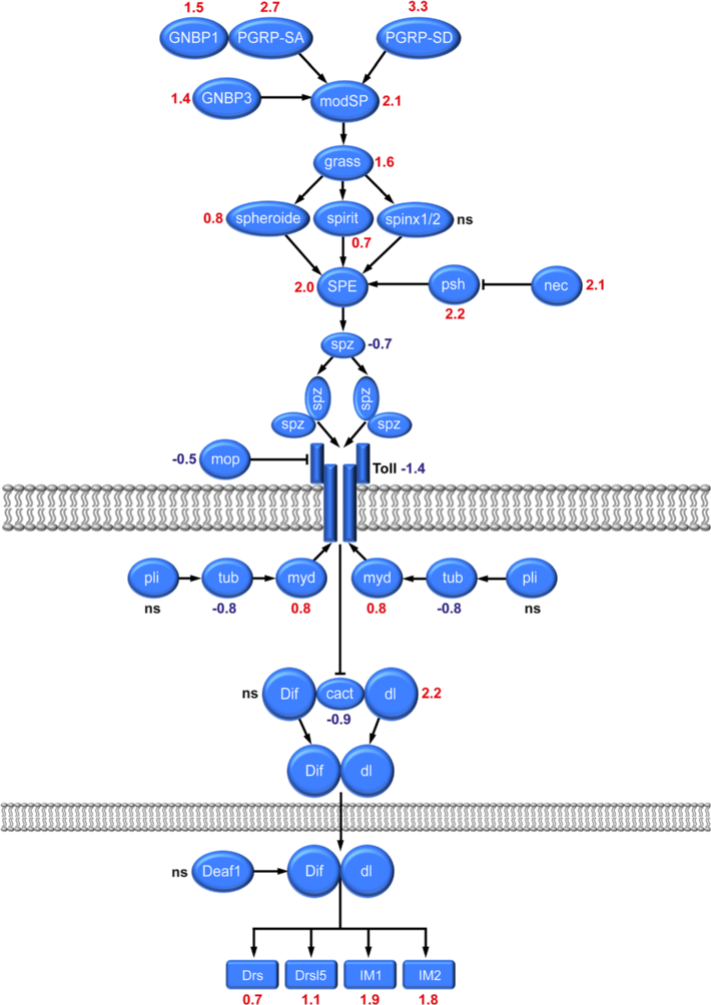
Altered gene expression in Toll (*Tl*) signaling pathway during diapause. The scheme displays a generalized assembly (regardless tissue specificity) of relevant genes in the Toll signal pathway. Transcript levels (logarithmic fold change, LogFC) are given in red for upregulated, blue for downregulated and black for no significant change (ns; LogFC close to 0). The acronyms are listed in Additional file 6: Table S3 where also references and details of gene/protein functions are given. The Toll pathway is activated in diapausing flies as seen from increases of target genes such as *Drosomycin* (*Drs*), *Drosomycin-like 5* (*Drsl5*), *IM1* and *IM2*. Also several extracellular members of the Toll-activating proteolytic cascade were upregulated. The signaling scheme is based on [97]

**Fig. 8:**
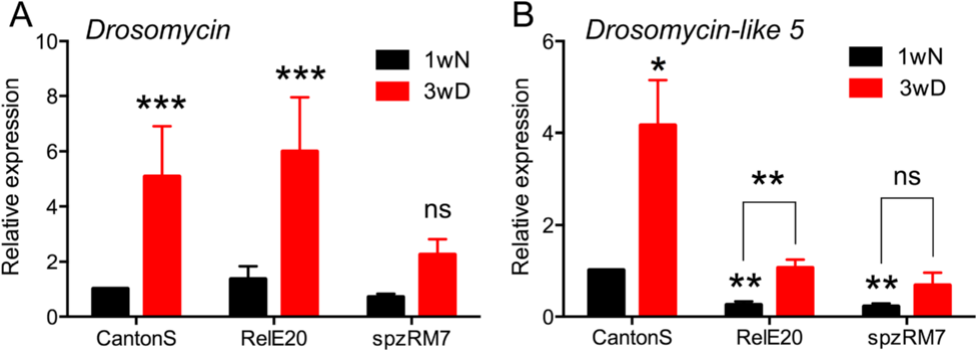
*Drosomycin* (*Drs*) and *Drosomycin-like 5* (*Drsl5*) transcription during diapause is regulated by Spätzle and Toll signaling. Upregulation of *Drosomycin* (**a**) and *Drosomycin-like 5* (**b**) is significant in the control genotype (Canton S), as well as in a mutant in a component of the Imd pathway, the transcription factor Relish (*RelE20*). Conversely transcription of *Drs* and *Drsl5* was blocked in a mutant in the Toll ligand Spätzle (*spzRM7*). Significance compared to one-week-old Canton S flies (1wN) was verified by ANOVA, followed by Tukey test (ns, not significant; * *p* < 0.05; ** *p* < 0.01; *** *p* < 0.001). Error bars show standard error of the mean, *n* = 3

Analysis of the array data confirmed our previous results [20] that *Drosomycin* as well as other *Drosomycin-like* genes exhibit increased expression in flies that have entered diapause. *Drosomycin* is considered a canonical target of Toll (Tl) signaling (see [97]). A summary diagram is shown in Fig. 7 and further details on immune genes are given in Additional file 6: Table S3. We found significant and strong upregulation for two additional targets of Tl signaling, namely *Immune induced molecule 1* (IM1) and *Immune induced molecule 2* (IM2) (see [98] and Fig. 7). A detailed pathway analysis revealed upregulation of genes for Peptidoglycan recognition proteins (PGRPs) and Gram-negative bacteria binding proteins (GNBPs), which bind to immune elicitors and activate an extracellular proteolytic cascade ultimately leading to proteolytic activation of Spätzle (encoded by *spz*) to its active form. Several extracellular members of the Tl-activating proteolytic cascade were upregulated upon diapause (see Fig. 7). Since both the serine protease *Persephone* (*psh*), which is part of the Spätzle-activating cascade [99], but also its inhibitor *Necrotic* (*nec*), were induced, activation of Tl signaling occurs most likely downstream of *ModSP/Grass,* a multifunctional member of the cascade. Despite a slight downregulation of Tl itself and its ligand Spätzle (encoded by *spz*), we could clearly show that the induction of both *Drs* and *Drsl5* is regulated via Spätzle, since *spz* mutants failed to induce both peptides, while flies mutant in the transcription factor *relish* (*rel*), which is part of the IMD (*Drosophila* immune deficiency) pathway show no such difference (Fig. 8a and b). Downstream of Tl-signaling during diapause the combined upregulation of the transcription factor dorsal (*dl*) and the downregulation of the inhibitor cactus (*cact*), leads to increased transcription of *dl* (and possibly *dif*) target genes.

### The ERK/MAPK pathway is downregulated

The extracellular signal-regulated kinase/mitogen-activated protein kinase (ERK/MAPK) signaling cascade regulates cell proliferation, differentiation and survival [100, 101] and we detected a downregulation of key components of this pathway. ERK/MAPK signaling can be triggered by ligands binding to receptor tyrosine kinases (RTKs) with subsequent Ras activation at the plasma membrane or via Ras induction from intracellular sites [101]. As seen in Additional file 8: Figure S4 downregulation of the whole MAPK signaling cascade in diapausing flies is supported by decreased expressions of all kinases, including MAPKKK/Raf1 (encoded by *phl*), MAPKK/MEK (encoded by *Dsor1*) and MAPK/ERK (encoded by *rl*). In addition we found diminished expressions of almost all positive regulators in the pathway, participating in Ras (*βggt-I, Fnta, Hmgcr*)*,* Raf1 (*ave, ksr, cnk, Cdc37, 14-3-3ε*), MEK (*gfzf)* and ERK (*mago, Fip1, Cdk12, eIF4AIII*) activation [100]. Further details on specific genes are provided in Additional file 6: Table S3. At the receptor level we noted an activation of genes encoding the RTK Sevenless and its ligand Boss in the diapausing flies. Thus, there might be a role of Boss in regulation of carbohydrate and lipid metabolism during diapause, similar to the one found during larva to pupa transition [102]. Possibly downregulation of MAPK signaling is of general importance during diapause, while its activation is responsible for the initiation of development following diapause termination in several insect species [103–105].

### JNK signaling is activated in diapause

The Jun-N-terminal Kinase (JNK) signaling pathway is an evolutionarily conserved stress-activated protein kinase pathway that is induced by a range of intrinsic and extrinsic stressors [106, 107]. JNK signaling is also involved in developmental and metabolic regulation, immune responses, as well as cell death and lifespan extension [106, 108]. In *Drosophila* JNK signaling involves a conserved core of kinases (JNKKK-JNKK-JNK) and a mitogen activated protein kinase (MAPK) pathway, and can be loosely classified into “canonical” and “non-canonical” signaling pathways [107]. Our transcriptome data indicate a moderate general activation of the “non-canonical” JNK signaling pathway in diapausing flies.

**Fig. 9:**
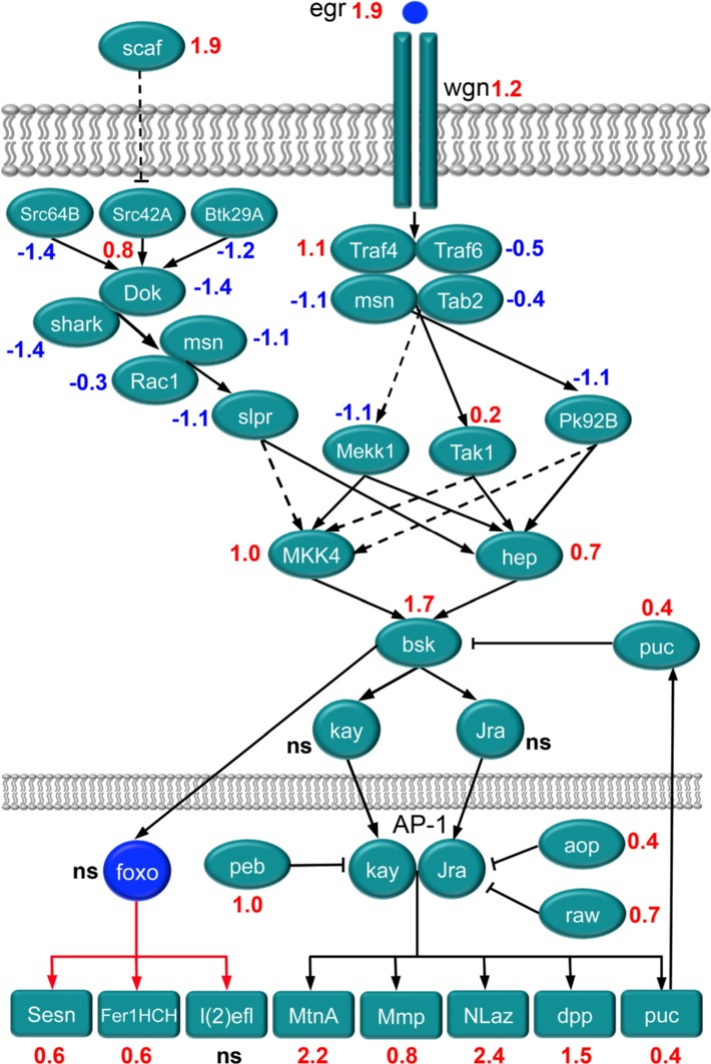
Altered gene expression in the JNK signaling pathway during diapause. This scheme displays a generalized assembly (regardless tissue specificity) of relevant genes in the JNK signal pathway. Transcript levels (logarithmic fold change, LogFC) are given in red for upregulated, blue for downregulated and black for no significant change (ns; LogFC close to 0). The acronyms are listed in Additional file 6: Table S3 where also references and details of gene/protein functions are given. At the whole organism level diapausing flies demonstrate a moderate activation of JNK signaling as judged from an elevated expression of some target genes. There is also enhanced expression of JNK-responsive genes, known to be under FOXO (IIS) transcriptional control (*Sesn* and *Fer1HCH*)

We assembled an overview of the JNK signaling cascade in *D. melanogaster* (Based on [106, 107]) with associated transcript changes induced by diapause (Fig. 9). Details on genes are provided in Additional file 6: Table S3. The “canonical” JNK pathway seems to be downregulated in diapausing flies, since we detect decreased expression of transcripts of the main upstream acting molecules, like non-receptor tyrosine kinases of the *Src* oncogene family (*Src64B* and *Btk29A,* but not *Src42A*) and the kinase *shark,* together with its adaptor protein *Downstream of kinase* (*Dok)*. Further components of the “canonical” pathway were also downregulated, including the specific JNKKK *Slipper* (*slpr*), together with the kinase *Misshapen* (*msn*), as well as the main activator *Rac1* (a small GTPase)*.*

In the “non-canonical” JNK pathway a ligand, the tumor necrosis factor homolog Eiger (*egr/TNF*) [109] and a receptor, Wengen (*wgn/TNFR*) [110] have been identified, both of which were upregulated in diapausing flies (Fig. 9). The activation of “non-canonical” JNK signaling in diapause is further suggested by elevated transcriptional levels of the main target genes of the transcriptional complex AP-1, including a *Drosophila* member of transforming growth factor-β (TGF-β) family *decapentaplegic (dpp)* and *puckered* (*puc*), which encodes a JNK-specific phosphatase (Fig. 9). Previous studies of diapausing *D. melanogaster* actually revealed that upregulation of the JNK target *dpp* varies in Australian and North American clines [26, 111]. Among JNK signaling responsive genes, upregulated in diapausing flies, we also found the homolog of vertebrate profilin *chickadee (chick), matrix metalloproteinase (Mmp1),* free radical scavenger *Ferritin 1 heavy chain (Fer1HCH),* the antioxidants *metallothionein A (MtnA) and Sestrin (Sesn)* and the lipocalin *Neural Lazarillo (NLaz)*.

Moderate activation of JNK signaling results in increased stress tolerance and extended lifespan [106], due to increased expression of cytoprotective JNK target genes in various tissues [112, 113]. This mechanism is balanced by an antagonism between JNK and IIS signaling pathways that regulate FOXO in opposite ways [108]. The same mechanism may be involved in *Drosophila* diapause leading to extended lifespan and increased stress tolerance [9, 15, 20]. Taken together our findings indicate a moderate general activation of “non-canonical” JNK signaling in diapausing *D. melanogaster* that could contribute to extended lifespan.

### Neuropeptides and peptide hormone signaling are selectively affected

In *Drosophila*, as in other animals, neuroendocrine peptides are central to the regulation of development, reproduction, physiology and behavior [114, 115] and at least insulin-like peptides and AKH have been implicated in regulation of longevity [28, 32, 71]. Thus, we screened for diapause-induced changes in expression of genes encoding neuropeptide/peptide hormone precursors and their G-protein coupled receptors (GPCRs).

The transcripts of 23 neuroendocrine peptide precursors were significantly upregulated in diapausing flies (Table 1). The only precursor transcript that was significantly down-regulated was *dilp8*, proposed to be expressed in ovaries (FlyAtlas [116], ModENCODE [117]). For four of the peptide ligands also the corresponding GPCR transcripts were significantly upregulated: those of AKH, corazonin (CRZ) diuretic hormone 44 (DH_44_), and leucokinin (LK). Transcripts of five peptide GPCRs were upregulated, but with ligand levels unchanged: the DH_31_-receptor 1 (DH_31_-R1), the capability (CAPA) receptor (capaR), DLGR1 (ligand is the dimeric protein GPA2/GPB5) and the neuropeptide F receptor (NPFR). Four of the peptide hormone signaling pathways with increased transcripts (capaR, DH_31_-R1, DH_44_, LK) are known to regulate diuresis by action on the Malpighian tubules, and the NPFR is expressed in this tissue [118]. Furthermore, in mosquitos DLGR1 is involved in hindgut regulation of ion and water balance [119]. Some of the upregulated peptides modulate feeding [allatostatin A (AstA), CCHamide2, short neuropeptide F (sNPF), NPF, drosulfakinin (DSK)] and others (CRZ, sNPF, tachykinin) may regulate stress responses [115, 118, 120–122]. Finally, five of the peptides (AstA, CRZ, CCHamide2, sNPF, tachykinin) are known to act on the insulin producing cells of the brain and thus modulate IIS [122–124]. In summary, peptidergic signaling appears altered in pathways involved in regulation of feeding, carbohydrate and lipid metabolism, IIS, stress responses, diuretic functions and longevity.

### Juvenile hormone, ecdysone and monoamine signaling is affected by diapause

Since monoamines, ecdysone (Ecd) and juvenile hormone (JH) have been implicated in diapause, reproduction and stress responses in several insects (see [8, 25, 27, 125–127]) we assembled transcript data for relevant genes in these signaling pathways (Table 2). Several genes associated with serotonin, dopamine and octopamine signaling displayed increased expression (Table 2). Whereas dopamine signaling may be confined to the CNS in *Drosophila*, serotonin and octopamine are also known to have peripheral targets [128].

**Table 2:** Hormonal and monoamine signaling components affected by diapause

Gene transcript	Role (main enrichment^a^)	CG number	Log FC
*Monoamine signaling*			
5-HT2A (serotonin receptor 2)	Serotonin signaling (salivary gland, eye)	CG1056	2.2
SerT (serotonin transporter)	Serotonin signaling (CNS, eye)	CG4545	1.4
Ddc (Dopa decarboxylase) +2.82 CG10697	Dopamine biosynthesis (CNS)	CG10697	2.8
DAT (Dopamine transporter) +1.7 CG8380 + 1.35 CG3856	Dopamine storage (CNS)	CG8380	1.7
DopR2 (Dopamine receptor 2) +1.15 CG18741	Dopamine signaling (CNS)	CG18741	1.1
DopEcdR (Dopamine/Ecd receptor) +2.87 CG18314	Dopamine ecdysone signaling (CNS)	CG18314	2.9
Dat (Dopamine N acetyltransferase) +1.35 CG3318	Dopamine inactivation (CNS, gut)	CG3318	1.4
Tdc1 (Tyrosine decarboxylase 1) +1.56 CG30445	Octopamine/tyramine biosynt. (gut)	CG30445	1.6
Oamb (octopamine receptor)	Octopamine (OA) signaling (CNS)	CG3856	1.4
Oct-TyrR (OA-Tyramine receptor) +1.84 CG7485	Octopamine/tyramine signal. (CNS)	CG7485	1.8
*Ecdysone signaling*			
Spo (Spook) Cytochrome P450	Ecdysone (Ecd) biosynthesis (ovary)	CG10594	−1.6
Eo (ecdysone oxidase) +2.44 CG9504	Ecd metabolism (renal tubules; RT)	CG9504	2.4
Usp (ultraspiracle)	Ecd receptor partner (ovary)	CG4380	−1.3
Cyp18a1 Cytochrome P450^b^	Ecd inducible (fat body, spermatheca)	CG6816	1.6
Eip63F1 (Ecd induced protein 63 F1)^c^	Ecd inducible protein (CNS, gut, RT)	CG15855	2.0
Eip75B (Ecd induced protein 75B)^b^ + 1.1 CG8127)	Ecd inducible protein (ubiq.)	CG8127	1.1
Eip74EF (Ecd induced prot. 74EF) +1.67 CG32180	Ecd inducible protein^d^ (brain, crop)	CG32180	1.7
Ftz-f1 (ftz transcription factor 1)	Ecd and JH^e^ inducible gene (ovary)	CG4059	−1.9
ImpL3 (ecd inducible gene L3)	Ecd inducible gene (midgut)	CG10160	2.4
Br (Broad)	DNA-binding, Ecd response (CNS)	CG11491	−1.5
ecd (ecdysoneless)	Ecd biosynthesis ? (CNS, ovary)	CG5714	−1.1
*Juvenile hormone signaling*			
Jhamt (JH acid methyltransferase)	JH biosynthesis (brain)	CG17330	1.8
Jheh1 (JH epoxide hydrolase 1)	JH catabolic process (fat body, gut)	CG15101	1.3
Jeheh3 (JH epoxide hydrolase 3)	JH catabolic process (gut)	CG15106	1.2
Jhedup (JH esterase duplication)	Carboxylesterase activity (head)	CG8424	3.2
Jhi1 (JH-inducible protein 1)	endoribonuclease activity (ubiq.)	CG3298	−1.5
jhi26 (JH-inducible protein 26)	Protein kinase-like (renal tubules)	CG3767	2.9

*Notes:* These transcripts are significantly affected more than two-fold (>LogFC 1)

^a^Adult expression (according to FlyAtlas)

^b^Read-out for increased ecdysone or 20-hydroxy ecdysone signaling during development [129, 130]

^c^EF-Hand 1, calcium-binding site

^d^may be involved in autophagy

^e^JH, juvenile hormone

Ecd signaling may be upregulated after three weeks of diapause since the genes *Eip75B* (*E75B*) and *Cyp18a1*, often used as proxys for elevated Ecd titer [129, 130], display increased transcript levels (Table 2). Also the Ecd-induced genes, Eip74EF and ImpL3 as well as the Dopamine/Ecd receptor (DopEcdR) are upregulated. Prothoracicotropic hormone (PTTH), a peptide known to induce Ecd biosynthesis [131] is also significantly upregulated in diapausing flies (Table 1). However, genes encoding the biosynthetic enzymes *Spook, ecdysoneless,* the Ecd receptor partner *Ultraspiracle,* the DNA-binding *Broad*, and the Ecd-induced transcription factor *Ftz-f1* are downregulated and *Ecd oxidase* upregulated during diapause. As shown in Table 2 several of the downregulated genes, including that of the PTTH receptor *Torso* [132] (see Table 1), are normally enriched in ovaries, suggesting a possible tissue specific response in adult flies.

In the JH signaling pathway genes in catabolic processes are upregulated, whereas one JH-inducible gene is upregulated (jhi1) and one is downregulated (jh26). Thus it is not clear from our transcript data how JH signaling is affected by three weeks of diapause at the organism level. In summary, it is likely that the dynamics of JH and Ecd signaling is most drastic during the initial phases of diapause and thus our data reflect a later, steady state transcriptional snapshot. Therefore, we may miss transcript changes reflecting critical transient hormone pulses or decreases, as well as feedback mechanisms.

### Altered transcripts of numerous genes implicated in aging and lifespan extension in *Drosophila*

**Fig. 10:**
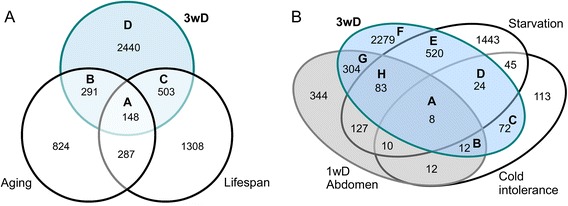
Venn diagrams comparing differentially regulated genes in genome-wide transcriptome studies of *D. melanogaster*. **a** Venn diagram comparing differentially regulated genes after 3 weeks of diapause (3wD, blue area; our study) with genes responding to aging [136] and a list of candidate genes affecting lifespan [135]. In Additional file 10: Figure S5 we show the results of Overrepresentation test of GO terms, performed in PANTHER for genes altered in dormancy in comparisons in groups A-D. **b** Comparisons with starvation, one-week diapause (abdomen) and cold tolerance. The blue area displays the number of differentially regulated genes after 3 weeks of diapause (3wD; our study). The grey area shows number of genes regulated in abdomens of flies after one week of diapause (1wD Abdomen) [140]. The other two sets contain genes regulated due to cold intolerance [141] and starvation [143]. In Additional file 11: Figure S6 we present a gene set enrichment analysis (GSEA) of Gene Ontology (GO) terms for comparisons among the four genome-wide transcription studies for each of the fields A-H in 10B

We compared our list of diapause induced transcript changes with a set of genes from the GenAge database [133, 134]. Out of 136 genes, which are known to influence longevity in *D melanogaster*, we found 100 to be altered also during dormancy (Table 3, Additional file 9: Table S4). Whereas genes with pro-longevity effects are mostly strongly upregulated, anti-longevity genes are downregulated as a response to dormancy. This is in line with the drastic extension of lifespan with reduced senescence shown in diapausing flies [20]. Performing a broader comparison, we revealed that about 28 % of the genes in our study (Fig. 10a, for details see Additional file 9: Table S4) have previously been recorded as part of a transcriptional response to aging [7, 135, 136], or were identified as candidate genes affecting longevity [33, 135]. According to the Panther GO overrepresentation test the majority of these shared genes do not have mapped GO terms, however some are involved in actomyosin organization and meiotic and mitotic division (Additional file 10: Figure S5).

**Table 3:** Genes shared between 3 weeks diapause and aging in *Drosophila*

Symbol	Gene name	3wD (logFC)	Longevity effect
ry	rosy	2.4	pro
Nlaz	Neural Lazarillo	2.38	pro
to	takeout	2.21	pro
Zw	Zwischenferment	2.18	pro
elav	embryonic lethal abnormal vision	1.83	pro
bsk	basket	1.69	pro
GstS1	Glutathione S transferase S1	1.68	pro
Nf1	Neurofibromin 1	1.63	pro
CG1623	hebe	1.54	pro
Mlp84B	Muscle LIM protein at 84B	1.45	pro
CG11546	kermit	1.43	pro
CG8846	Thor	1.3	pro
Ilp6	Insulin-like peptide 6	1.17	pro
cher	cheerio	1.13	pro
rut	rutabaga	1.01	pro
srl	spargel	−1.06	pro
bam	bag of marbles	−1.18	pro
Daxx	Daxx-like protein	−1.4	pro
POSH	Plenty of SH3s	−1.42	pro
CG5671	Pten	−1.71	pro
Mt2	Methyltransferase 2	−2.14	pro
Hsp26	Heat shock protein 26	−2.89	pro
Hsp27	Heat shock protein 27	−3.15	pro
CG6284	Sirt6	−3.41	pro
a-Man-Ia	alpha Mannosidase I	2.01	anti
Ilp2	Insulin-like peptide 2	1.9	anti
E(z)	Enhancer of zeste	−1	anti
snz	snazarus	−1.2	anti
Edem1	ER degradation enhancer mannosidase alpha-like 1	−1.35	anti
kuk	kugelkern	−1.36	anti
LBR	Lamin B receptor	−1.91	anti
CG6824	ovo	−1.98	anti
esc	extra sexcombs	−2.44	anti
Hsp22	Hsp22	−1.13	both

*Notes:* During diapause 34 genes out of 136 hits for aging genes in GenAge database were found to be significantly regulated (q < 0.05, |logFC| > 1). Comparison with the GenAge database [133, 134] http://genomics.senescence.info/genes/

We find that this overlap with several aging studies is not only observed at the level of gene lists, but also in the occurrence of affected signaling pathways and GO terms. Using the *Drosophila melanogaster* genetic reference panel (DGRP) for a genome-wide analysis of single nucleotide polymorphisms (SNPs) influencing lifespan a number of genes were identified with roles in processes such as carbohydrate metabolism, TOR signaling, proteolysis, cell death and others [33]. Of the top ranked genes identified in that study we also identified *TOR, slif, mipp2, Thor, sima, dm* of the TOR pathway, and *brummer* (and *Thor*) of the IIS pathway, as well as the protease *nephrilysin1* (*Nep1*) and the organic cation transporter (*Orct*), possibly downstream of S6K in TOR signaling (see Fig. 4 and Additional file 3: Dataset S1, Additional file 9: Table S4).

Increasing S-adenosylmethionine (SAM) synthesis by FOXO-dependent glycine N methyltransferase (Gnmt) extends the lifespan in *Drosophila* and thus overexpression of *Gnmt* increases longevity, cooperatively with dietary restriction and lowered IIS [137]. We see a 6.3 LogFC (increase) in *Gnmt* in three week diapausing flies (Additional file 3: Dataset S1, Additional file 9: Table S4). Another gene implicated in *Drosophila* lifespan extension is *Tequila* a multiple-domain serine protease known to be upregulated during infection [138]. These authors showed that knockdown of *Tequila* in insulin producing cells increases longevity, probably due to decreased systemic IIS. In our transcript data *Tequila* is significantly upregulated during diapause (1.7 LogFC).

Finally, it was noted that aging is associated with alterations of genes involved in responses to light and expressed in the eye [139], and we found a few genes associated with phototransduction significantly upregulated: *NinaC* (CG5125, 2.1 LogFC), *InaE* (CG33174, 1.6 LogFC), *Rhodopsin 3* (CG10888, 1.2 LogFC) and *Gβ5* (CG10763, 2.15 LogFC). Thus, apart from components of the IIS and TOR pathways, a number of single genes with important roles in extending lifespan have been implicated in various screens and are also seen in our gene list from diapausing flies.

### Comparisons with other genome-wide investigations of dormancy and stress

Despite the high level of complexity of transcriptional changes in fly diapause, as reflected by the large number of affected genes in our analysis, there are certain commonalities with gene regulation found in other stress syndromes, including other forms of dormancy in animals. Especially comparisons at a gene ontology level show considerable conservation.

First, we compared our gene list with previously published genome-wide transcription studies in *D. melanogaster*, where relevant physiological phenotypes were tested. Not surprisingly, we found considerable overlap with transcripts identified in a study focusing on egg development during *one week* in diapause conditions [140]. Almost 50 % of the altered genes of that genome-wide transcription study were changed also in our analysis (Fig. 10b). According to the GO analysis, these genes are mostly involved in DNA replication and repair, chitin based cuticle attachment to epithelium, anatomical structure development, regulation of mitosis and maintenance of RNA localization (see Additional file 11: Figure S6, groups G and H). Note that the egg development study only analyzed the transcriptome of the abdomen, whereas our study covers transcriptional changes in the whole fly. Thus, our work includes additional genes expressed in the CNS and other tissues in the head and thorax, likely to be important in association with the diapause syndrome.

After three weeks in diapause at 11 °C the flies seem well adapted to cold conditions. Thus we compared our data to a genome-wide analysis of transcriptional changes in a cold-sensitive fly strain compared to its cold resistant siblings [141]. We find that 40 % of the genes are shared between these studies (Fig. 10b). The majority of the genes are regulated in opposite directions compared to our 3wD flies. The GO terms common for the shared genes are response to heat, immune response (covers part of the Toll pathway and stress response pathway JAK/STAT) and response to oxidative stress (see Additional file 11: Figure S6, group C). In this context, the gene *frost* (CG9434) is among the most upregulated in our study (LogFC 7.4; Fig. 2) and is known to be essential for cold tolerance [142].

During diapause, food intake of flies is strongly reduced [20]. Indeed, a comparison with a study of starved flies [143] reveals about 30 % shared genes and GO terms, such as inhibition of cell division, changes in biosynthesis, carbohydrate catabolism (for GO term enrichment analysis see Additional file 11: Figure S6, groups D and E) and downregulation of TOR pathway response genes (e.g. *ash2*). A comparison with abdominal genes after 1wD [140] only highlights 10 % shared genes (Fig. 10b). Starvation usually results in many pathologies, eventually leading to increased mortality, whereas during diapause flies still feed at a reduced rate, aging is slowed down and flies display extended lifespan [20]. Possibly the adult diapause is more similar to food restriction [144]. Indeed, a comparison to transcriptome changes after food restriction show 50 % of the transcripts we identified in our analysis (Additional file 12: Dataset S2). However, most genes exhibit much higher fold change in diapause than upon food restriction. The shared genes are mostly connected to cell cycle and gene expression (Additional file 13: Dataset S3). Surprisingly, 120 genes were found to be regulated in opposite direction; these mostly comprise carbohydrate metabolism, as verified by GO term enrichment analysis (Additional file 13: Dataset S3), and a comparison of GSEA of KEGG pathways between ours and the food restriction study (compare Additional file 4: Table S1 with [145]). Whereas in diapause we found many upregulated genes in starch, sucrose, galactose metabolism, as well as pentose cycle, those pathways are mostly downregulated upon food restriction. Another discrepancy we found is in expression of cytochromes p450, which are highly upregulated upon diapause.

Next, we compared our transcriptome analysis to studies on *D. melanogaster* where the focus was on mapping genetic variation that could be implicated in diapause, revealing many interesting similarities. As already mentioned, the importance of insulin-regulated genes such as *dp110* for diapause has been found also in genetic studies (e.g. [24]), and *coach potato,* previously identified as a candidate gene for climatic adaptations in *D. melanogaster* [146] was differentially expressed in our transcriptome. Tauber et al. [147] provided evidence that a mutation in the clock gene *timeless* has spread in Europe over the last 10 000 years, and suggested a link to an enhancement of diapause (see also [148]). Notably, *timeless* was one of the most strongly differentially regulated genes in our study. Finally, a genome-wide sequencing study [26] comparing populations along the North American east coast found that many of the most strongly differentiated genes along the cline of increased diapause intensity northwards are involved in functional pathways indicated also by our transcriptome study, i.e. insulin/TOR, JAK/STAT, immunity and circadian rhythm pathways. In all, this concordance between studies of different types suggests that the same genes are targeted by genetic adaptation and transcriptional responses, and indicates that an understanding of the pathways involved in diapause is emerging.

What about other species? Only a limited number of transcriptome studies on reproductive diapause in other insect species are available so far, and these usually identify only a small number of significantly changed genes. In a comparison with a targeted mini-array performed on *Drosophila montana,* that live in temperate regions and has a photoperiodically regulated reproductive diapause [149], we found three genes out of 17 to be regulated in the same manner in *D. melanogaster* (Additional file 14: Dataset S4). These include upregulated *couch potato*, and downregulated *CG7650* (encoding a phosducin-like protein) and *Hsp70/Hsp90 organizing protein homolog*. Transcriptional changes in *Hsp70* and *Hsp90* during diapause have been described for several insect species [10] and these molecular chaperons may be involved in increased resistance to stress. In our analysis of *D. melanogaster* four small heat shock proteins (Hsp22, 23, 26 and 27) were found downregulated (Additional file 3: Dataset S1). Three of these are normally enriched in the ovaries. In the Asian temperate *Drosophila* species *D. triauraria Drosomycin* and *Drosomycin-like* genes were found to be strongly upregulated during diapause, whereas other immune response genes like *Drosocin* and *Defensin* were not [150]. This is in line with our findings, that *Drosomycin* and *Drosomycin-like* genes are upregulated via the Toll-pathway, whereas the imd pathway is not influenced upon diapause. Another study in *D. triauraria* reveled allelic variations in *timeless* and *cryptochrome* genes, which influence the incidence of diapause [151]. This might be related to our findings that *timeless* isoforms are regulated differentially upon diapause in *D. melanogaster*.

More distantly in dipteran insects, we made a comparison with candidate genes of diapausing mosquitos, *Culex pipiens*, revealed from suppressive subtractive hybridization [152]. We identified 5 out of 40 genes that are in common with our study (Additional file 14: Dataset S4). These include the extracellular matrix component *Multiplexin*, *CG34227* (encoding a secreted protein with unknown function), *Mediator complex subunit 14* (encoding an RNA polymerase II complex subunit) and two heat shock proteins (*Hsp23* and *Hsp27*), which are downregulated in diapausing mosquitos, but upregulated in diapausing fruitflies. A variable Heat shock protein expression (species specific or transcript specific), has been described previously for many insect species, and is known to vary among species with reproductive diapause, from flies to mosquitoes and beetles [10].

More general comparisons of KEGG or GO analyses performed on insects displaying different forms of diapause (including other developmental stages), and also the dormant dauer state in *C. elegans* reveal that similar signal pathways are affected, such as IIS, TOR, stress responses, carbohydrate and lipid metabolism [62, 153–156]. However, so far limited transcriptional similarities have been detected across species, and it has even been suggested that there may be many transcriptional strategies for producing similar dormancy phenotypes [62]. Possibly, the same genetic and physiological modules are involved across taxa, but the details of how they interconnect may differ, leading to unexpected variation in the expression of specific genes when extrapolating between species.

## Conclusions

The drastically extended lifespan and diminished senescence associated with adult diapause is a likely to be caused by multiple factors that increase resistance to the deleterious effects of stress and aging. Indeed, we found that a substantial portion of the genes and signal pathways affected by *D. melanogaster* diapause is shared with the ones revealed in studies of aging and extended lifespan, and also include pathways regulating development, detoxification, as well as stress and immune responses [2, 4, 7, 135, 136].

Diapause induces a massive alteration of gene expression in *D. melanogaster* with more than 4500 differentially regulated genes. Gene Ontology, GSEA and KEGG analysis provide clues to processes that are affected by diapause. These include metabolic processes (energetic and storage metabolism), cell communication, developmental processes, neuronal function, reproduction, lysosome pathway, transcription, translation, protein processing, clock system, drug metabolism and detoxification. Our manual annotation of transcripts in signaling pathways reveals downregulation of IIS, TOR and MAPK signaling and upregulation of JNK and Toll immune signaling, as well as tissue specific upregulation of JAK/STAT signaling in hemocytes. All these pathways are likely involved in the diapause phenotype with reduced food intake, diminished metabolism, increased nutrient stores, arrested vitellogenesis, and increased stress resistance. Several of the pathways have also been implicated in extended lifespan and diminished senescence [2, 7, 32, 33, 135, 136]. Furthermore, we find that many genes whose expressions are affected in diapausing flies are shared with ones found in *D. melanogaster* exposed to restricted diet or cold [141, 144]. A portion of the transcript changes probably reflects the transition from reproduction to survival mode, characteristic of adult diapause. Thus, experimentally induced diapause is a suitable means to study regulation of processes underlying aging related phenomena.

Comparisons with other insects that display adult reproductive diapause such as *D. montana* and the mosquito *Culex pipiens*, where limited transcriptome analyses have been performed [149, 152], revealed some overlap, though restricted by the small number of transcripts assessed in these studies. However, general comparisons of transcript changes in components of signal pathways by KEGG or GO analyses suggest similarities between reproductive diapause in *Drosophila* and dormancy in other insects and *C. elegans*, including IIS and TOR signaling, stress responses and metabolism, as well as energy storage [62, 153–156]. This comparison can be extended to mammals, where the hibernation phenotype involves altered metabolism and TOR signaling [157, 158]. Taken together our data provide an organism-wide and detailed analysis of transcriptional changes induced by diapause, and shows the power of *D. melanogaster* as a model to analyze genetics of dormancy and its effects on lifespan and aging.

## Methods

### *Drosophila* strains and diapause induction

*Drosophila melanogaster* of the *Canton S* strain, obtained from the Bloomington Drosophila Stock Center (BDSC, Bloomington, IN) was used in the microarray analysis. For analysis of transcriptional regulation of immune response genes during diapause we also tested *Rel*^*E20*^ [159] and *spz*^*rm7*^ [160] mutants kindly provided by Bruno Lemaitre (Lausanne, Switzerland). Furthermore, for monitoring activity in the JAK/STAT pathway we used *w*^*1118*^*; 10xStat92E-GFP* [76] reporter flies from BDSC. For diapause induction we followed the protocol described in our previous study [20].

### RNA isolation and sampling

As our experimental group for microarray analysis we used *Canton S* female virgin flies kept in diapause conditions (11 °C, short photoperiod 10 L:14D) for 3 weeks (3wD). Flies were placed under diapause conditions 4-6 h after adult eclosion. Control virgin female flies (1wN) were kept for either 1 week in normal conditions (25 °C, 12 L:12D), or as a sibling control we used flies kept for 3 weeks in normal conditions (3wN). All flies were fed standard *Drosophila* food. Total RNA from whole flies was extracted using Trizol reagent (Invitrogen) according to manufacturer’s protocol and subsequently cleaned with NucleoSpin RNA II kit (Macherey Nagel). 15 flies were used for each biological replicate. Quality and concentration of the RNA were measured with a NanoDrop 2000 spectrophotometer (Thermo Scientific). RNA integrity was analyzed in an Agilent 2100 Bioanalyzer. We included only samples with an intact RNA profile. The same RNA isolation procedure was also used for preparation of samples for quantitative real-time PCR (qPCR).

### Expression profiling

The Affymetrix GeneChip® Drosophila Genome 2.0 Array System was used for microarray analysis following the standard protocol [100 ng RNA was amplified with GeneChip 3 ‘ IVT Express Kit (Affymetrix) and 10 μg of labeled cRNA was hybridized to the chip according to the manufacturer’s instructions].

### Statistical analysis of array data

Analysis was performed in four replicates (except for 1wN controls where 3 replicates were used). Data were preprocessed in Partek Genomic Suit (Partek). The transcription profiles were background corrected using the GCRMA method, quantile normalized and variance stabilized using base-2 logarithmic transformation. Analysis of variance yielded transcripts differentially expressed between analyzed samples (within LIMMA [161]); Storey’s q values [162] were used to select significantly differentially transcribed genes, q < 0.05. The transcription data are MIAME compliant and deposited in the ArrayExpress database (accession E-MTAB-3546).

Statistical analyses were performed in R (http://www.Rproject.org) and within Bioconductor [163] with additional database searches in AmiGO 2 [164]. Functional classification of Gene Ontology (GO) terms [165] was performed in PANTHER [166] with default settings. Differentially expressed genes were selected for gene set enrichment analysis (GSEA). We performed GSEA on genes that mapped to KEGG pathways [167] using the Fisher test and the approach of Tian et al. [168]. For GSEA genes with q < 0.05 and |log FC| ≥ 0.4 were considered differentially expressed. To identify significantly perturbed pathways, we performed SPIA analysis [34] on KEGG pathways.

### Quantitative Real-Time PCR analysis

Quantitative Real-Time PCR (qPCR) experiments were performed on aliquots of RNA extractions performed for microarray tests (see above) as controls of gene expression. Life Technologies SuperScript III Reverse Transcriptase (Life Technologies) was used for cDNA synthesis. Primers were designed using Primer-BLAST (http://www.ncbi.nlm.nih.gov/tools/primer-blast/), except for two cases, where previously designed primers were used: for Actin 88 F [20], and for primers targeting all *timeless* isoforms except N and O [169]. Primers are shown in Additional file 15: Table S5. Levels of mRNA were estimated using SensiFAST SYBR Hi-ROX (Bioline Reagents, London, UK) on a StepOnePlus Real-Time PCR System (Applied Biosystems/Thermo Fisher Scientific). Samples were compared using the ΔΔCt method, with Actin 88 F as reference gene, using the StepOne software v 2.3 (Applied Biosystems). For measurement of transcripts of Toll response genes we followed the protocol for immune genes described in our previous study [20]. We used TaqMan Gene Expression Assays (Applied Biosystems) for following genes: *Drosomycin* (Dm01822006_s1), *Drosomycin-like 5* (Dm02332286_g1) and internal control gene *RpL32* (Dm02151827_g1). Statistical analysis of data was performed in Prism 6 (Graphpad Software Inc, San Diego, CA).

### Microscopy and 10xStat92E-GFP intensity measurements

A Leica MZ FLIII fluorescence stereomicroscope associated with a Panasonic DMC-G2 camera was used to visualize whole flies anesthetized with CO_2_. Confocal images of ovaries were produced in a Zeiss LSM 780 microscope. The following dyes were used: DAPI (1:1000 dilution, Sigma-Aldrich), and Phalloidin-TRITC (1:1000 dilution, Sigma-Aldrich). The samples were mounted in Fluoromount-G (SouthernBiotech). To retrieve the hemocytes, flies were anesthetized, preinjected with small amount of Schneider’s medium (Sigma-Aldrich) containing the anti-coagulant phenylthiourea (PTU) and incubated on ice for 5 min. Thereafter cells were collected on a glass slide with 25 μl of Schneider’s medium with PTU and incubated in a wet chamber for 30 min to allow them to adhere. For fixation 4 % paraformaldehyde in PBS was used for 10 min. After staining with DAPI (1:1000 dilution, Sigma-Aldrich), samples were mounted in Fluoromount-G (SouthernBiotech) and observed under a Zeiss Axioplan 2 microscope coupled to a Hamamatsu ORCA-ER camera (C4742-95). The ImageJ program was used to measure the intensity of GFP. Fluorescence values were recalculated to Corrected total fluorescence (CTF) according to [170].

### Availability of supporting data

Gene microarray data have been deposited at ArrayExpress, accession number E-MTAB-3546.

## Additional files

Additional file 1: Figure S1. Heat map of the top 100 most variable genes in our microarray study. Columns depicted: 3wD (1_3wD-4_3wD) = samples from flies kept for 3 weeks in diapause conditions (11 °C, short photoperiod 10 L:14D); 1wN (1_1wN-3_1wN) = control flies kept in normal conditions (25 °C, 12 L:12D) for 1 week, and 3wN (1-3wN-4_3wN) = sibling controls, 3 week old flies kept in normal conditions (25 °C, 12 L:12D). Each column represents an independent sample. The color key represents the level of regulation (red is up- and blue downregulation). Dark intensities indicate the most up- and down- regulated genes, respectively. The largest subset of most variable genes is upregulated in diapause (3wD) samples and downregulated in 1wN and 3wN controls. However there is also a smaller number of genes, which are downregulated in diapause, clearly upregulated in 3wN control, but less upregulated in 1wN controls. (TIF 1817 kb) Additional file 2: Figure S2. Principal component analysis (PCA) plot. The Y-axis represent the biggest variability in our samples. Our samples fall into two groups. One represents all our samples from diapause conditions (3wD, above line), the second all our control samples (1wN and 3wN under line). Whereas the PCA clearly grouped all 3wD samples (orange subset), it failed to distinguish between 1wN control samples (green subset) and 3wN sibling control (blue subset). (TIF 553 kb) Additional file 3: Dataset S1. Gene list of significantly regulated probes and reciprocal comparison. Transcripts that are significantly altered more than two-fold (|log_2_FC| ≥ 1, q ≤ 0.05) are shown. 3wDvs1wN represent transcripts regulated upon 3 weeks of diapause compared to 1 week control flies, 3wDvs3wN represent genes regulated in diapause samples with comparison to 3 week old sibling controls, 3wNvs1wN represent genes influenced by aging in 3 week old control flies compared to 1 week old control flies. Significantly inhibited transcripts are highlighted in blue, significantly activated in orange. Links to Flybase, Wikigene, Genecard, NCBI, ENSEMBL and ExPASy databases are included. (XLSX 2131 kb) Additional file 4: Table S1. GSEA of KEGG pathway analysis. **(A)** The set of KEGG pathways enriched for upregulated genes encompass mostly carbohydrate metabolic pathways, drug metabolism and circadian rhythm pathway. **(B)** The set of KEGG pathways enriched for downregulated genes covers mostly RNA and DNA metabolism and protein degradation. Genes with |log_2_FC| ≥ 0.04 and q ≤ 0.05 were considered significantly regulated. Pathways highlighted in *italics* were defined only on the basis of experiments performed in mammalian systems. (PDF 45 kb) Additional file 5: Table S2. SPIA of KEGG pathways. Significantly inhibited pathways are highlighted in blue and significantly activated ones in orange. Genes with |log_2_FC| ≥ 0.1 and q ≤ 0.05 were considered significantly regulated. Pathways highlighted in *italics* were defined only on the basis of experiments performed in mammalian systems. (PDF 42 kb) Additional file 6: Table S3. Detailed data on genes in seven signal pathways. Description of genes shown in Figs. 4, 5, 7, 9 and Additional file 8: Figure S4 and discussed in the corresponding sections of the paper. References are also given for these genes. Note that the genes are listed as they are shown in the figures (from top to bottom in the pathways). (PDF 328 kb) Additional file 7: Figure S3. Read-outs from AKH-IIS-TOR pathways. Summary of likely effects of altered signaling in these pathways, based on transcript changes in read-out genes. Based on Fig. 4. (TIF 3492 kb) Additional file 8: Figure S4. Altered gene expression in the MAPK signaling pathway during diapause. This scheme displays a generalized assembly (regardless tissue specificity) of relevant genes in the MAPK signal pathway. Transcript levels (logarithmic fold change, LogFC) are given in red for upregulated, blue for downregulated and black for no significant change (ns; LogFC close to 0). The acronyms are listed in Additional file 6: Table S3 where also references and details of gene/protein functions are given. Decreased expression of all MAP kinases (*Ras85D, phl, Dsor1, rl*) and most positive regulators support a general downregulation of the whole MAPK signaling cascade in diapausing flies. Only one read-out gene was downregulated (dm, *diminutive* a *Drosophila Myc*). (TIF 1437 kb) Additional file 9: Table S4. Genes affected by 3 weeks diapause compared to ones implicated in aging and increased longevity. Microarray study of aging response genes [136], longevity mapping study [33], transcriptome changes in mated and non-mated aging females [7] and candidate study for lifespan genes [135]. Significantly inhibited transcripts are highlighted in blue, significantly activated in orange. (XLSX 77 kb) Additional file 10: Figure S5. Results of overrepresentation test of GO biological process terms for comparisons between our genome-wide transcription study of *D. melanogaster* dormancy and aging studies. 3wD, three weeks diapause (whole animals) our study; Aging, aging response genes [136] and candidate lifespan extending genes [135]. Comparison based on genes in the Venn diagram in Fig. 10a. Term enrichment analysis of Groups A – D are shown separately. (PDF 405 kb) Additional file 11: Figure S6. Term enrichment analysis of GO biological process terms for comparisons among four genome-wide transcription studies of *D. melanogaster*. 3wD, three weeks diapause (whole animals) our study; 1wD, abdomens sampled after one week diapause [140]; cold intolerance [141] and starvation [143]. Comparison based on genes in the Venn diagram in Fig. 10b. Term analysis of Groups A – H are shown separately. (PDF 1267 kb) Additional file 12: Dataset S2. List of genes shared between diapause and response to restricted diet in *D. melanogaster*. We compared our study of three weeks diapause (3wD) to that of an analysis of effects of restricted diet (RF) [145]. Significantly inhibited transcripts are highlighted in blue, significantly activated in orange (for the RF study, genes with q ≤ 0.05 were considered significantly regulated, as was used in original study). Links to Flybase and Genecard databases are included. The Venn diagram summarizes the numbers of significantly regulated genes and how they overlap. (XLSX 173 kb) Additional file 13: Dataset S3. Gene set enrichment analysis (GSEA) of GO terms comparing diapause and response to restricted diet in *D. melanogaster*. We compare our study of three weeks diapause (3wD) to that of an analysis of effects of restricted diet (RF) [145]. **(A)** GSEA for upregulated transcripts identified mostly terms connected to cell cycle and gene expression. **(B)** Transcripts mostly from carbohydrate metabolism are regulated in opposite way in 3wD and RF. **(C)** For downregulated transcripts no significant enriched GO terms (ns) were identified. (XLSX 11 kb) Additional file 14: Dataset S4. Comparisons of diapause response genes from our study of *D. melanogaster* with other transcription studies on reproductive diapause. We compared our study to a *D. montana* candidate gene microarray study [149] and *Culex pipiens* diapause response genes [152]. Significantly inhibited transcripts are highlighted in blue, significantly activated in orange. Links to Flybase are included at genes shared with *D. melanogaster*. (XLSX 15 kb) Additional file 15: Table S5. Primers used for quantitative real-time PCR. (PDF 55 kb)
