# Supplementary figures and images for: Slowed aging during reproductive dormancy is reflected in genome-wide transcriptome changes in Drosophila melanogaster
Source: BMC Genomics. 2016 Jan 13;17:50. doi: 10.1186/s12864-016-2383-1 (PMC4711038; doi:10.1186/s12864-016-2383-1)

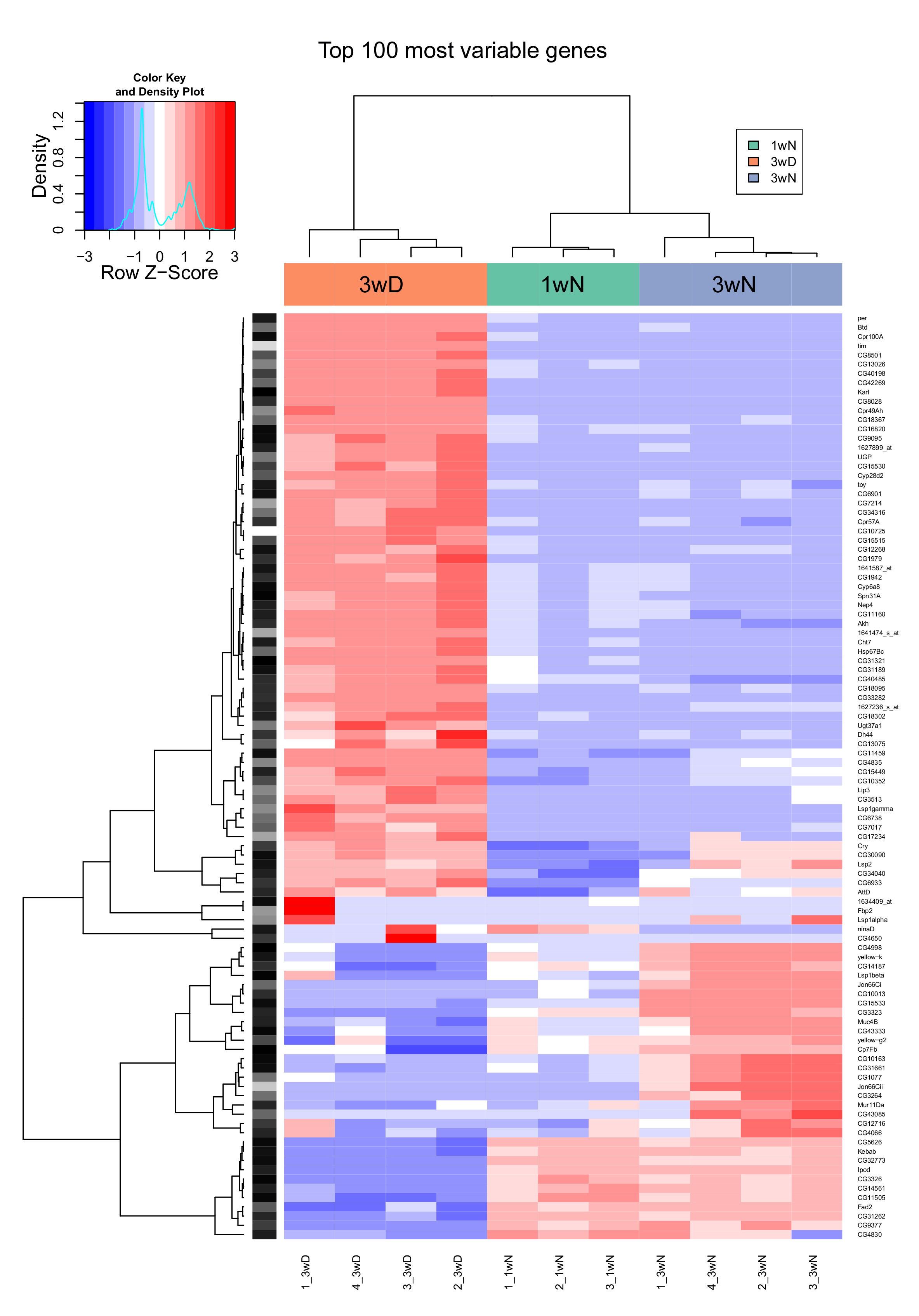

Supplement: Additional file 1: Figure S1. — Heat map of the top 100 most variable genes in our microarray study. Columns depicted: 3wD (1_3wD-4_3wD) = samples from flies kept for 3 weeks in diapause conditions (11 °C, short photoperiod 10 L:14D); 1wN (1_1wN-3_1wN) = control flies kept in normal conditions (25 °C, 12 L:12D) for 1 week, and 3wN (1-3wN-4_3wN) = sibling controls, 3 week old flies kept in normal conditions (25 °C, 12 L:12D). Each column represents an independent sample. The color key represents the level of regulation (red is up- and blue downregulation). Dark intensities indicate the most up- and down- regulated genes, respectively. The largest subset of most variable genes is upregulated in diapause (3wD) samples and downregulated in 1wN and 3wN controls. However there is also a smaller number of genes, which are downregulated in diapause, clearly upregulated in 3wN control, but less upregulated in 1wN controls. (TIF 1817 kb) [file 12864_2016_2383_MOESM1_ESM.tif]

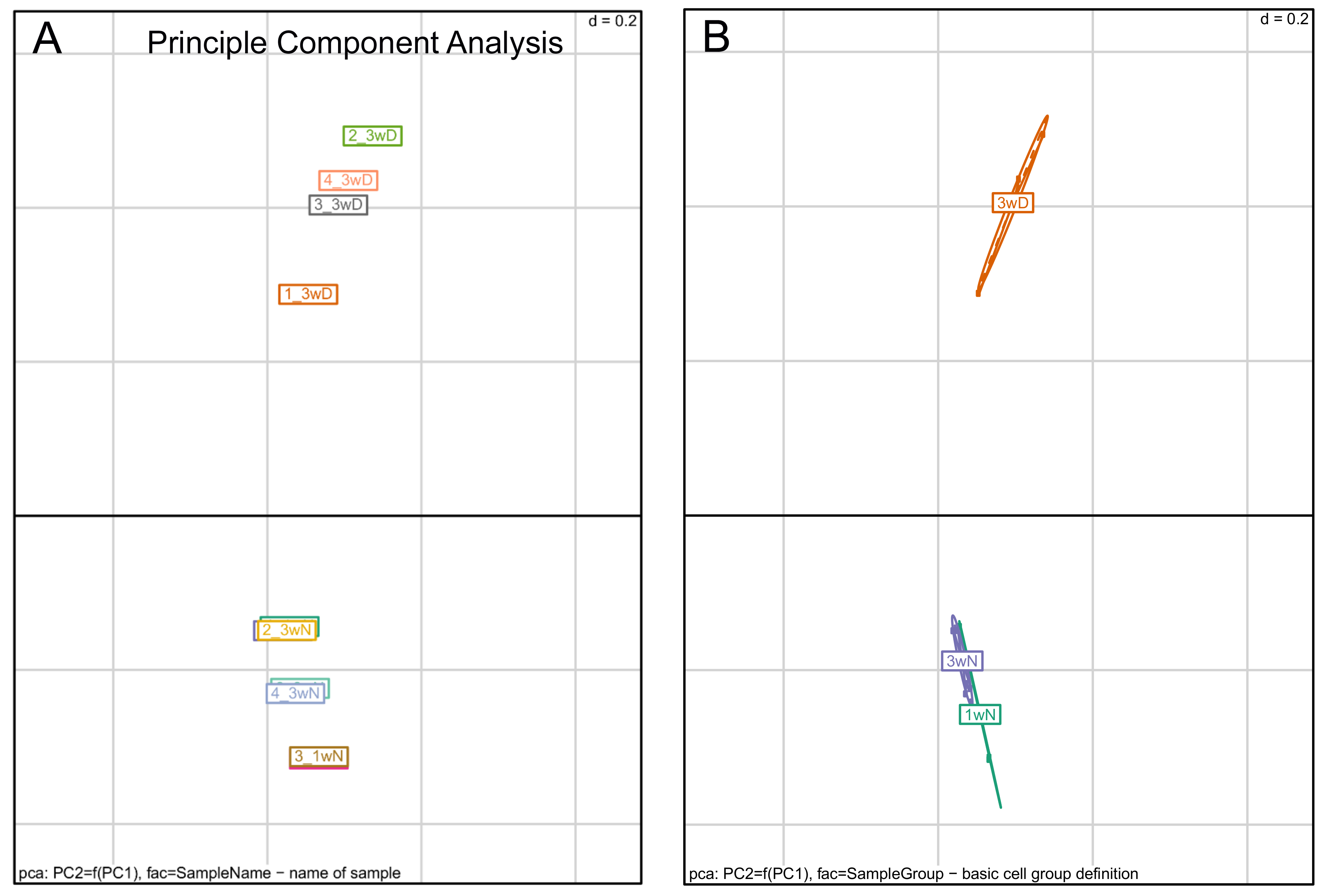

Supplement: Additional file 2: Figure S2. — Principal component analysis (PCA) plot. The Y-axis represent the biggest variability in our samples. Our samples fall into two groups. One represents all our samples from diapause conditions (3wD, above line), the second all our control samples (1wN and 3wN under line). Whereas the PCA clearly grouped all 3wD samples (orange subset), it failed to distinguish between 1wN control samples (green subset) and 3wN sibling control (blue subset). (TIF 553 kb) [file 12864_2016_2383_MOESM2_ESM.tif]

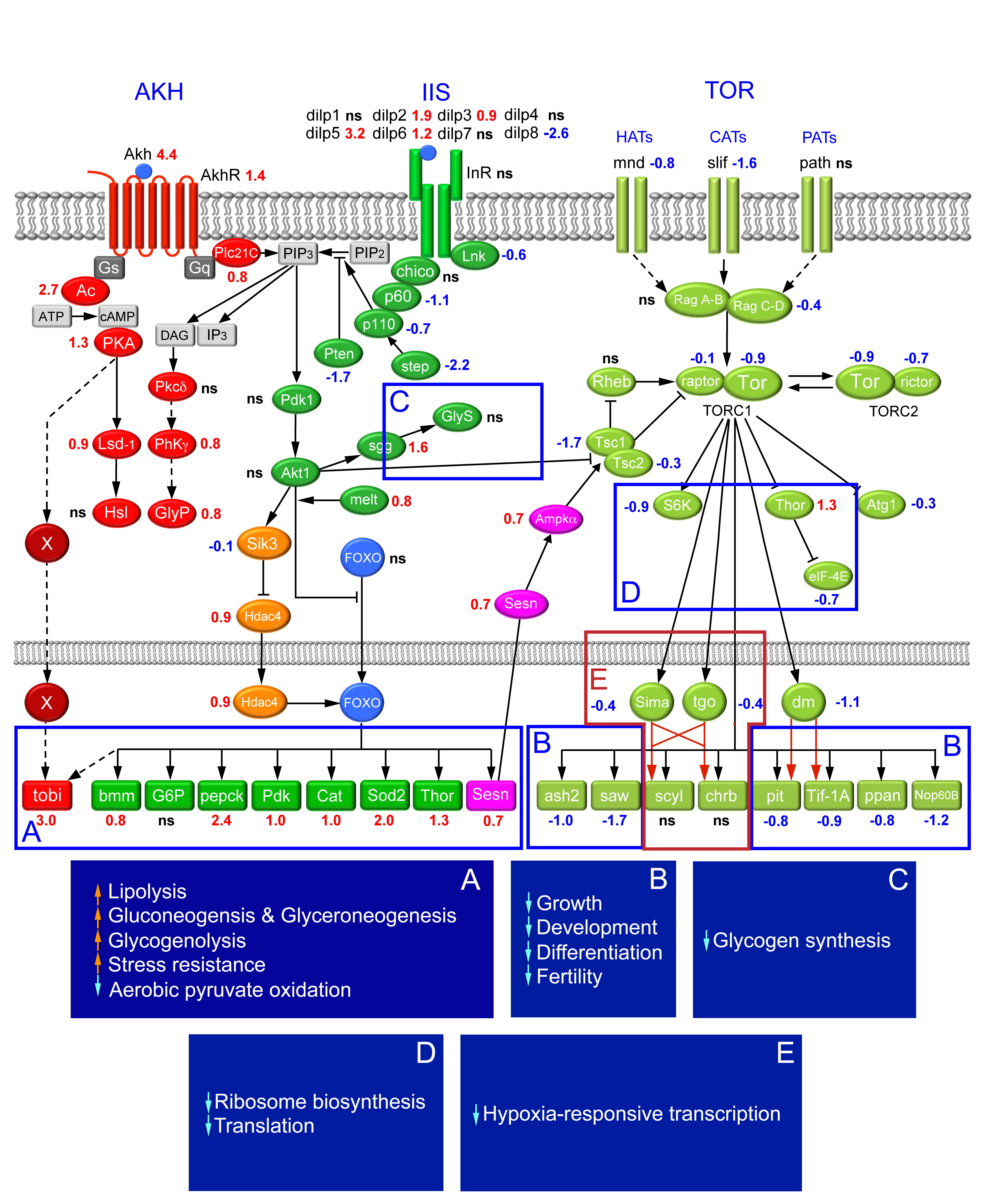

Supplement: Additional file 7: Figure S3. — Read-outs from AKH-IIS-TOR pathways. Summary of likely effects of altered signaling in these pathways, based on transcript changes in read-out genes. Based on Fig. 4. (TIF 3492 kb) [file 12864_2016_2383_MOESM7_ESM.tif]

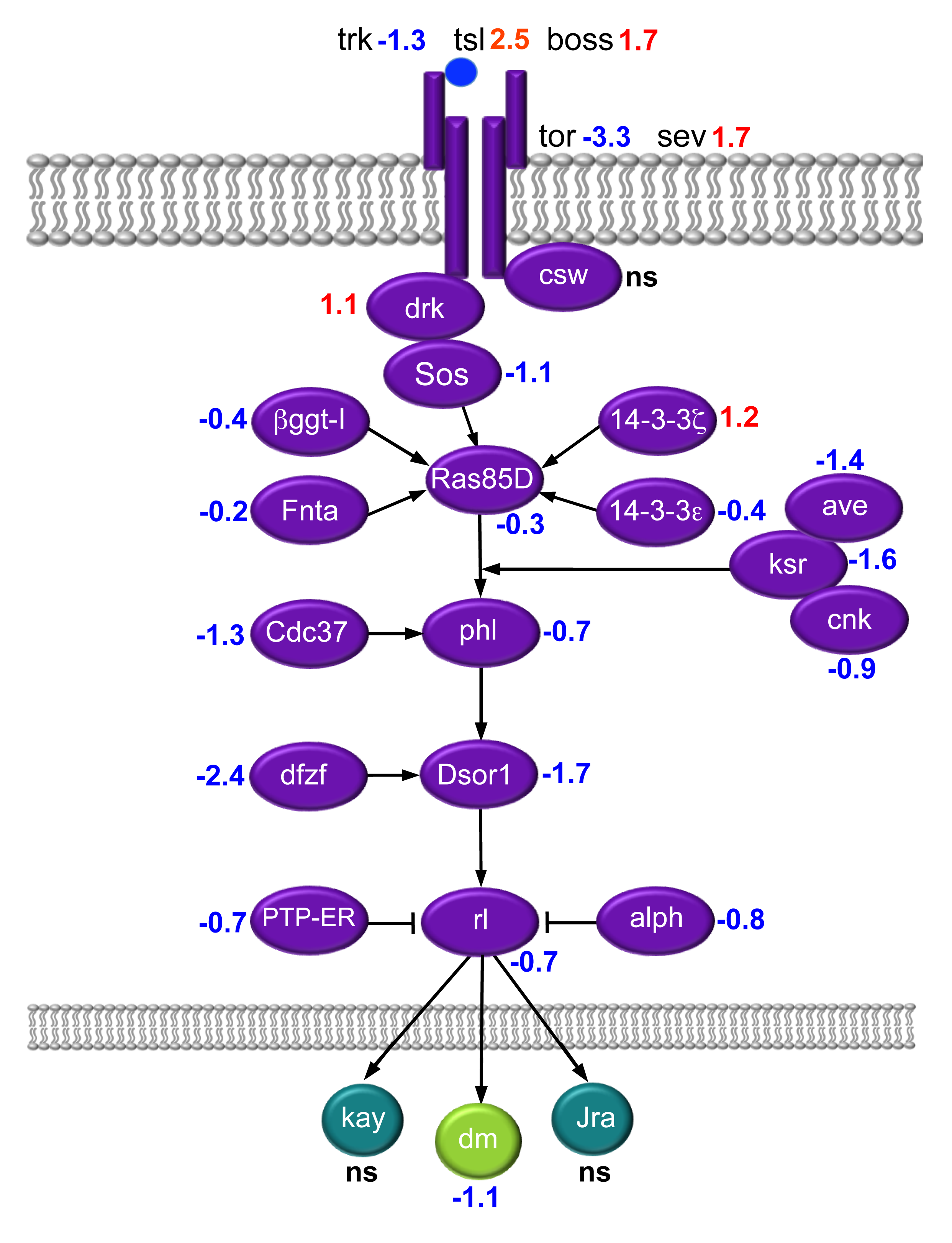

Supplement: Additional file 8: Figure S4. — Altered gene expression in the MAPK signaling pathway during diapause. This scheme displays a generalized assembly (regardless tissue specificity) of relevant genes in the MAPK signal pathway. Transcript levels (logarithmic fold change, LogFC) are given in red for upregulated, blue for downregulated and black for no significant change (ns; LogFC close to 0). The acronyms are listed in Additional file 6: Table S3 where also references and details of gene/protein functions are given. Decreased expression of all MAP kinases (Ras85D, phl, Dsor1, rl) and most positive regulators support a general downregulation of the whole MAPK signaling cascade in diapausing flies. Only one read-out gene was downregulated (dm, diminutive a Drosophila Myc). (TIF 1437 kb) [file 12864_2016_2383_MOESM8_ESM.tif]
