# Supplementary material for: Slowed aging during reproductive dormancy is reflected in genome-wide transcriptome changes in Drosophila melanogaster
Source: BMC Genomics. 2016 Jan 13;17:50. doi: 10.1186/s12864-016-2383-1 (PMC4711038; doi:10.1186/s12864-016-2383-1)
Supplement: Additional file 4: Table S1. — GSEA of KEGG pathway analysis. (A) The set of KEGG pathways enriched for upregulated genes encompass mostly carbohydrate metabolic pathways, drug metabolism and circadian rhythm pathway. (B) The set of KEGG pathways enriched for downregulated genes covers mostly RNA and DNA metabolism and protein degradation. Genes with |log2FC| ≥ 0.04 and q ≤ 0.05 were considered significantly regulated. Pathways highlighted in italics were defined only on the basis of experiments performed in mammalian systems. (PDF 45 kb) [file 12864_2016_2383_MOESM4_ESM.pdf]

A - GSEA of KEGG (Significant enrichment for mostly upregulated genes)

KEGG ID	Name	gDET	gALL	gSIG.Q	q (hyperG_Up)
dme01100	Metabolic pathways	635	940	551	1.41E-20
dme00500	Starch and sucrose metabolism	39	57	37	6.06E-10
dme00982	Drug metabolism - cytochrome P450	44	59	38	1.72E-08
dme00980	Metabolism of xenobiotics by cytochrome P450	43	58	37	3.42E-08
dme00052	Galactose metabolism	22	29	21	2.10E-06
dme00903	Limonene and pinene degradation	46	68	41	4.58E-06
dme00983	Drug metabolism - other enzymes	41	57	34	4.51E-05
dme00040	Pentose and glucuronate interconversions	28	40	25	6.90E-05
dme00830	Retinol metabolism	19	29	18	0.000259
dme00260	Glycine, serine and threonine metabolism	21	27	18	0.000306
dme00053	Ascorbate and aldarate metabolism	18	29	16	0.000501
dme00480	Glutathione metabolism	42	60	35	0.000501
dme00600	Sphingolipid metabolism	20	25	18	0.00157
dme00520	Amino sugar and nucleotide sugar metabolism	32	46	26	0.00241
dme04146	Peroxisome	51	63	41	0.00627
dme00360	Phenylalanine metabolism	9	10	9	0.00837
dme04512	ECM-receptor interaction	9	11	7	0.00837
dme00561	Glycerolipid metabolism	29	44	26	0.00929
dme04142	Lysosome	55	87	47	0.0102
dme00250	Alanine, aspartate and glutamate metabolism	23	29	20	0.0189
dme00604	Glycosphingolipid biosynthesis - ganglio series	6	6	6	0.024
dme00350	Tyrosine metabolism	14	19	14	0.0256
dme00565	Ether lipid metabolism	18	23	16	0.0288
dme01040	Biosynthesis of unsaturated fatty acids	15	16	14	0.0327
dme00010	Glycolysis / Gluconeogenesis	27	49	26	0.033
dme00564	Glycerophospholipid metabolism	41	56	15	0.0383
dme00030	Pentose phosphate pathway	16	22	35	0.0383
dme00514	Other types of O-glycan biosynthesis	21	34	19	0.042
dme00340	Histidine metabolism	7	7	6	0.0468
dme04711	Circadian rhythm - fly	6	7	6	0.0468

B - GSEA of KEGG (Significant enrichment for mostly downregulated genes)

KEGG ID	Name	gDET	gALL	gSIG.Q	q (hyperG_Down)
dme03040	Spliceosome	90	116	86	1.94E-14
dme03013	RNA transport	94	125	91	7.47E-14
dme03018	RNA degradation	45	51	41	1.60E-08
dme03030	DNA replication	29	34	29	1.88E-07
dme03010	Ribosome	42	92	41	6.99E-07
dme03020	RNA polymerase	22	27	50	6.99E-07
dme03008	Ribosome biogenesis in eukaryotes	53	84	22	6.99E-07
dme03420	Nucleotide excision repair	27	37	27	3.15E-06
dme00970	Aminoacyl-tRNA biosynthesis	37	46	33	6.01E-06
dme00240	Pyrimidine metabolism	64	80	59	0.000826
dme03430	Mismatch repair	15	19	15	0.00137
dme03022	Basal transcription factors	25	30	24	0.00173
dme04914	*Progesterone-mediated oocyte maturation*	35	44	33	0.0086
dme03440	Homologous recombination	14	18	13	0.0178
dme03050	Proteasome	33	50	32	0.0278
dme03015	mRNA surveillance pathway	46	59	63	0.0348
dme04120	Ubiquitin mediated proteolysis	72	98	40	0.0348
dme00563	Glycosylphosphatidylinositol(GPI)-anchor biosynthesis	15	20	14	0.0355

gDET	genes detected on array
gALL	genes defined in pathway
gSIG_Q	significantly expressed genes in our study, |log FC| > 0.4, q < 0.05
