# Supplementary material for: Slowed aging during reproductive dormancy is reflected in genome-wide transcriptome changes in Drosophila melanogaster
Source: BMC Genomics. 2016 Jan 13;17:50. doi: 10.1186/s12864-016-2383-1 (PMC4711038; doi:10.1186/s12864-016-2383-1)
Supplement: Additional file 5: Table S2. — SPIA of KEGG pathways. Significantly inhibited pathways are highlighted in blue and significantly activated ones in orange. Genes with |log2FC| ≥ 0.1 and q ≤ 0.05 were considered significantly regulated. Pathways highlighted in italics were defined only on the basis of experiments performed in mammalian systems. (PDF 42 kb) [file 12864_2016_2383_MOESM5_ESM.pdf]

KEGG ID	TITLE	gDET	gALL	gSIG_Q	pG_Q	Status_Q	Description
**dme04914**	*Progesterone-mediated oocyte maturation*	41	41	25	0.00236	Inhibited	Covers part of insulin signaling and MAPK signaling pathway.
**dme03460**	*Fanconi anemia pathway*	26	NA	17	0.00267	Activated	The Fanconi anemia pathway is required for the efficient repair of damaged DNA, especially interstrand cross-links (ICLs).
**dme04512**	**ECM-receptor interaction**	10	10	7	0.0269	Activated	ECM (Extracellular matrix) receptor interaction
**dme04711**	**Circadian rhythm - fly**	8	10	6	0.0271	Inhibited	

gDET genes detected on array
gALL genes defined in pathway
gSIG_Q significantly expressed genes in our study, |log FC| > 0.1, q < 0.05

NA not allowed
