# Supplementary material for: Slowed aging during reproductive dormancy is reflected in genome-wide transcriptome changes in Drosophila melanogaster
Source: BMC Genomics. 2016 Jan 13;17:50. doi: 10.1186/s12864-016-2383-1 (PMC4711038; doi:10.1186/s12864-016-2383-1)
Supplement: Additional file 6: Table S3. — Detailed data on genes in seven signal pathways. Description of genes shown in Figs. 4, 5, 7, 9 and Additional file 8: Figure S4 and discussed in the corresponding sections of the paper. References are also given for these genes. Note that the genes are listed as they are shown in the figures (from top to bottom in the pathways). (PDF 328 kb) [file 12864_2016_2383_MOESM6_ESM.pdf]

Supplementary Material Tables 1 – 7 Gene acronyms, full names and functions in signal pathways (to accompany Figures 4, 5, 7, 9 and S8 Fig.).

Table 1 Insulin/IGF signaling (IIS) pathway components and pathway regulators in *D. melanogaster*

Acronym	Gene name	CG number	Function/description of gene product	Reference
dilp2	*Drosophila insulin-like peptide 2*	CG8167	*dilp2* is expressed in a set of median neurosecretory cells (mNSC), known as insulin-producing cells (IPCs); regulates growth, metabolism, stress and aging	[1-3]
dilp1	*Drosophila insulin-like peptide 1*	CG14173	*dilp1* transcripts are detected in larval-pupal IPCs; expression and function in adult are unknown	[4,5]
dilp4	*Drosophila insulin-like peptide 1*	CG6736	*dilp4* expression is not detected in adult flies; functions are unknown	[1,5]
dilp3	*Drosophila insulin-like peptide 3*	CG14167	*dilp3* is co-expressed with *dilp2* and *dilp5* in IPCs and in the muscle cells of the adult midgut	[1,4,6]
dilp5	*Drosophila insulin-like peptide 5*	CG33273	*dilp5* is expressed in IPCs and in follicle cells of the ovary as well as principal cells of the renal tubules	[1,4,7]
dilp6	*Drosophila insulin-like peptide 6*	CG14049	*dilp6* in is expressed in adipose cells of the fat body	[8,9]
dilp7	*Drosophila insulin-like peptide 7*	CG13317	*dilp7* is expressed in about 20 neurons of the abdominal neuromeres; may function as *Drosophila* relaxin	[1,10,11]
dilp8	*Drosophila insulin-like peptide 8*	CG14059	*dilp8* is expressed in the imaginal discs of larvae; in adult *dilp8* expression is abundant in ovaries	[12,13]
InR	*Insulin-like receptor*	CG18402	Tyrosine-protein kinase with insulin-activated receptor activity; receptor of IIS signaling pathway	[14,15]
Chico *(IRS)*	*Chico* *(Insulin receptor substrate)*	CG5686	Insulin receptor substrate	[16]
Lnk	*Lnk*	CG17367	Insulin receptor adaptor protein	[17,18]
Dp110 *(Pi3K92E)*	*Dp110* *(Pi3K92E)*	CG4141	Catalytic subunit of phosphatidylinositol 3-kinase (PI3K), a main upstream kinase in IIS	[19,20]
Dp60 *(Pi3K21B)*	*Dp60* *(Pi3K21B)*	CG2699	Adaptive subunit of phosphatidylinositol 3-kinase (PI3K)	[21]
step	*steppke*	CG11628	PI3K upstream factor Steppke	[22]

Akt1 *(PKB)*	*Akt1* *(Protein kinase B)*	CG4006	AKT, also known as protein kinase B, is a main downstream kinase in IIS, phosphorylates the transcription factor FOXO	[15]
Pdk1	*Phospho-inositide-dependent kinase 1*	CG1210	PDK-1 is a central mediator of the signaling between PI3K and various intracellular serine/ threonine kinases including Akt (PKB)	[23]
melt	*melted*	CG8624	Melted modulates IIS pathway in *Drosophila* interacting with both Tsc1 and FOXO and can recruit these proteins to the cell membrane	[24]
foxo	*forkhead box, sub-group O*	CG3143	FOXO is a key component of the insulin signaling cascade, which is phosphorylated by dAkt, leading to cytoplasmic retention and inhibition of its transcriptional activity	[25]
sgg / GSK3	*shaggy/ Glycogen Synthase Kinase 3*	CG2621	Shaggy is a Drosophila orthologue of glycogen synthase kinase-3; which regulates glycogen synthesis by inhibiting glycogen synthase	[26]
GlyS (GS)	*Glycogen synthase*	CG6904	Glycogen synthase is the main enzyme involved in converting glucose to glycogen	[27]
Pepck	*Phosphoenol-pyruvate carboxykinase*	CG17725	PEPCK is a key enzyme of both gluconeogenesis and glycerogenesis	[28,29]
bmm *(Atgl)*	*Brummer* *(adipocyte triglyceride lipase)*	CG5295	Triacylglyceride lipase Brummer, a homolog of human adipocyte triglyceride lipase (ATGL)	[30]
Pdk	*Pyruvate dehydro-genase kinase*	CG8808	PDK is responsible for an inactivation of pyruvate dehydrogenase complex and decrease of Krebs cycle turnover with a re-direction of pyruvate into anaerobic oxidation	[31]
Cat	*Catalase*	CG6871	Catalase is one of the key antioxidants enzymes; *Cat* expression is under FOXO control	[32,33]
Sod2	*Superoxide dismutase 2*	CG8905	SOD2 is a manganese/iron superoxide dismutase, participating in antioxidant defence; *Sod2* expression is under FOXO control	[32,33]
Thor *(4E-BP)*	*Thor* *(eukaryotic initiation factor 4 binding protein)*	CG8846	*Thor* encodes eukaryotic initiation factor 4 binding protein (4E-BP), an inhibitor of translation and is a FOXO transcriptional target, However, 4E-BP is also known to be under a control of the TOR-signaling pathway	[34,35]

Sik3	Salt-inducible kinase 3	CG42856 (CG15072, CG18604)	SIK3 is a member of the AMPK family of Ser/Thr kinases, which play a critical role in energy balance. Being activated by AKT, SIK3 promotes lipid storage by blocking FOXO activity	[29]
HDAC4	*HDAC4*	CG1770	Gene encodes histone deacetylase class II, which regulates transcriptional activity of certain transcriptional factors, including FOXO	[29,36]
HNF4	*Hepatocyte nuclear factor 4*	CG9310	HNF4 is a nuclear receptor, which in association with the transcription factor FOXO activates expression of gluconeogenic genes	[37-39]

Table 2 TOR signaling pathway components in *D. melanogaster*

Acronym	Gene name	CG number	Function/Description of the gene product	Reference
slif	*slimfast*	CG11128	cationic amino acid transporter (CAT)	[40]
path	*pathetic*	CG3424	proton-assisted amino acid transporter (PAT)	[41]
mnd	*minidiscs*	CG3297	heterodimeric amino acid transporter (HAT)	[42]
raptor	*raptor*	CG4320	A rapamycin-sensitive TOR companion within TORC1 complex	(see [43])
Tor	*Target of rapamycin*	CG5092	TOR kinase, a main component of TORC1 and TORC2 complexes	(see [43])
rictor	*rapamycin-insensitive companion of Tor*	CG8002	A rapamycin-insensitive companion of TOR in TORC2 complex	(see [43])
S6k	*RPS6-p70-protein kinase*	CG10539	Ribosomal protein S6 kinase; a downstream target of TOR	(see [43])
Thor	*Thor*/4E-BP	CG8846	Eukaryotic translation initiation factor 4E binding; a downstream target of TOR	(see [43])
RagA-B	*Ras-related GTP binding A/B*	CG11968	Rag GTPases are activators of TORC1 in response to amino acid signals	[44,45]
RagC-D	*Ras-related GTP binding C/D*	CG8707	Rag GTPases are activators of TORC1 in response to amino acid signals	[44,45]
Tsc1	*Tsc1/tuberous sclerosis complex 1*	CG6147	A part of tuberous sclerosis complex (TSC), consisting of two subunits in *Drosophila* TSC1 and gigas (TSC2)	[46]
gig	*gigas (Tsc2)*	CG6975	A part of tuberous sclerosis complex (TSC) with an ability of TSC2 to act as a GTPase-activating protein to inactivate GTPase Rheb (Ras homologue enriched in brain), an ultimate activator of TOR kinase	[46]
Rheb	*Ras homolog enriched in brain*	CG1081	Small GTPase Rheb is a direct target of Tsc2 GAP activity	[47]

AMPKα (snf1a)	*AMP-activated protein kinase α subunit (snf1a)*	CG3051	AMPK is a low ATP:ADP ratio sensor, which can directly phosphorylate TSC2, enhancing its GAP activity and, as result, leading to TORC1 inhibition	[48-50]
Sesn	*Sestrin*	CG11299	Sestrin transcription in *Drosophila* is under FOXO control and leads to AMPK activation and in turn an enhancement of Tsc1/Tsc2 inhibition of TORC1	[51]
eIF-4E	*Eukaryotic initiation factor 4E*	CG4035	Translation initiation factor that interacts with *Drosophila* eIF4E-binding protein (4E-BP)	[52]
Tif-IA	*Tif-IA*	CG3278 (CG5951)	TIF-IA protein is a Pol I transcription initiation factor	Reviewed [53]
Atg1	*Autophagy-related 1*	CG10967	Atg 1 is a main autophagy gene, whose kinase activity is known to be inhibited by TOR	Reviewed in [54]
sima (HIF-1α)	*similar (Hypoxia inducible factor 1α)*	CG45051 (CG7951)	One of the main hypoxia-inducible transcription factors, which represent *Drosophila* HIF1-a	[55]
tgo (Arnt, HIF-1b)	*tango (Arnt, Hypoxia inducible factor 1b)*	CG11987	One of the main hypoxia-inducible transcription factors, which represent *Drosophila* HIF1-b	[55]
scyl	*scylla*	CG7590	One of the hypoxia induced genes, homologue of mammalian REDD1	[56]
chrb	*charybde*	CG7533	One of the hypoxia induced genes, homologue of mammalian REDD2	[56]
Dm (Myc)	*diminutive (Myc)*	CG10798	Diminutive is a *Drosophila* Myc homologue; a transcriptional factor, whose expression and activity is under FOXO and TORC1 control, respectively, and involved in the transcriptional regulation of ribosome synthesis genes such as Pol I transcription factor *Tif-IA*	[57]
saw/ CG3071	*saw/CG3071*	CG3071	An ortholog of human SAW; is under TOR-dependent transcriptional control and encodes a protein with conserved functional roles in growth (positive growth regulator)	[58]
ash2	absent, small, or homeotic discs 2	CG6677	An ortholog of human ASHL; is under TOR-dependent transcriptional control and encodes a protein, that is a negative growth regulator	[58]
pit	*pitchoune*	CG6375	Pitchoune is a helicase, required for cell growth and proliferation; a potential target of d-Myc	[59]
ppan	*peter pan*	CG5786	Required for mitotic proliferation, growth and some aspects of differentiation	[60]

Nop60B (mnf)	*Nucleolar protein at 60B (minifly)*	CG3333	Gene with pleiotropic effects on viability and fertility	[61]

Table 3 Proposed AKH signalling pathway components in *D. melanogaster,* partly based on experimental findings from other insects

**Acronym**	**Gene name**	**CG number**	**Function/Description of the gene product**	**Reference**
Akh	*Adipokinetic hormone*	CG1171	AKH is produced by corpora cardiaca glandular cells; functional homolog of glucagon and regulates glucose homeostasis	[62,63]
AkhR (GRHR)	*Adipokinetic hormone receptor (Gonadotropin releasing hormone receptor)*	CG11325	AKHR belongs to the G protein-coupled proteins, structurally related to receptors of the vertebrate gonadotropin releasing hormone	[64]
Plc21C	*Phospholipase C at 21C*	CG4574	Activation of PLC21C is triggered by binding of AKH to AKHR, initiating a phospholipase-C/proteinkinase-C signalling cascade, when enhanced phospholipase C cleaves phosphatidylinositol (3,4,5)-trisphosphate (PIP_3) into diacylglycerol (DAG) and inositol (1,4,5) phosphate (IP_3), which, in turn, transduces a signal to protein kinase C	[65-67]
Pkcδ	*Protein kinase C delta*	CG42349 (CG10524)	Protein kinase C is activated by DAG as result of phospholipase C activation	[65,66]
Cam	*Calmodulin*	CG8472	Calmodulin is a calcium binding signal molecule that assists phospholipase-C/proteinkinase-C signalling cascade	[65]
PhKγ	*Phosphorylase kinase gamma*	CG1830	*Drosophila* phosphorylase kinase gamma is a putative homologue of the vertebrate phosphorylase kinase catalytic gamma-subunit, which is a target of phospholipase-C/proteinkinase-C signalling cascade and a direct activator of glycogen phosphorylase	reviewed in [66,68]
GlyP (GP)	*Glycogen phosphorylase*	CG7254	Glycogen breakdown enzyme	[69]
Ac13E	Adenylyl cyclase 35C	CG9210	Activation of adenylyl cyclase leads to elevation of cAMP, which activates protein kinase A (PKA)	Reviewed in [66,67]
Pka/Pka-C1	*Protein kinase A/cAMP-dependent protein kinase 1*	CG4379	PKA with assistance of calcium ions stimulates Plin1 and, in turn, hormone-sensitive lipase (HSL). In *Manduca sexta* binding of AKH by its receptor transduces a signal via cAMP to PKA, which phosphorylates Plin1, but the AKH/AKHR-mediated PKA activation is only	[70,71]

			partly confirmed for *Drosophila*	
Lsd-1 (plin1)	*Lipid storage droplet-1 (Perilipin 1)*	CG10374	Perilipin 1 is a *Drosophila* lipid droplet surface protein that has opposite roles under normal and starvation conditions, preventing lipases from accessing lipid droplets under basal lyposis, but promoting lipase access to lipids under starvation. Thus, under energy deficit Perilipin1 is phosphorylated by PKA in response to hormonal signals. Phosphorylated Perilipin1 facilitates maximal lipolysis by recruiting hormone-sensitive lipase (HSL). Plin1 is found to be downstream effector for AKH signalling. The mechanism is conserved for mammals and *Drosophila*	[70,72-74]
Hsl	Hormone-sensitive lipase	CG11055	Hormone-sensitive lipase is the sole homolog of mammalian HSL in *Drosophila*	[72,75]
tobi	*target of brain insulin*	CG11909	*tobi* expression was postulated to be under AKH signaling control through an unknown X transcription factor; *tobi* encodes alpha-1,4-glucosidase	[76]

Table 4 JAK-STAT signaling pathway components in *D. melanogaster*

**Acronym**	**Gene name**	**CG number**	**Function/Description of the gene product**	**Reference**
dome	*domeless*	CG14226	Encodes cytokine-like receptor Dome, which expression is under Stat92E control	[77-80]
hop	*hopscotch*	CG1594	Encodes *Drosophila* Janus kinase (JAK)	[77-79]
Stat92E	*Signal-transducer and activator of transcription protein at 92E*	CG4257	Transcriptional factor Stat92E	[77-79]
RanBPM	*Ran-binding protein M*	CG42236 (CG11763)	The *Drosophila* homologue of *RanBP10* known to control signal-dependent nuclear translocation of Stat92E	[81]
RanBP3	*Ran binding protein 3*	CG10225	Control of the signal-dependent nuclear translocation of Stat92E	[81]
Socs36E	*Suppressor of cytokine signaling at 36E*	CG15154	The strongest negative effector of JAK/STAT signaling among three Socs. Involved in both lysosomal degradation of Dome and preventing its phosphorylation by hop; participates in a negative feedback loop: *Socs36E* expression is transcriptionally activated by JAK signaling	[82-85]
Socs16D	*Suppressor of cytokine signaling at*	CG8146	Socs16D is a negative regulator of JAK-STAT signalling	[82,83]

	16D			
Socs44A	*Suppressor of cytokine signaling at 44A*	CG2160	Socs44E is a negative regulator of JAK-STAT signalling through hop	[82,83]
Ptp61F	*Protein tyrosine phosphatase 61F*	CG9181	Ptp61F is a transcriptional target of Stat92E, which also functions via a negative feedback and deactivates hop	[78,81]
Su(var)2-10	*Suppressor of variegation 2-10 (zimp, dPIAS)*	CG8068	The *Drosophila* PIAS (protein inhibitors of activated STATs) homolog, responsible for binding and blocking of Stat92E dimers	[86]
os/upd1	*outstretched (unpaired 1)*	CG5993	Receptor Dome ligand	[87]
upd2	*unpaired 2*	CG5988	Receptor Dome ligand	[87]
upd3	*unpaired 3*	CG33542 (CG5963, CG15062)	Receptor Dome ligand	[87]
Cnot4	*Cnot 4 homologue*	CG31716 (CG5251)	Transcription regulation complex subunit, needed for proper Stat92E DNA binding	[88]
BRWD3	*bromo-domain-containing protein*	CG31132 (CG6400)	A positive regulator with an elusive function	[79,89]
ken	*ken and barbie (ok)*	CG5575	Stat92E transcriptional antagonist	[79]
Diedel	*Diedel*	CG11501	A suggested negative regulator of JAK/STAT signalling with unclear molecular mechanism	[89,90]
et	*eye transformer (latran)*	CG14225	A negative regulator of JAK/STAT signaling	[91]
dally	*division abnormally delayed (gem)*	CG4974	A glypican involved in Upd distribution, responsible in activating of JAK/STAT pathway	[92]
dlp	*dally-like protein*	CG32146	An activator of JAK/STAT pathway by participating in Upd distribution	[92]
TotA	*Turandot A*	CG31509	Stress-inducible humoral factor, known to play an important role in stress tolerance and immune response	[87,93]
Nop56	*Nop56*	CG13849	A JAK/STAT target gene with Stat92E binding cites; a growth regulator	Reviewed in [94]
Jheh2	*Juvenile hormone epoxide hydrolase*	CG15102	A JAK/STAT target gene with Stat92E binding cites; JHEH is mainly involved in xenobiotic biotransformation, not just in juvenile hormone metabolism in *D. melanogaster*	Reviewed in [94,95]
ftz-f1	*ftz transcription factor 1*	CG4059	A JAK/STAT target gene with Stat92E binding cites; zinc finger, nuclear hormone receptor-type	Reviewed in [94]
H	*Hairless*	CG5460	A JAK/STAT target gene with Stat92E binding cites; negative regulator of Notch	Reviewed in [94]

			pathway	
Tom	*Twin of m4 (barbu)*	CG5185	A JAK/STAT target gene with Stat92E binding cites; negative regulator of Notch pathway	Reviewed in [94]
Paip2	*polyA-binding protein interacting protein 2*	CG12358	A JAK/STAT target gene with Stat92E binding cites; negative regulator of translation	Reviewed in [94]
Zfh-1	*zinc finger homeodomain 1*	CG1322	A JAK/STAT target gene with Stat92E binding cites; transcription factor that binds E box sequences and acts as an active transcriptional repressor	[96,97]
eve	*even skipped*	CG2328	A JAK/STAT target gene that functions in development	[98]
chinmo	*chronologically inappropriate morphogenesis*	CG31666 (CG17156)	A functional effector of the JAK/STAT pathway that regulates eye development, tumor formation, and stem cell self-renewal in *Drosophila*	[99]
TotC	Turandot C	CG31508	Stress-inducible humoral factor, known to play an important role in stress tolerance and immune response	[93]
TotM	Turandot M	CG14027	Stress-inducible humoral factor, known to play an important role in stress tolerance and immune response	[93]
TotX	Turandot X	CG31193	Stress-inducible humoral factor, involved in response to heat and oxidative stress as well as bacterial infection	[93]
vir-1	*virus-induced RNA-1*	CG31764	A JAK/STAT readout gene, known to be required for antiviral protection	[100]
Tep2	*Thioester-contaning protein 2*	CG7052 (CG18589)	Resembles the vertebrate complement factors and α-macroglobulin family of protease inhibitors, and is known to be expressed mainly in hemocytes under JAK/STAT signalling control	[77,101]
Tep3	*Thioester-contaning protein 3*	CG7068	Resembles the vertebrate complement factors and α-macroglobulin family of protease inhibitors, and is known to be expressed mainly in hemocytes under JAK/STAT signalling control	[77,101]
Tep4	*Thioester-contaning protein 4*	CG10363	Resembles the vertebrate complement factors and α-macroglobulin family of protease inhibitors, and is known to be expressed mainly in hemocytes under JAK/STAT signalling control	[77,101]

Table 5 Toll signaling pathway in *D. melanogaster*

Acronym	Gene name	CG number	Function/Description of the gene product	Reference
PGRP-SA	*Peptidoglycan recognition protein SA*	CG11709	Pattern recognition receptor for Gram-positive bacteria, cooperates with GNBP1	[74]
PGRP-SD	*Peptidoglycan recognition protein SD*	CG7496	Pattern recognition receptor for Gram-positive bacteria	[102]
GNBP1	*Gram-negative bacteria binding protein 1*	CG6895	Pattern recognition receptor for Gram-positive bacteria, cooperates with PGRP-SA	[74]
GNBP3	*Gram-negative bacteria binding protein 3*	CG5008	Pattern recognition receptor for fungal cell wall components	[95]
modSP	*modular serine protease*	CG31217	Protease from protease cascade leading to processing spatzle	[84]
grass	*Gram-positive Specific Serine protease*	CG5896	Protease from activation cascade leading to processing spatzle	[103]
spheroide	*spheroide*	CG9675	Protease from activation cascade leading to processing spatzle	[69]
spirit	*spirit*	CG2056	Protease from activation cascade leading to processing spatzle	[69]
sphinx1/2	*sphinx1/ sphinx2*	CG32383/ CG32382	Proteases from activation cascade leading to processing spatzle	[69]
nec	*necrotic*	CG1857	Serine proteinase inhibitor, inhibitor of persephone	[104]
psh	*persephone*	CG6367	Serine protease activated by several virulence factors	[105]
SPE	*Spätzle-Processing Enzyme*	CG16705	Serine protease responsible for spätzle cleavage	[106]
spz	*spätzle*	CG6134	Toll receptor ligand	[105]
Tl	*Toll*	CG5490	Toll receptor	[105]
mop	*myopic*	CG9311	Endocytosis complex component	[107]
Myd88	*Myd88*	CG2078	Adaptor protein of Toll receptor, part of heterotrimeric DD complex	[105]
tub	*tube*	CG10520	Adaptor protein of Toll receptor, part of heterotrimeric DD complex	[105]
pll	*pelle*	CG5974	Adaptor protein of Toll receptor, part of heterotrimeric DD complex	[105]
pli	*Pellino*	CG5212	Pelle/IRAK interacting protein	[108]
Gprk2	*G protein-coupled receptor kinase 2*	CG17998	Cactus interacting protein	[109]
cact	*cactus*	CG5848	IκB factor	[59]
Dif	*Dorsal-related*	CG6794	NF-κB transcription factor	[110]

	immunity factor			
dl	*dorsal*	CG6667	NF-κB transcription factor	[105]
Deaf1	*Deformed epidermal autoregulatory factor-1*	CG8567	Transcription factor	[111]
Drs	*Drosomycin*	CG10810	Antimicrobial protein (AMP)	[112]
Drsl5	*Drosomycin-like 5*	CG10812	AMP-like protein	[113]
IM1	*Immune induced molecule 1*	CG18108		[113]
IM2	*Immune induced molecule 2*	CG18106		[113]

Table 6 Torso/MAPK signaling pathway components in *D. melanogaster*

Acronym	Gene name	CG number	Function/Description of the gene product	Reference
tsl	*torso-like*	CG6705	Torso-like is a *Drosophila* membrane attack complex/perforin-like protein. It binds the receptor tyrosine kinase Torso (Tor) and activates the Torso signaling cascade during terminal pattering of *Drosophila* embryo. A role of Tsl in control of body size and development, independent on Torso signaling.	[103,114,115]
tor	*torso*	CG1389	The Torso receptor tyrosine kinase (RTK) is distributed throughout the membrane in the embryo and activated by its ligand, like torso-like. Upon activation, two neighboring Torso receptors dimerize and transphosphorylate each other, recruiting corkscrew and the adaptor molecule Drk.	[116]
trk	*trunk*	CG5619	Trunk (expressed in the embryo) is a putative ligand of Torso receptor, responsible for the spatially restricted Tor activation. Trunk must be cleaved under control of Torso-like (Tsl) secretion at embryo pole in order to bind Tor. Torso-like independent cleavage of Trunk has also been found.	[117]
boss	bride of sevenless	CG8285	Boss is a G protein-coupled receptor (GPCR) that was first identified as a ligand for the Sevenless tyrosine kinase, which is involved in eye differentiation in *Drosophila*. Recently a role of Boss in glucose sensing and regulation of sugar and lipid metabolism was shown.	[118,119]
sev	*bride of sevenless*	CG18085	Sevenless is a receptor tyrosine kinase (RTK), initiating the signaling pathway responsible for	[120,121]

			differentiation of ommatidial cells during development.	
drk	*downstream of receptor kinase*	CG6033	Drk is an adaptor molecule that couples receptor tyrosine kinase (RTK) to son-of-sevenless (Sos). The association of Drk, Sos and RTK allows Sos to interact with membrane-bound Ras1.	[122]
csw	*corkscrew*	CG3954	Corkscrew is a non-receptor protein tyrosine phosphatase, homologous to the mammalian SHP-2 protein.	[123]
Sos	*Son of sevenless*	CG7793	Sos encodes a guanine-nucleotide exchange factor, which exchanges inactive GDP-Ras I for active GTP-Ras I.	[124]
βggt-I	*β subunit of type I geranylgeranyl transferase*	CG3469	A factor involved in RAS posttranslational modification (prenylation); a positive regulator of MAPK signaling.	[125,126]
Fnta	*Farnesyl transferase α*	CG2976	FNTA is *Drosophila* ortholog of the mammalian alpha farnesyltransferase subunit, an enzyme responsible for RAS farnesylation, which is required for Ras membrane localization and cell transforming activity.	[102,126]
Hmgcr	*Hydroxymethyl-glutaryl-(HMG) Coenzyme A reductase*	CG10367	HMGCR functions is required for farnesylation of RAS membrane-associated proteins.	[126]
Ras85D (Ras1)	*Ras oncogene at 85D (Ras 1)*	CG9375	Ras I associates with Raf1 serine/threonine kinase, recruiting it to the plasma membrane.	[127]
phl (Raf1)	*pole hole (Raf1)*	CG2845	Pole hole, a *Drosophila* Raf1 that may function as the first molecule in the MAPK cascade, phosphorylating and activating Dsor 1 (*Drosophila* MEK).	[109,128]
Dsor1 (MEK)	*Downstream of raf1*	CG15793	Dsor (MEK) is MAPKK, which is phosphorylated by Raf1 (MAPKKK), in turn, phosphorylates and activates rolled/ERK (MAPK).	[111]
rl (ERK, MAPK)	rolled *(Mitogen-activated protein kinase)*	CG12559	Rolled encodes a *Drosophila* MAPK – a central kinase in the MAPK signalling cascade, which phosphorylates a number of transcriptional factors and cytoplasmic targets.	[129]
ksr	*kinase suppressor of ras*	CG2899	*ksr* encodes a protein kinase, which is a general and evolutionarily conserved component of the RAS signaling pathway that acts between RAS and RAF.	[107]
cnk	*connector enhancer of ksr*	CG6556	*cnk* encodes a protein containing several protein-protein interaction domains, suggesting	[106]

			that it brings different signaling molecules together.	
Ave (HYP)	*Aveugle (Hyphen)*	CG30476	Hyphen (Aveugle) is a sterile alpha motif domain-containing protein, which in association with CNK interacts with KSR and that leads to stimulation of the RAS-dependent RAF activating.	[130,131]
alph	*alphabet*	CG1906	*alph* encodes a protein phosphatase 2C (PP2C) family member, which negatively regulates the MAPK signaling pathway.	[132]
14-3-3ε	*14-3-3ε*	CG31196	14-3-3ε is a member of 14-3-3 protein family, an activator of Ras.	[133]
14-3-3ζ	*14-3-3ζ*	CG17870	14-3-3ζ is a member of the 14-3-3 protein family, an activator of Ras with a partly redundant function with 14-3-3ε.	[133]
Cdc37	*Cdc37*	CG12019	A positive regulation of Raf1.	[134]
mago	*mago nashi*	CG9401	A positive effector of *rl (MAPK)* transcription.	[126,135]
eIF4AIII	*eIF4AIII*	CG7483	A positive effector of *rl (MAPK)* transcription.	[126,135]
Cdk12	*Cyclin-dependent kinase 12*	CG7597	Cdk12 is RNA polymerase II C-terminal domain kinase, which provides phosphorylation required for transcription elongation, RNA processing, and splicing; Cdk12 is a modulator of *rl (MAPK)* transcription.	[126,136]
Fip1	*Fip1*	CG1078	FIP1 is a modulator of *rl (MAPK)* transcript processing, probably regulating splicing efficiency; FIP1 yeast ortholog encodes a component of a yeast pre-mRNA polyadenylation factor that directly interacts with poly(A) polymerase.	[126,137]
CG1603		*CG1603*	CG1603 encodes a protein of unknown function that contains MADF type zinc finger domains; modulator of *rl (MAPK)* transcription.	[126]
CG4936		*CG4936*	*CG4936* encodes a zinc finger protein of unknown function that is related to human BCL-6 like transcription factor that is expressed in hematopoietic tissues; effector of MAPK signalling in *Drosophila* acting as MEK downstream .	[126,138]
PTP-ER	*Protein tyrosine phosphatase-ERK/Enhancer of Ras1*	CG9856	Negative regulator of MAPK signalling; transcription of *PTP-ER* is found to be under *CG4936* control.	[126]
gfzf	*GST-containing FLYWCH zinc-finger protein*	CG33546<br>CG10065<br>CG31492	GFZF affects MEK expression, presumably by regulating mek transcription	[126]

		CG31329		

Table 7 JNK signaling pathway components in *D. melanogaster*

Acronym	Gene name	CG number	Function/Description of the gene product	Reference
Src64B	*Src oncogene at 64B*	CG7524	A non-receptor tyrosine kinase of the Src family; an upstream kinase in “canonical” JNK signalling pathway	[108]
Btk29A	*Btk family kinase at 29A*	CG8049	A non-receptor tyrosine kinase of the Src family; an upstream kinase in “canonical” JNK signalling pathway	[108]
Src42A	*Src oncogene at 42A*	CG44128	A non-receptor tyrosine kinase of the Src family; an upstream kinase in “canonical” JNK signalling pathway	[108]
shark	*SH2 ankyrin repeat kinase*	CG18247	Tyrosine kinase Shark plays a crucial role in JNK-mediated dorsal closure, where it acts upstream of JNK	[139]
Dok	*Downstream of kinase*	CG2079	Dok is an adaptor protein of Shark with ability to bind Shark SH2 domains in a tyrosine phosphorylation-dependent fashion; Dok tyrosine phosphorylation is Src dependent	[140]
slpr	*slipper*	CG2272	JUN kinase kinase kinase (JNKKK), known as Mixed Lineage Kinase (MLK)	[112]
msn	*misshapen*	CG16973	JUN kinase kinase kinase kinase (JNKKKK), kinase associated with MLK	[141]
Rac1	*Rac1*	CG2248	A small GTPase; a main activator of MLK	[141]
egr (TNF)	*eiger (tumor necrosis factor)*	CG12919	A TNF superfamily ligand that triggers the *Drosophila* JNK pathway	[142]
wgn (TNFR)	*wengen (tumor necrosis factor receptor)*	CG6531	The receptor of Eiger	[143]
Traf4 (TRAF1)	*TNF-receptor-associated factor 4 (Drosophila homolog of mammalian TRAF1)*	CG3048	Tumor necrosis factor receptor-associated factor, functioning as a signal mediator of cell surface receptor	[144]
Traf6 (TRAF2)	TNF-receptor-associated factor 4 *(Drosophila homolog of mammalian TRAF2)*	CG10961	Tumor necrosis factor receptor-associated factor, functions as a signal mediator of cell surface receptor	[144]
Tab2	*TAK1-associated-binding protein 2*	CG7417	Adaptor protein, linking dTRAF1 to the JNKKK dTAK1	[145]

Tak1	*TGF-β activated kinase 1*	CG18492	JUN kinase kinase kinase (JNKKK)	[146]
Pk92B (ASK1)	*Protein kinase at 92B (Apoptotic signal-regulating kinase 1)*	CG4720	JUN kinase kinase kinase (JNKKK)	[110,147]
Mekk1 (MEKK4)	*Mekk1 (MEKK4)*	CG7717	Stress-induced MAP kinase kinase kinase (MKKK), an activator of JNK pathway	[148]
hep (MKK7)	*Hemipterous (MAP kinase kinase 7)*	CG4353	JUN kinase kinase (JNKK)	[149]
Mkk4	*MAP kinase kinase 4*	CG9738	JUN kinase kinase (JNKK)	[149]
bsk	*basket*	CG5680	Encodes a single *Drosophila* JNK	[150]
Jra (c-Jun)	Jun-related antigen	CG2275	A homolog of mammalian c-Jun; in association with Kayak forms AP-1 transcriptional complex	[151]
kay (c-Fos)	*kayak*	CG33956	A homolog of mammalian c-Fos; in association with Jra forms AP-1 transcriptional complex	[151]
Cka	*Connector of kinase to AP-1*	CG7392	Encodes a scaffold molecule suggested to form a complex with Hemipterous and Basket	[148]
dpp	*decapentaple-gic*	CG9885	*dpp* is a main AP-1 target gene, encoding the *Drosophila* member of transforming growth factor-β (TGF-β) family, a ligand for Dpp signalling	[152,153]
puc	*puckered*	CG7850	A JNK-specific phosphatase that restricts JNK activity in a negative feedback loop	[154,155]
chic	*chickadee*	CG9553	A JNK signaling responsible gene, homolog of vertebrate regulator of actin cytoskeleton profilin	[156]
Mmp1	*Matrix metallo-proteinase 1*	CG4859	A primary function of Mmp1 in the JNK pathway control is to promote basement membrane repair, which in turn may permit cell migration and the restoration of tissue	[113]
Fer1HCH	*Ferritin 1 heavy chain* homologue	CG2216	A JNK signaling responsible gene; free radical scavanger	[104]
MtnA	*Metallothione-in A*	CG9470	A JNK signaling responsible gene; antioxidant	[104]
Sesn	*Sestrin*	CG11299	A JNK signaling responsible gene; antioxidant	[51]
NLaz	*Neural Lazarillo*	CG33126	A JNK signaling responsible gene; lipocalin	[157]
l(2)efl	*lethal (2) essential for life*	CG4533	A JNK signaling target gene; a small heat shock protein, antioxidant	[104]
GstD1	*Glutathione-S-transferase D1 homolog*	CG10045	A JNK signaling target gene; an antioxidant enzyme	[104]

Hsp68	*Heat shock protein 68*	CG5436	A JNK signaling target gene; *Drosophila* HSP70	[104]
Jafrac1	*thioredoxin peroxidase 1*	CG1633	Jafrac1, a JNK signaling target gene, is a *Drosophila* homolog of human Peroxiredoxin II (hPrxII), known to act as a downstream effector of JNK/FOXO signaling in neurons that enhance stress resistance and extends life span	[158]
aop (Yan)	*anterior open*	CG3166	Negative regulator of JNK pathway, which represses transcriptional activity of AP-1 complex	[151]
scaf	*scarface*	CG11066	Inhibitor of JNK pathway, which acts in negative feedback loop as an extracellular pathway regulator	[159]
raw	*raw*	CG12437	Negative regulator of JNK signalling pathway, whose molecular mechanisms remain unclear	[160]
peb	*pebbled*	CG12212	Inhibitor of JNK signalling pathway with an elusive mechanism	[160]

REFERENCES

1. Brogiolo W, Stocker H, Ikeya T, Rintelen F, Fernandez R, et al. (2001) An evolutionarily conserved function of the *Drosophila* insulin receptor and insulin-like peptides in growth control. Curr Biol 11: 213-221.
2. Broughton SJ, Piper MD, Ikeya T, Bass TM, Jacobson J, et al. (2005) Longer lifespan, altered metabolism, and stress resistance in *Drosophila* from ablation of cells making insulin-like ligands. Proc Natl Acad Sci U S A 102: 3105-3110.
3. Grönke S, Clarke DF, Broughton S, Andrews TD, Partridge L (2010) Molecular evolution and functional characterization of *Drosophila* insulin-like peptides. PLoS Genet 6: e1000857.
4. Rulifson EJ, Kim SK, Nusse R (2002) Ablation of insulin-producing neurons in flies: growth and diabetic phenotypes. Science 296: 1118-1120.
5. Toivonen JM, Partridge L (2009) Endocrine regulation of aging and reproduction in *Drosophila*. Mol Cell Endocrinol 299: 39-50.
6. Veenstra JA, Agricola HJ, Sellami A (2008) Regulatory peptides in fruit fly midgut. Cell Tissue Res 334: 499-516.
7. Söderberg JA, Birse RT, Nässel DR (2011) Insulin production and signaling in renal tubules of *Drosophila* is under control of tachykinin-related peptide and regulates stress resistance. PLoS ONE 6: e19866.
8. Okamoto N, Yamanaka N, Yagi Y, Nishida Y, Kataoka H, et al. (2009) A fat body-derived IGF-like peptide regulates postfeeding growth in *Drosophila*. Dev Cell 17: 885-891.
9. Slaidina M, Delanoue R, Grönke S, Partridge L, Leopold P (2009) A *Drosophila* insulin-like peptide promotes growth during nonfeeding states. Dev Cell 17: 874-884.
10. Miguel-Aliaga I, Thor S, Gould AP (2008) Postmitotic specification of *Drosophila* insulinergic neurons from pioneer neurons. PLoS Biol 6: e58.
11. Yang CH, Belawat P, Hafen E, Jan LY, Jan YN (2008) *Drosophila* egg-laying site selection as a system to study simple decision-making processes. Science 319: 1679-1683.
12. Colombani J, Andersen DS, Leopold P (2012) Secreted peptide Dilp8 coordinates *Drosophila* tissue growth with developmental timing. Science 336: 582-585.

13. Garelli A, Gontijo AM, Miguela V, Caparros E, Dominguez M (2012) Imaginal discs secrete insulin-like peptide 8 to mediate plasticity of growth and maturation. Science 336: 579-582.
14. Fernandez R, Tabarini D, Azpiazu N, Frasch M, Schlessinger J (1995) The *Drosophila* insulin receptor homolog: a gene essential for embryonic development encodes two receptor isoforms with different signaling potential. EMBO J 14: 3373-3384.
15. Oldham S, Hafen E (2003) Insulin/IGF and target of rapamycin signaling: a TOR de force in growth control. Trends Cell Biol 13: 79-85.
16. Clancy DJ, Gems D, Harshman LG, Oldham S, Stocker H, et al. (2001) Extension of life-span by loss of CHICO, a *Drosophila* insulin receptor substrate protein. Science 292: 104-106.
17. Slack C, Werz C, Wieser D, Alic N, Foley A, et al. (2010) Regulation of lifespan, metabolism, and stress responses by the *Drosophila* SH2B protein, Lnk. PLoS Genet 6: e1000881.
18. Werz C, Kohler K, Hafen E, Stocker H (2009) The *Drosophila* SH2B family adaptor Lnk acts in parallel to chico in the insulin signaling pathway. PLoS genetics 5: e1000596.
19. Leevers SJ (2001) Growth control: invertebrate insulin surprises! Current biology : CB 11: R209-212.
20. Williams KD, Busto M, Suster ML, So AK, Ben-Shahar Y, et al. (2006) Natural variation in *Drosophila melanogaster* diapause due to the insulin-regulated PI3-kinase. Proc Natl Acad Sci USA 103: 15911-15915.
21. Weinkove D, Leevers SJ, MacDougall LK, Waterfield MD (1997) p60 is an adaptor for the *Drosophila* phosphoinositide 3-kinase, Dp110. J Biol Chem 272: 14606-14610.
22. Fuss B, Becker T, Zinke I, Hoch M (2006) The cytohesin Steppke is essential for insulin signalling in *Drosophila*. Nature 444: 945-948.
23. Cho KS, Lee JH, Kim S, Kim D, Koh H, et al. (2001) *Drosophila* phosphoinositide-dependent kinase-1 regulates apoptosis and growth via the phosphoinositide 3-kinase-dependent signaling pathway. Proceedings of the National Academy of Sciences of the United States of America 98: 6144-6149.
24. Teleman AA, Chen YW, Cohen SM (2005) 4E-BP functions as a metabolic brake used under stress conditions but not during normal growth. Genes & development 19: 1844-1848.
25. Puig O, Marr MT, Ruhf ML, Tjian R (2003) Control of cell number by Drosophila FOXO: downstream and feedback regulation of the insulin receptor pathway. Genes & development 17: 2006-2020.
26. Frame S, Cohen P, Biondi RM (2001) A common phosphate binding site explains the unique substrate specificity of GSK3 and its inactivation by phosphorylation. Molecular cell 7: 1321-1327.
27. Dombradi V, Matko J, Kiss Z, Kiss L, Friedrich P, et al. (1986) Structural and functional properties of *Drosophila melanogaster* phosphorylase: comparison with the rabbit skeletal muscle enzyme. Comparative biochemistry and physiology B, Comparative biochemistry 84: 537-543.
28. Okamura T, Shimizu H, Nagao T, Ueda R, Ishii S (2007) ATF-2 regulates fat metabolism in *Drosophila*. Molecular biology of the cell 18: 1519-1529.
29. Wang B, Moya N, Niessen S, Hoover H, Mihaylova MM, et al. (2011) A hormone-dependent module regulating energy balance. Cell 145: 596-606.
30. Grönke S, Mildner A, Fellert S, Tennagels N, Petry S, et al. (2005) Brummer lipase is an evolutionary conserved fat storage regulator in *Drosophila*. Cell Metabol 1: 323-330.
31. Furuyama T, Kitayama K, Yamashita H, Mori N (2003) Forkhead transcription factor FOXO1 (FKHR)-dependent induction of PDK4 gene expression in skeletal muscle during energy deprivation. The Biochemical journal 375: 365-371.
32. Sedding DG (2008) FoxO transcription factors in oxidative stress response and ageing--a new fork on the way to longevity? Biological chemistry 389: 279-283.
33. van der Horst A, Burgering BM (2007) Stressing the role of FoxO proteins in lifespan and disease. Nature reviews Molecular cell biology 8: 440-450.

34. Tettweiler G, Miron M, Jenkins M, Sonenberg N, Lasko PF (2005) Starvation and oxidative stress resistance in *Drosophila* are mediated through the eIF4E-binding protein, d4E-BP. Genes & development 19: 1840-1843.
35. Teleman AA (2010) Molecular mechanisms of metabolic regulation by insulin in *Drosophila*. Biochem J 425: 13-26.
36. Xie Q, Chen J, Yuan Z (2012) Post-translational regulation of FOXO. Acta biochimica et biophysica Sinica 44: 897-901.
37. Palanker L, Tennessen JM, Lam G, Thummel CS (2009) *Drosophila* HNF4 regulates lipid mobilization and beta-oxidation. Cell metabolism 9: 228-239.
38. Altarejos JY, Montminy M (2011) CREB and the CRTC co-activators: sensors for hormonal and metabolic signals. Nature reviews Molecular cell biology 12: 141-151.
39. Karpac J, Jasper H (2011) Metabolic homeostasis: HDACs take center stage. Cell 145: 497-499.
40. Colombani J, Raisin S, Pantalacci S, Radimerski T, Montagne J, et al. (2003) A nutrient sensor mechanism controls *Drosophila* growth. Cell 114: 739-749.
41. Goberdhan DC, Meredith D, Boyd CA, Wilson C (2005) PAT-related amino acid transporters regulate growth via a novel mechanism that does not require bulk transport of amino acids. Development 132: 2365-2375.
42. Martin JF, Hersperger E, Simcox A, Shearn A (2000) minidiscs encodes a putative amino acid transporter subunit required non-autonomously for imaginal cell proliferation. Mech Dev 92: 155-167.
43. Wullschleger S, Loewith R, Hall MN (2006) TOR signaling in growth and metabolism. Cell 124: 471-484.
44. Kim E, Goraksha-Hicks P, Li L, Neufeld TP, Guan KL (2008) Regulation of TORC1 by Rag GTPases in nutrient response. Nature cell biology 10: 935-945.
45. Yang H, Gong R, Xu Y (2013) Control of cell growth: Rag GTPases in activation of TORC1. Cellular and molecular life sciences : CMLS 70: 2873-2885.
46. Hall DJ, Grewal SS, de la Cruz AF, Edgar BA (2007) Rheb-TOR signaling promotes protein synthesis, but not glucose or amino acid import, in Drosophila. BMC Biol 5: 10.
47. Zhang Y, Gao X, Saucedo LJ, Ru B, Edgar BA, et al. (2003) Rheb is a direct target of the tuberous sclerosis tumour suppressor proteins. Nature cell biology 5: 578-581.
48. Pan DA, Hardie DG (2002) A homologue of AMP-activated protein kinase in Drosophila melanogaster is sensitive to AMP and is activated by ATP depletion. Biochem J 367: 179-186.
49. Inoki K, Zhu T, Guan KL (2003) TSC2 mediates cellular energy response to control cell growth and survival. Cell 115: 577-590.
50. Inoki K, Ouyang H, Zhu T, Lindvall C, Wang Y, et al. (2006) TSC2 integrates Wnt and energy signals via a coordinated phosphorylation by AMPK and GSK3 to regulate cell growth. Cell 126: 955-968.
51. Lee JH, Budanov AV, Park EJ, Birse R, Kim TE, et al. (2010) Sestrin as a Feedback Inhibitor of TOR That Prevents Age-Related Pathologies. Science 327: 1223-1228.
52. Hernandez G, Altmann M, Sierra JM, Urlaub H, Diez del Corral R, et al. (2005) Functional analysis of seven genes encoding eight translation initiation factor 4E (eIF4E) isoforms in Drosophila. Mech Dev 122: 529-543.
53. Grewal SS, Evans JR, Edgar BA (2007) *Drosophila* TIF-IA is required for ribosome synthesis and cell growth and is regulated by the TOR pathway. The Journal of cell biology 179: 1105-1113.
54. Chang YY, Neufeld TP (2010) Autophagy takes flight in *Drosophila*. FEBS letters 584: 1342-1349.
55. Dekanty A, Lavista-Llanos S, Irisarri M, Oldham S, Wappner P (2005) The insulin-PI3K/TOR pathway induces a HIF-dependent transcriptional response in *Drosophila* by promoting nuclear localization of HIF-alpha/Sima. Journal of cell science 118: 5431-5441.
56. Reiling JH, Hafen E (2004) The hypoxia-induced paralogs Scylla and Charybdis inhibit growth by down-regulating S6K activity upstream of TSC in Drosophila. Genes Dev 18: 2879-2892.
57. Teleman AA, Hietakangas V, Sayadian AC, Cohen SM (2008) Nutritional control of protein biosynthetic capacity by insulin via Myc in *Drosophila*. Cell Metab 7: 21-32.

58. Guertin DA, Guntur KV, Bell GW, Thoreen CC, Sabatini DM (2006) Functional genomics identifies TOR-regulated genes that control growth and division. Current biology : CB 16: 958-970.
59. Zaffran S, Chartier A, Gallant P, Astier M, Arquier N, et al. (1998) A Drosophila RNA helicase gene, pitchoune, is required for cell growth and proliferation and is a potential target of d-Myc. Development 125: 3571-3584.
60. Migeon JC, Garfinkel MS, Edgar BA (1999) Cloning and characterization of peter pan, a novel Drosophila gene required for larval growth. Mol Biol Cell 10: 1733-1744.
61. Giordano E, Peluso I, Senger S, Furia M (1999) minifly, a Drosophila gene required for ribosome biogenesis. J Cell Biol 144: 1123-1133.
62. Kim SK, Rulifson EJ (2004) Conserved mechanisms of glucose sensing and regulation by *Drosophila* corpora cardiaca cells. Nature 431: 316-320.
63. Lee G, Park JH (2004) Hemolymph sugar homeostasis and starvation-induced hyperactivity affected by genetic manipulations of the adipokinetic hormone-encoding gene in *Drosophila melanogaster*. Genetics 167: 311-323.
64. Staubli F, Jorgensen TJ, Cazzamali G, Williamson M, Lenz C, et al. (2002) Molecular identification of the insect adipokinetic hormone receptors. Proc Natl Acad Sci U S A 99: 3446-3451.
65. Brubaker-Purkey BJ, Woodruff RI (2013) Vitellogenesis in the fruit fly, Drosophila melanogaster: antagonists demonstrate that the PLC, IP3/DAG, PK-C pathway is triggered by calmodulin. J Insect Sci 13: 68.
66. Gäde G, Auerswald L (2003) Mode of action of neuropeptides from the adipokinetic hormone family. Gen Comp Endocrinol 132: 10-20.
67. Baumbach J, Xu Y, Hehlert P, Kuhnlein RP (2014) Galphaq, Ggamma1 and Plc21C control *Drosophila* body fat storage. Journal of genetics and genomics 41: 283-292.
68. Bahri SM, Chia W (1994) DPhK-gamma, a putative Drosophila kinase with homology to vertebrate phosphorylase kinase gamma subunits: molecular characterisation of the gene and phenotypic analysis of loss of function mutants. Mol Gen Genet 245: 588-597.
69. Tick G, Cserpan I, Dombradi V, Mechler BM, Torok I, et al. (1999) Structural and functional characterization of the Drosophila glycogen phosphorylase gene. Biochem Biophys Res Commun 257: 34-43.
70. Beller M, Bulankina AV, Hsiao HH, Urlaub H, Jackle H, et al. (2010) PERILIPIN-dependent control of lipid droplet structure and fat storage in Drosophila. Cell Metab 12: 521-532.
71. Patel RT, Soulages JL, Hariharasundaram B, Arrese EL (2005) Activation of the lipid droplet controls the rate of lipolysis of triglycerides in the insect fat body. J Biol Chem 280: 22624-22631.
72. Bi J, Xiang Y, Chen H, Liu Z, Gronke S, et al. (2012) Opposite and redundant roles of the two Drosophila perilipins in lipid mobilization. J Cell Sci 125: 3568-3577.
73. Miyoshi H, Souza SC, Zhang HH, Strissel KJ, Christoffolete MA, et al. (2006) Perilipin promotes hormone-sensitive lipase-mediated adipocyte lipolysis via phosphorylation-dependent and -independent mechanisms. J Biol Chem 281: 15837-15844.
74. Sztalryd C, Xu G, Dorward H, Tansey JT, Contreras JA, et al. (2003) Perilipin A is essential for the translocation of hormone-sensitive lipase during lipolytic activation. J Cell Biol 161: 1093-1103.
75. Kuhnlein RP (2011) The contribution of the Drosophila model to lipid droplet research. Progress in lipid research 50: 348-356.
76. Buch S, Melcher C, Bauer M, Katzenberger J, Pankratz MJ (2008) Opposing effects of dietary protein and sugar regulate a transcriptional target of *Drosophila* insulin-like peptide signaling. Cell Metab 7: 321-332.
77. Agaisse H, Perrimon N (2004) The roles of JAK/STAT signaling in Drosophila immune responses. Immunol Rev 198: 72-82.
78. Bach EA, Vincent S, Zeidler MP, Perrimon N (2003) A sensitized genetic screen to identify novel regulators and components of the Drosophila janus kinase/signal transducer and activator of transcription pathway. Genetics 165: 1149-1166.
79. Arbouzova NI, Zeidler MP (2006) JAK/STAT signalling in Drosophila: insights into conserved regulatory and cellular functions. Development 133: 2605-2616.

80. Ghiglione C, Devergne O, Georgenthum E, Carballes F, Medioni C, et al. (2002) The *Drosophila* cytokine receptor Domeless controls border cell migration and epithelial polarization during oogenesis. Development 129: 5437-5447.
81. Baeg GH, Zhou R, Perrimon N (2005) Genome-wide RNAi analysis of JAK/STAT signaling components in Drosophila. Genes Dev 19: 1861-1870.
82. Rawlings JS, Rosler KM, Harrison DA (2004) The JAK/STAT signaling pathway. Journal of cell science 117: 1281-1283.
83. Stec WJ, Zeidler MP (2011) Drosophila SOCS Proteins. J Signal Transduct 2011: 894510.
84. Stec W, Vidal O, Zeidler MP (2013) Drosophila SOCS36E negatively regulates JAK/STAT pathway signaling via two separable mechanisms. Molecular Biology of the Cell 24: 3000-3009.
85. Karsten P, Hader S, Zeidler MP (2002) Cloning and expression of *Drosophila* SOCS36E and its potential regulation by the JAK/STAT pathway. Mechanisms of development 117: 343-346.
86. Betz A, Lampen N, Martinek S, Young MW, Darnell JE, Jr. (2001) A Drosophila PIAS homologue negatively regulates stat92E. Proc Natl Acad Sci U S A 98: 9563-9568.
87. Agaisse H, Petersen UM, Boutros M, Mathey-Prevot B, Perrimon N (2003) Signaling role of hemocytes in Drosophila JAK/STAT-dependent response to septic injury. Dev Cell 5: 441-450.
88. Gronholm J, Kaustio M, Myllymaki H, Kallio J, Saarikettu J, et al. (2012) Not4 enhances JAK/STAT pathway-dependent gene expression in Drosophila and in human cells. FASEB J 26: 1239-1250.
89. Müller P, Kuttenkeuler D, Gesellchen V, Zeidler MP, Boutros M (2005) Identification of JAK/STAT signalling components by genome-wide RNA interference. Nature 436: 871-875.
90. Coste F, Kemp C, Bobezeau V, Hetru C, Kellenberger C, et al. (2012) Crystal structure of Diedel, a marker of the immune response of Drosophila melanogaster. PLoS One 7: e33416.
91. Kallio J, Myllymaki H, Gronholm J, Armstrong M, Vanha-aho LM, et al. (2010) Eye transformer is a negative regulator of Drosophila JAK/STAT signaling. FASEB J 24: 4467-4479.
92. Zhang Y, You J, Ren W, Lin X (2013) Drosophila glypicans Dally and Dally-like are essential regulators for JAK/STAT signaling and Unpaired distribution in eye development. Dev Biol 375: 23-32.
93. Ekengren S, Hultmark D (2001) A family of Turandot-related genes in the humoral stress response of Drosophila. Biochem Biophys Res Commun 284: 998-1003.
94. Wang HZ, Brown J, Gao SG, Liang S, Jotwani R, et al. (2013) The Role of JAK-3 in Regulating TLR-Mediated Inflammatory Cytokine Production in Innate Immune Cells. Journal of Immunology 191: 1164-1174.
95. Taniai K, Inceoglu AB, Yukuhiro K, Hammock BD (2003) Characterization and cDNA cloning of a clofibrate-inducible microsomal epoxide hydrolase in *Drosophila melanogaster*. Eur J Biochem 270: 4696-4705.
96. Leatherman JL, Dinardo S (2008) Zfh-1 controls somatic stem cell self-renewal in the Drosophila testis and nonautonomously influences germline stem cell self-renewal. Cell Stem Cell 3: 44-54.
97. Postigo AA, Ward E, Skeath JB, Dean DC (1999) zfh-1, the Drosophila homologue of ZEB, is a transcriptional repressor that regulates somatic myogenesis. Mol Cell Biol 19: 7255-7263.
98. Yan R, Small S, Desplan C, Dearolf CR, Darnell JE, Jr. (1996) Identification of a Stat gene that functions in Drosophila development. Cell 84: 421-430.
99. Flaherty MS, Salis P, Evans CJ, Ekas LA, Marouf A, et al. (2010) chinmo Is a Functional Effector of the JAK/STAT Pathway that Regulates Eye Development, Tumor Formation, and Stem Cell Self-Renewal in Drosophila. Developmental Cell 18: 556-568.
100. Dostert C, Jouanguy E, Irving P, Troxler L, Galiana-Arnoux D, et al. (2005) The Jak-STAT signaling pathway is required but not sufficient for the antiviral response of drosophila. Nat Immunol 6: 946-953.

101. Bou Aoun R, Hetru C, Troxler L, Doucet D, Ferrandon D, et al. (2011) Analysis of thioester-containing proteins during the innate immune response of Drosophila melanogaster. J Innate Immun 3: 52-64.
102. Schaber MD, O'Hara MB, Garsky VM, Mosser SC, Bergstrom JD, et al. (1990) Polyisoprenylation of Ras in vitro by a farnesyl-protein transferase. J Biol Chem 265: 14701-14704.
103. Stevens LM, Frohnhofer HG, Klingler M, Nusslein-Volhard C (1990) Localized requirement for torso-like expression in follicle cells for development of terminal anlagen of the Drosophila embryo. Nature 346: 660-663.
104. Wang MC, Bohmann D, Jasper H (2003) JNK signaling confers tolerance to oxidative stress and extends lifespan in Drosophila. Dev Cell 5: 811-816.
105. Valanne S, Wang JH, Ramet M (2011) The Drosophila Toll signaling pathway. J Immunol 186: 649-656.
106. Therrien M, Wong AM, Rubin GM (1998) CNK, a RAF-binding multidomain protein required for RAS signaling. Cell 95: 343-353.
107. Therrien M, Chang HC, Solomon NM, Karim FD, Wassarman DA, et al. (1995) KSR, a novel protein kinase required for RAS signal transduction. Cell 83: 879-888.
108. Tateno M, Nishida Y, Adachi-Yamada T (2000) Regulation of JNK by Src during Drosophila development. Science 287: 324-327.
109. Wellbrock C, Karasarides M, Marais R (2004) The RAF proteins take centre stage. Nat Rev Mol Cell Biol 5: 875-885.
110. Wassarman DA, Solomon NM, Rubin GM (1996) Pk92B: a Drosophila melanogaster protein kinase that belongs to the MEKK family. Gene 169: 283-284.
111. Tsuda L, Inoue YH, Yoo MA, Mizuno M, Hata M, et al. (1993) A protein kinase similar to MAP kinase activator acts downstream of the raf kinase in Drosophila. Cell 72: 407-414.
112. Stronach B, Perrimon N (2002) Activation of the JNK pathway during dorsal closure in Drosophila requires the mixed lineage kinase, slipper. Genes Dev 16: 377-387.
113. Stevens LJ, Page-McCaw A (2012) A secreted MMP is required for reepithelialization during wound healing. Molecular Biology of the Cell 23: 1068-1079.
114. Martin JR, Raibaud A, Ollo R (1994) Terminal pattern elements in Drosophila embryo induced by the torso-like protein. Nature 367: 741-745.
115. Johnson TK, Crossman T, Foote KA, Henstridge MA, Saligari MJ, et al. (2013) Torso-like functions independently of Torso to regulate Drosophila growth and developmental timing. Proc Natl Acad Sci U S A 110: 14688-14692.
116. Casanova J, Struhl G (1989) Localized surface activity of torso, a receptor tyrosine kinase, specifies terminal body pattern in Drosophila. Genes Dev 3: 2025-2038.
117. Henstridge MA, Johnson TK, Warr CG, Whisstock JC (2014) Trunk cleavage is essential for Drosophila terminal patterning and can occur independently of Torso-like. Nat Commun 5: 3419.
118. Hart AC, Kramer H, Van Vactor DL, Jr., Paidhungat M, Zipursky SL (1990) Induction of cell fate in the Drosophila retina: the bride of sevenless protein is predicted to contain a large extracellular domain and seven transmembrane segments. Genes Dev 4: 1835-1847.
119. Kohyama-Koganeya A, Kim YJ, Miura M, Hirabayashi Y (2008) A Drosophila orphan G protein-coupled receptor BOSS functions as a glucose-responding receptor: loss of boss causes abnormal energy metabolism. Proc Natl Acad Sci U S A 105: 15328-15333.
120. Basler K, Hafen E (1989) Ubiquitous expression of sevenless: position-dependent specification of cell fate. Science 243: 931-934.
121. Basler K, Hafen E (1989) Dynamics of Drosophila eye development and temporal requirements of sevenless expression. Development 107: 723-731.
122. Olivier JP, Raabe T, Henkemeyer M, Dickson B, Mbamalu G, et al. (1993) A Drosophila SH2-SH3 adaptor protein implicated in coupling the sevenless tyrosine kinase to an activator of Ras guanine nucleotide exchange, Sos. Cell 73: 179-191.
123. Perkins LA, Larsen I, Perrimon N (1992) corkscrew encodes a putative protein tyrosine phosphatase that functions to transduce the terminal signal from the receptor tyrosine kinase torso. Cell 70: 225-236.

124. Buday L, Downward J (1993) Epidermal growth factor regulates p21ras through the formation of a complex of receptor, Grb2 adapter protein, and Sos nucleotide exchange factor. Cell 73: 611-620.
125. Karim FD, Chang HC, Therrien M, Wassarman DA, Laverty T, et al. (1996) A screen for genes that function downstream of Ras1 during Drosophila eye development. Genetics 143: 315-329.
126. Ashton-Beaucage D, Udell CM, Gendron P, Sahmi M, Lefrancois M, et al. (2014) A functional screen reveals an extensive layer of transcriptional and splicing control underlying RAS/MAPK signaling in Drosophila. PLoS Biol 12: e1001809.
127. Leevers SJ, Paterson HF, Marshall CJ (1994) Requirement for Ras in Raf activation is overcome by targeting Raf to the plasma membrane. Nature 369: 411-414.
128. Baccarini M (2005) Second nature: biological functions of the Raf-1 "kinase". FEBS Lett 579: 3271-3277.
129. Lim YM, Nishizawa K, Nishi Y, Tsuda L, Inoue YH, et al. (1999) Genetic analysis of rolled, which encodes a Drosophila mitogen-activated protein kinase. Genetics 153: 763-771.
130. Douziech M, Sahmi M, Laberge G, Therrien M (2006) A KSR/CNK complex mediated by HYP, a novel SAM domain-containing protein, regulates RAS-dependent RAF activation in Drosophila. Genes Dev 20: 807-819.
131. Claperon A, Therrien M (2007) KSR and CNK: two scaffolds regulating RAS-mediated RAF activation. Oncogene 26: 3143-3158.
132. Baril C, Therrien M (2006) Alphabet, a Ser/Thr phosphatase of the protein phosphatase 2C family, negatively regulates RAS/MAPK signaling in Drosophila. Dev Biol 294: 232-245.
133. Chang HC, Rubin GM (1997) 14-3-3 epsilon positively regulates Ras-mediated signaling in Drosophila. Genes Dev 11: 1132-1139.
134. Grammatikakis N, Lin JH, Grammatikakis A, Tsichlis PN, Cochran BH (1999) p50(cdc37) acting in concert with Hsp90 is required for Raf-1 function. Mol Cell Biol 19: 1661-1672.
135. Ashton-Beaucage D, Udell CM, Lavoie H, Baril C, Lefrancois M, et al. (2010) The exon junction complex controls the splicing of MAPK and other long intron-containing transcripts in Drosophila. Cell 143: 251-262.
136. Phatnani HP, Greenleaf AL (2006) Phosphorylation and functions of the RNA polymerase II CTD. Genes Dev 20: 2922-2936.
137. Preker PJ, Lingner J, Minvielle-Sebastia L, Keller W (1995) The FIP1 gene encodes a component of a yeast pre-mRNA polyadenylation factor that directly interacts with poly(A) polymerase. Cell 81: 379-389.
138. Zhang W, Mi J, Li N, Sui L, Wan T, et al. (2001) Identification and characterization of DPZF, a novel human BTB/POZ zinc finger protein sharing homology to BCL-6. Biochem Biophys Res Commun 282: 1067-1073.
139. Fernandez R, Takahashi F, Liu Z, Steward R, Stein D, et al. (2000) The Drosophila shark tyrosine kinase is required for embryonic dorsal closure. Genes Dev 14: 604-614.
140. Biswas R, Stein D, Stanley ER (2006) Drosophila Dok is required for embryonic dorsal closure. Development 133: 217-227.
141. Garlena RA, Gonda RL, Green AB, Pileggi RM, Stronach B (2010) Regulation of mixed-lineage kinase activation in JNK-dependent morphogenesis. J Cell Sci 123: 3177-3188.
142. Igaki T, Kanda H, Yamamoto-Goto Y, Kanuka H, Kuranaga E, et al. (2002) Eiger, a TNF superfamily ligand that triggers the Drosophila JNK pathway. EMBO J 21: 3009-3018.
143. Kauppila S, Maaty WS, Chen P, Tomar RS, Eby MT, et al. (2003) Eiger and its receptor, Wengen, comprise a TNF-like system in Drosophila. Oncogene 22: 4860-4867.
144. Cha GH, Cho KS, Lee JH, Kim M, Kim E, et al. (2003) Discrete functions of TRAF1 and TRAF2 in Drosophila melanogaster mediated by c-Jun N-terminal kinase and NF-kappaB-dependent signaling pathways. Mol Cell Biol 23: 7982-7991.
145. Geuking P, Narasimamurthy R, Basler K (2005) A genetic screen targeting the tumor necrosis factor/Eiger signaling pathway: identification of Drosophila TAB2 as a functionally conserved component. Genetics 171: 1683-1694.
146. Mihaly J, Kockel L, Gaengel K, Weber U, Bohmann D, et al. (2001) The role of the Drosophila TAK homologue dTAK during development. Mech Dev 102: 67-79.

147. Kuranaga E, Kanuka H, Igaki T, Sawamoto K, Ichijo H, et al. (2002) Reaper-mediated inhibition of DIAP1-induced DTRAF1 degradation results in activation of JNK in Drosophila. Nat Cell Biol 4: 705-710.
148. Chen W, White MA, Cobb MH (2002) Stimulus-specific requirements for MAP3 kinases in activating the JNK pathway. J Biol Chem 277: 49105-49110.
149. Geuking P, Narasimamurthy R, Lemaitre B, Basler K, Leulier F (2009) A non-redundant role for Drosophila Mkk4 and hemipterous/Mkk7 in TAK1-mediated activation of JNK. PLoS One 4: e7709.
150. Riesgo-Escovar JR, Jenni M, Fritz A, Hafen E (1996) The *Drosophila* Jun-N-terminal kinase is required for cell morphogenesis but not for DJun-dependent cell fate specification in the eye. Genes & Development 10: 2759-2768.
151. Riesgo-Escovar JR, Hafen E (1997) Drosophila Jun kinase regulates expression of decapentaplegic via the ETS-domain protein Aop and the AP-1 transcription factor DJun during dorsal closure. Genes Dev 11: 1717-1727.
152. Raftery LA, Sutherland DJ (1999) TGF-beta family signal transduction in Drosophila development: from Mad to Smads. Dev Biol 210: 251-268.
153. Fernandez BG, Arias AM, Jacinto A (2007) Dpp signalling orchestrates dorsal closure by regulating cell shape changes both in the amnioserosa and in the epidermis. Mech Dev 124: 884-897.
154. Martin-Blanco E, Gampel A, Ring J, Virdee K, Kirov N, et al. (1998) puckered encodes a phosphatase that mediates a feedback loop regulating JNK activity during dorsal closure in Drosophila. Genes Dev 12: 557-570.
155. McEwen DG, Peifer M (2005) Puckered, a Drosophila MAPK phosphatase, ensures cell viability by antagonizing JNK-induced apoptosis. Development 132: 3935-3946.
156. Jasper H, Benes V, Schwager C, Sauer S, Clauder-Munster S, et al. (2001) The genomic response of the *Drosophila* embryo to JNK signaling. Dev Cell 1: 579-586.
157. Hull-Thompson J, Muffat J, Sanchez D, Walker DW, Benzer S, et al. (2009) Control of metabolic homeostasis by stress signaling is mediated by the lipocalin NLaz. PLoS Genet 5: e1000460.
158. Lee KS, Iijima-Ando K, Iijima K, Lee WJ, Lee JH, et al. (2009) JNK/FOXO-mediated neuronal expression of fly homologue of peroxiredoxin II reduces oxidative stress and extends life span. J Biol Chem 284: 29454-29461.
159. Rousset R, Bono-Lauriol S, Gettings M, Suzanne M, Speder P, et al. (2010) The Drosophila serine protease homologue Scarface regulates JNK signalling in a negative-feedback loop during epithelial morphogenesis. Development 137: 2177-2186.
160. Reed BH, Wilk R, Lipshitz HD (2001) Downregulation of Jun kinase signaling in the amnioserosa is essential for dorsal closure of the Drosophila embryo. Curr Biol 11: 1098-1108.
