# Supplementary material for: Slowed aging during reproductive dormancy is reflected in genome-wide transcriptome changes in Drosophila melanogaster
Source: BMC Genomics. 2016 Jan 13;17:50. doi: 10.1186/s12864-016-2383-1 (PMC4711038; doi:10.1186/s12864-016-2383-1)
Supplement: Additional file 10: Figure S5. — Results of overrepresentation test of GO biological process terms for comparisons between our genome-wide transcription study of D. melanogaster dormancy and aging studies. 3wD, three weeks diapause (whole animals) our study; Aging, aging response genes [136] and candidate lifespan extending genes [135]. Comparison based on genes in the Venn diagram in Fig. 10a. Term enrichment analysis of Groups A – D are shown separately. (PDF 405 kb) [file 12864_2016_2383_MOESM10_ESM.pdf]

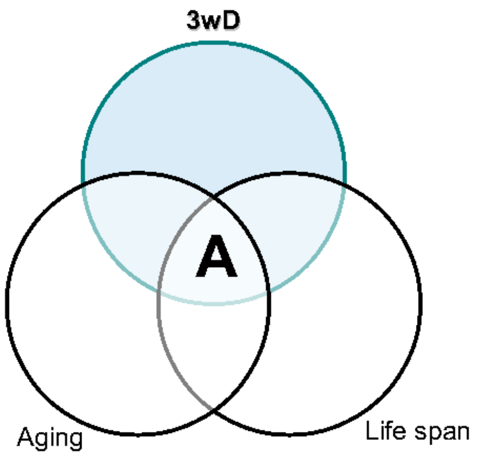

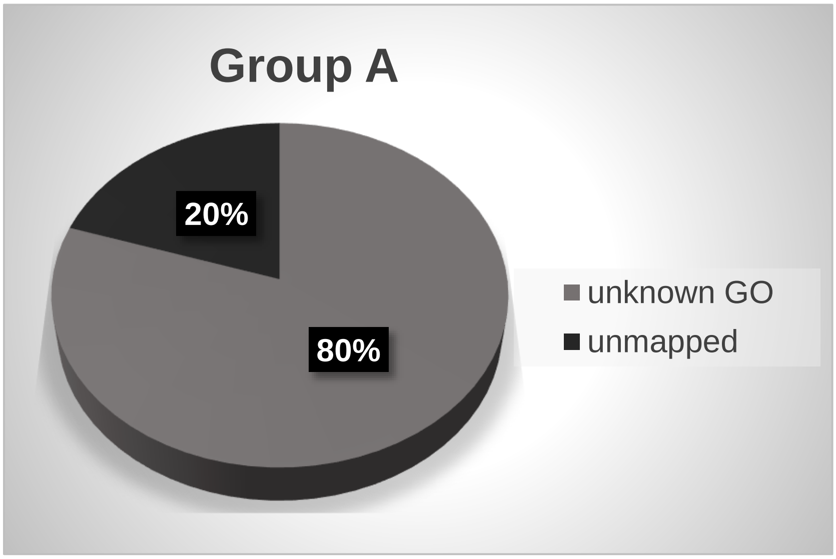

Term	Backgr. frequency	Sample frequency	Fold enrichment	+/-	P-value
Unclassified	3044	32	1.03	+	0.00E+00

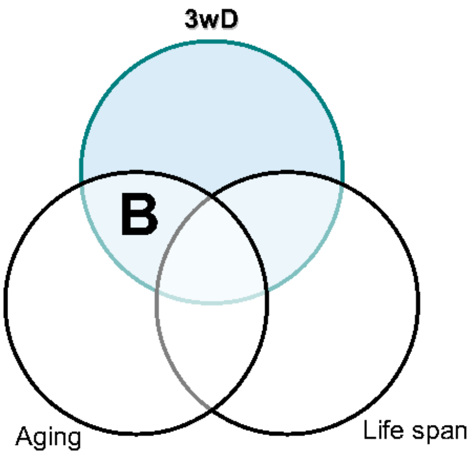

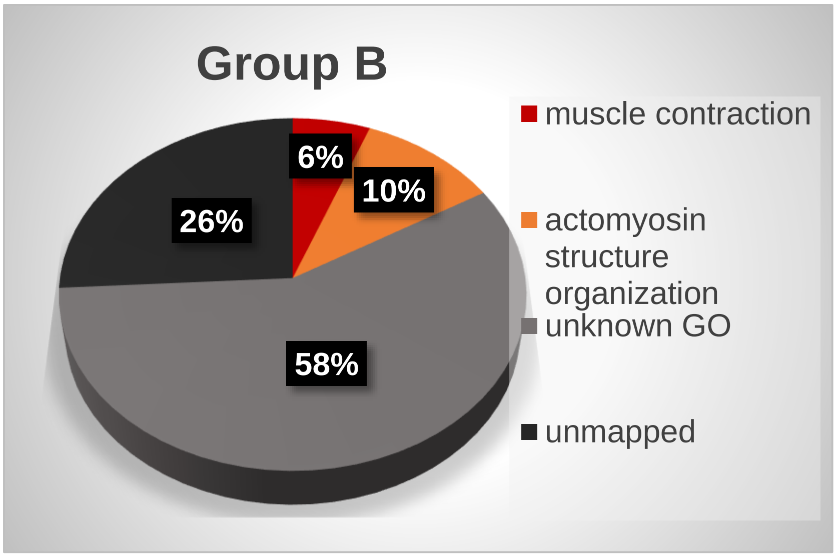

Term	Backgr. frequency	Sample frequency	Fold enrichment	+/-	P-value
Muscle contraction (GO:0006936)	15	5	>5	+	3.74E-02
Muscle system process (GO:0003012)	22	6	>5	+	1.59E-02
Myofibril assembly (GO:0030239)	40	7	>5	+	4.64E-02
Striated muscle cell development(GO:0055002)	54	8	>5	+	3.84E-02
Muscle cell development (GO:0055001)	54	8	>5	+	3.84E-02
Actomyosin structure organization (GO:0031032)	66	9	>5	+	2.16E-02
Unclassified	3044	49	0.82	-	0.00E+00

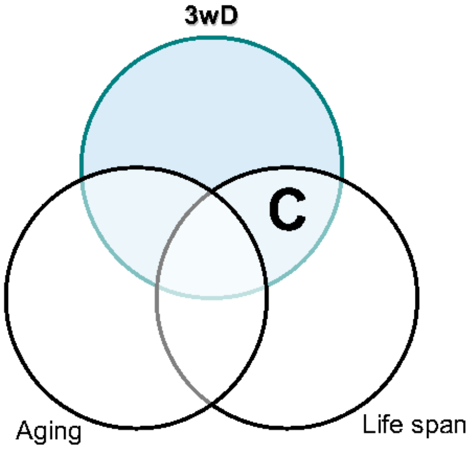

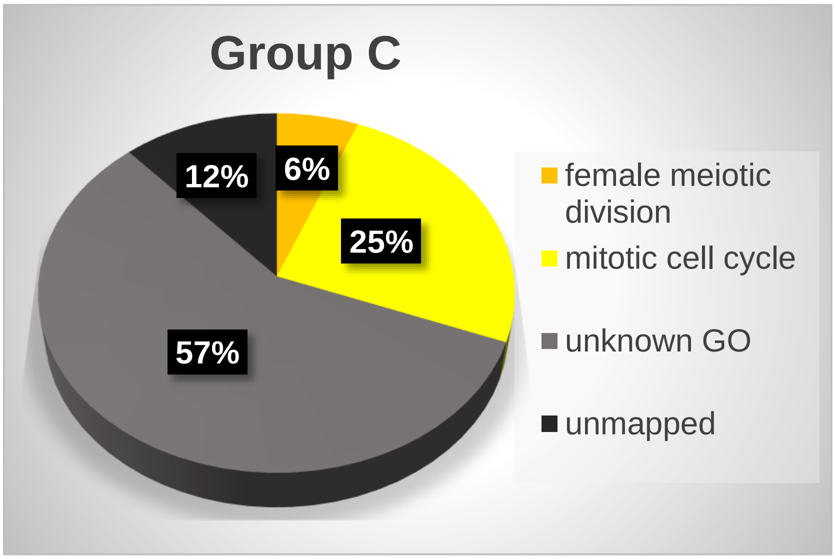

Term	Backgr. frequency	Sample frequency	Fold enrichment	+/-	P-value
Spindle assembly involved in female meiosis (GO:0007056)	15	6	>5	+	4.87E-02
Female meiotic division(GO:0007143)	73	12	4.69	+	3.87E-02
Meiotic cell cycle (GO:0051321)	538	40	2.12	+	2.49E-02
Cell cycle (GO:0007049)	729	54	2.11	+	6.99E-04
Cell cycle process (GO:0022402)	674	49	2.07	+	4.38E-03
Unclassified	3044	114	1.07	+	0.00E+00

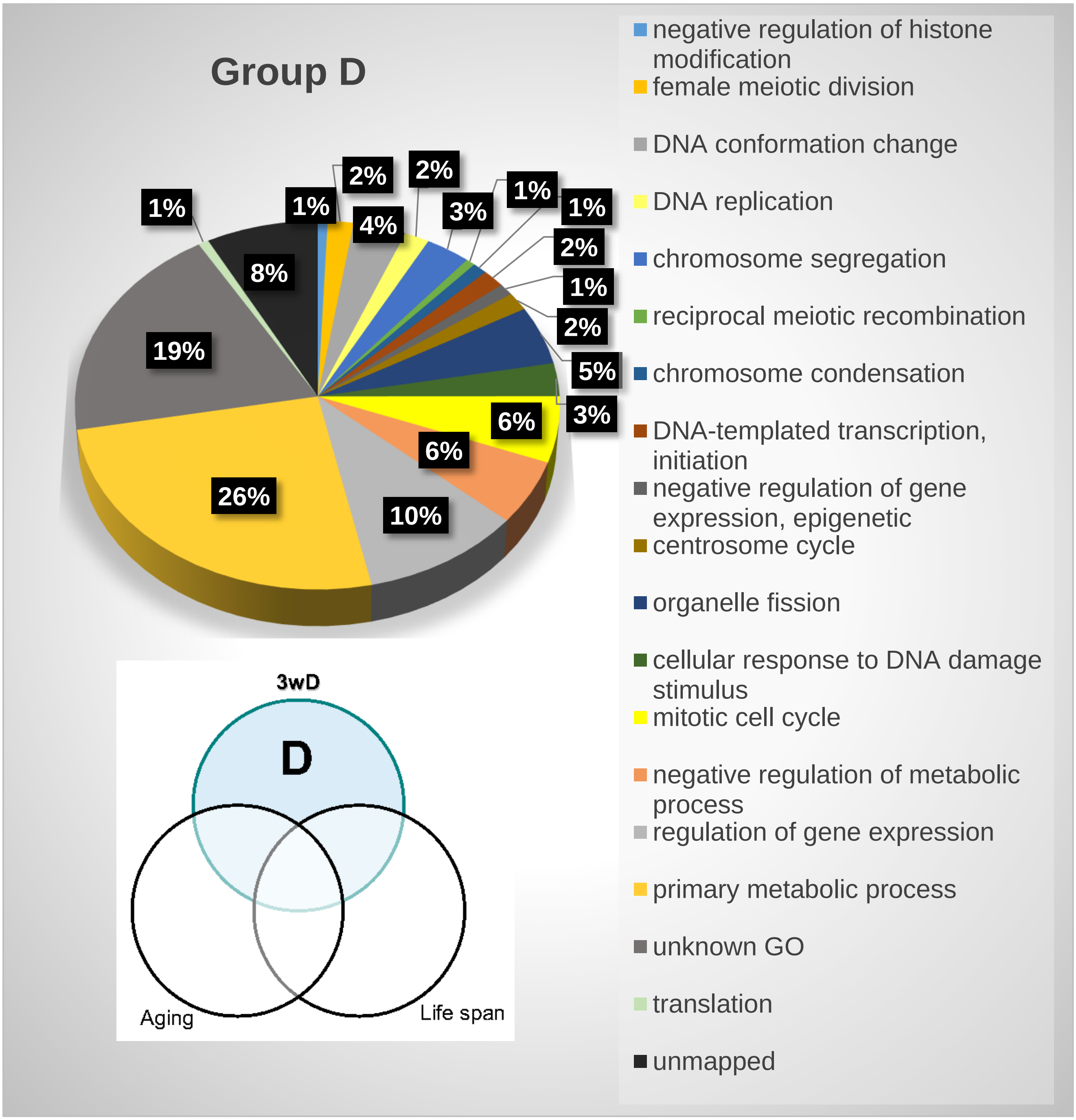

Term	Backgr. Frequency	Sample frequency	Fold Enrichment	+/-	P value
Negative regulation of histone modification	5	18	> 5	+	4.42E-15
Negative regulation of chromatin modification	15	22	> 5	+	7.15E-11
Nucleosome assembly	65	65	> 5	+	1.95E-26
Female meiosis chromosome segregation	34	32	> 5	+	2.46E-11
Chromatin assembly	73	67	> 5	+	3.20E-25
Negative regulation of chromosome organization	30	27	> 5	+	7.89E-09
Protein-DNA complex assembly	85	76	> 5	+	3.64E-28
Regulation of histone modification	26	23	> 5	+	4.31E-07
Nucleosome organization	80	66	> 5	+	2.62E-22
DNA packaging	122	93	4.68	+	1.04E-29
Protein-DNA complex subunit organization	107	78	4.48	+	2.22E-23
DNA conformation change	134	96	4.4	+	1.08E-28

Term	Backgr. Frequency	Sample frequency	Fold Enrichment	+/-	P value
DNA conformation change	134	96	4.4	+	1.08E-28
Cell cycle DNA replication	23	16	4.27	+	5.53E-03
Meiotic chromosome segregation	65	44	4.16	+	3.08E-11
Female meiotic division	73	47	3.96	+	2.36E-11
DNA-dependent DNA replication	57	35	3.77	+	2.02E-07
Nuclear chromosome segregation	131	79	3.71	+	9.90E-19
Reciprocal meiotic recombination	29	17	3.6	+	2.49E-02
Chromosome condensation	47	27	3.53	+	1.06E-04
Reciprocal DNA recombination	30	17	3.48	+	3.81E-02
DNA replication	80	45	3.46	+	7.84E-09
Chromosome segregation	151	82	3.34	+	8.37E-17
DNA-templated transcription, initiation	76	41	3.31	+	2.43E-07
Mitotic sister chromatid segregation	72	38	3.24	+	2.09E-06
Sister chromatid segregation	79	39	3.03	+	7.74E-06
Chromatin assembly or disassembly	140	69	3.03	+	8.59E-12
Negative regulation of gene expression, epigenetic	56	27	2.96	+	3.01E-03
Chromatin silencing	55	26	2.9	+	6.68E-03
Regulation of chromatin organization	72	34	2.9	+	2.15E-04
Regulation of chromatin modification	68	31	2.8	+	1.63E-03
Negative regulation of organelle organization	75	34	2.79	+	5.43E-04
Negative regulation of protein modification process	83	37	2.74	+	2.43E-04
Centrosome duplication	84	37	2.71	+	3.25E-04
Regulation of chromosome organization	102	44	2.65	+	3.98E-05
Centrosome cycle	98	42	2.63	+	9.96E-05
DNA recombination	61	26	2.62	+	3.94E-02
Meiosis I	73	31	2.61	+	6.80E-03
DNA metabolic process	243	103	2.6	+	3.65E-14
Microtubule organizing center organization	130	54	2.55	+	3.55E-06
Cellular macromolecular complex assembly	234	95	2.49	+	8.64E-12
Meiotic nuclear division	180	73	2.49	+	1.43E-08
Meiotic cell cycle process	186	74	2.44	+	2.44E-08
Meiotic cell cycle	186	74	2.44	+	2.44E-08
Centrosome organization	126	50	2.44	+	5.83E-05
RNA biosynthetic process	177	68	2.36	+	7.01E-07
Nuclear division	354	135	2.34	+	1.90E-15
Nucleic acid-templated transcription	174	66	2.33	+	2.10E-06
Transcription, DNA-templated	174	66	2.33	+	2.10E-06
Chromosome organization	480	182	2.33	+	1.97E-21
Protein complex assembly	280	106	2.33	+	1.85E-11
Negative regulation of cellular component organization	128	48	2.3	+	5.58E-04
Protein complex biogenesis	283	106	2.3	+	3.60E-11
Organelle fission	361	135	2.3	+	9.01E-15
Gene silencing	99	37	2.3	+	1.40E-02
DNA repair	127	46	2.23	+	2.49E-03
Chromatin organization	314	113	2.21	+	6.43E-11
Macromolecular complex assembly	349	124	2.18	+	8.39E-12
Mitotic nuclear division	207	69	2.05	+	1.30E-04
Nucleobase-containing compound biosynthetic process	321	103	1.97	+	5.46E-07

Term	Backgr. Frequency	Sample frequency	Fold Enrichment	+/-	P value
Regulation of mitotic cell cycle	221	70	1.95	+	6.69E-04
Cell cycle process	674	213	1.94	+	3.45E-16
Cell cycle	729	227	1.91	+	1.07E-16
Nucleic acid metabolic process	865	269	1.91	+	2.63E-20
Regulation of cell cycle process	201	62	1.9	+	7.21E-03
Regulation of cell cycle	350	104	1.83	+	2.32E-05
Heterocycle biosynthetic process	393	114	1.78	+	1.62E-05
Aromatic compound biosynthetic process	389	111	1.75	+	5.91E-05
Negative regulation of nucleobase-containing compound metabolic process	309	88	1.75	+	1.81E-03
Regulation of organelle organization	284	80	1.73	+	8.46E-03
Mitotic cell cycle	538	151	1.72	+	5.05E-07
Negative regulation of RNA biosynthetic process	275	77	1.72	+	1.58E-02
Negative regulation of transcription, DNA-templated	275	77	1.72	+	1.58E-02
Negative regulation of nucleic acid-templated transcription	275	77	1.72	+	1.58E-02
Microtubule cytoskeleton organization	405	113	1.71	+	1.37E-04
Negative regulation of gene expression	405	113	1.71	+	1.37E-04
Mitotic cell cycle process	484	135	1.71	+	6.93E-06
Negative regulation of macromolecule biosynthetic process	323	90	1.71	+	3.26E-03
Negative regulation of cellular macromolecule biosynthetic process	323	90	1.71	+	3.26E-03
Cellular response to DNA damage stimulus	274	76	1.7	+	2.48E-02
Negative regulation of cellular biosynthetic process	332	92	1.7	+	3.11E-03
Negative regulation of biosynthetic process	332	92	1.7	+	3.11E-03
Protein complex subunit organization	580	160	1.7	+	4.80E-07
Negative regulation of RNA metabolic process	288	79	1.69	+	2.44E-02
Organic cyclic compound biosynthetic process	431	117	1.67	+	3.20E-04
Negative regulation of nitrogen compound metabolic process	354	96	1.67	+	4.47E-03
Nucleobase-containing compound metabolic process	1149	309	1.65	+	1.39E-14
RNA metabolic process	644	173	1.65	+	6.03E-07
Negative regulation of macromolecule metabolic process	531	141	1.63	+	5.90E-05
Positive regulation of macromolecule biosynthetic process	328	87	1.63	+	3.03E-02
Regulation of cellular macromolecule biosynthetic process	941	246	1.61	+	1.04E-09
Regulation of macromolecule biosynthetic process	943	246	1.6	+	1.29E-09
Macromolecular complex subunit organization	866	224	1.59	+	3.48E-08
Heterocycle metabolic process	1240	320	1.59	+	8.47E-13
Negative regulation of metabolic process	566	146	1.58	+	1.83E-04
Cellular aromatic compound metabolic process	1273	326	1.57	+	1.17E-12
Regulation of biosynthetic process	997	255	1.57	+	3.49E-09
Regulation of cellular biosynthetic process	996	254	1.57	+	5.22E-09
Regulation of RNA biosynthetic process	846	215	1.56	+	4.21E-07
Regulation of transcription, DNA-templated	846	215	1.56	+	4.21E-07
Regulation of nucleic acid-templated transcription	846	215	1.56	+	4.21E-07
Cellular component assembly	752	191	1.56	+	5.04E-06

Term	Backgr. Frequency	Sample frequency	Fold Enrichment	+/-	P value
Regulation of cellular protein metabolic process	429	108	1.55	+	2.59E-02
Organic cyclic compound metabolic process	1343	335	1.53	+	1.41E-11
Regulation of gene expression	1080	268	1.52	+	2.02E-08
Regulation of nucleobase-containing compound metabolic process	974	241	1.52	+	3.43E-07
Regulation of protein metabolic process	454	112	1.52	+	4.16E-02
Negative regulation of cellular metabolic process	485	119	1.51	+	2.88E-02
Regulation of nitrogen compound metabolic process	1079	264	1.5	+	1.20E-07
Positive regulation of macromolecule metabolic process	521	127	1.5	+	1.98E-02
Single-organism organelle organization	1080	263	1.5	+	2.07E-07
Cellular component biogenesis	826	201	1.5	+	4.65E-05
Regulation of cellular component organization	639	155	1.49	+	2.58E-03
Regulation of RNA metabolic process	923	222	1.48	+	1.98E-05
Regulation of macromolecule metabolic process	1423	341	1.47	+	1.33E-09
Macromolecule biosynthetic process	708	169	1.47	+	2.15E-03
Cellular macromolecule biosynthetic process	700	167	1.47	+	2.58E-03
Organelle organization	1539	365	1.46	+	6.42E-10
Regulation of primary metabolic process	1378	322	1.44	+	1.20E-07
Regulation of cellular metabolic process	1421	326	1.41	+	6.69E-07
Negative regulation of biological process	1141	258	1.39	+	2.19E-04
Cellular nitrogen compound metabolic process	1516	342	1.39	+	1.43E-06
Regulation of metabolic process	1620	362	1.37	+	1.18E-06
Negative regulation of cellular process	1011	225	1.37	+	4.82E-03
Cellular component organization	2359	520	1.35	+	3.67E-10
Positive regulation of biological process	1100	242	1.35	+	4.34E-03
Positive regulation of cellular process	969	212	1.34	+	2.84E-02
Nervous system development	1550	337	1.34	+	8.82E-05
Cellular component organization or biogenesis	2420	526	1.34	+	2.57E-09
Organic substance biosynthetic process	1107	239	1.33	+	1.84E-02
Regulation of cellular process	2901	626	1.33	+	2.06E-11
Neurogenesis	1398	299	1.31	+	2.42E-03
Cellular macromolecule metabolic process	2233	477	1.31	+	5.37E-07
Regulation of biological process	3154	673	1.31	+	1.10E-11
Nitrogen compound metabolic process	1782	379	1.31	+	1.07E-04
Biological regulation	3431	720	1.29	+	2.45E-11
System development	2184	457	1.29	+	2.09E-05
Multicellular organismal development	2737	557	1.25	+	1.38E-05
Cell differentiation	2207	446	1.24	+	1.78E-03
Single-organism cellular process	4961	1000	1.24	+	9.04E-14
Cellular developmental process	2276	458	1.24	+	1.90E-03
Anatomical structure development	2973	588	1.22	+	2.56E-04
Cellular process	6231	1227	1.21	+	2.66E-16
Developmental process	3281	645	1.21	+	1.03E-04
Single-organism developmental process	3257	639	1.21	+	1.68E-04
Cellular metabolic process	3189	625	1.2	+	2.97E-04
Macromolecule metabolic process	2815	551	1.2	+	2.89E-03
Single-organism process	6411	1225	1.17	+	2.10E-11
Primary metabolic process	3500	667	1.17	+	4.89E-03
Single-multicellular organism process	3282	625	1.17	+	1.38E-02

Term	Backgr. Frequency	Sample frequency	Fold Enrichment	+/-	P value
Organic substance metabolic process	3812	710	1.14	+	4.27E-02
Unclassified	3044	506	1.02	+	0.00E+00
Translation	290	19	0.4	-	6.04E-03
Amide biosynthetic process	309	20	0.4	-	2.22E-03
Peptide biosynthetic process	299	19	0.39	-	2.40E-03
