# Supplementary material for: Slowed aging during reproductive dormancy is reflected in genome-wide transcriptome changes in Drosophila melanogaster
Source: BMC Genomics. 2016 Jan 13;17:50. doi: 10.1186/s12864-016-2383-1 (PMC4711038; doi:10.1186/s12864-016-2383-1)
Supplement: Additional file 11: Figure S6. — Term enrichment analysis of GO biological process terms for comparisons among four genome-wide transcription studies of D. melanogaster. 3wD, three weeks diapause (whole animals) our study; 1wD, abdomens sampled after one week diapause [140]; cold intolerance [141] and starvation [143]. Comparison based on genes in the Venn diagram in Fig. 10b. Term analysis of Groups A – H are shown separately. (PDF 1267 kb) [file 12864_2016_2383_MOESM11_ESM.pdf]

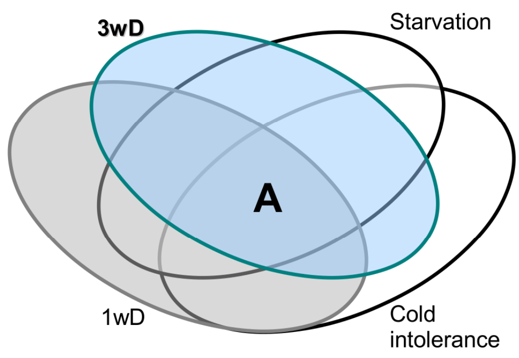

8 genes

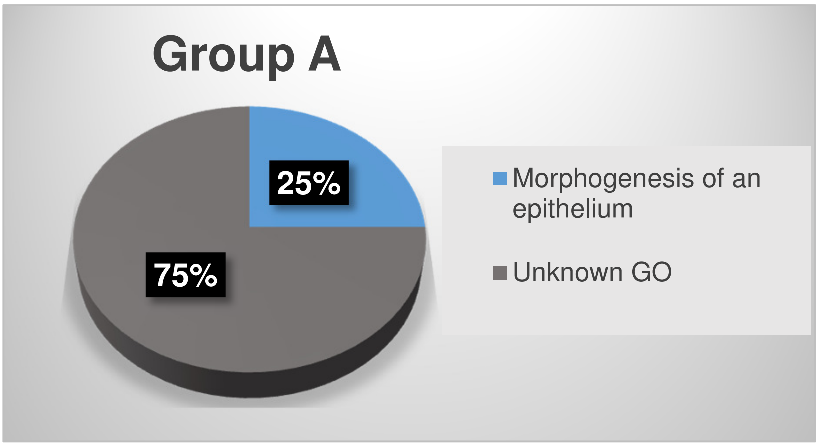

Term	Backgr. frequency	Sample frequency	Expected	+/-	P-value
Tube morphogenesis (GO:0035239)	123	2	7.22E-02	+	2.20E-03
Tube development (GO:0035295)	204	2	1.20E-01	+	5.91E-03
Embryonic morphogenesis (GO:0048598)	231	2	1.36E-01	+	7.52E-03
Morphogenesis of an epithelium (GO:0002009)	285	2	1.67E-01	+	1.13E-02
Tissue morphogenesis (GO:0048729)	311	2	1.83E-01	+	1.33E-02
Embryo development (GO:0009790)	509	2	2.99E-01	+	3.36E-02

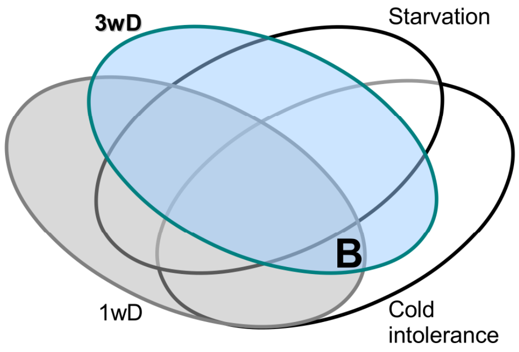

12 genes

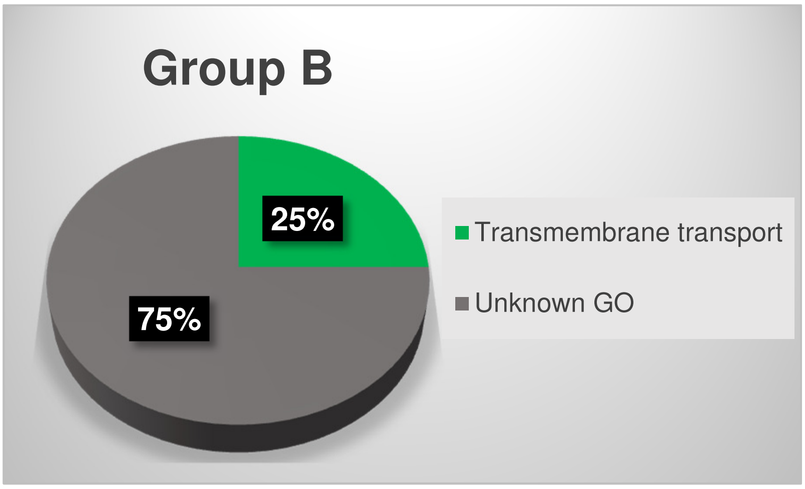

Term	Backgr. frequency	Sample frequency	Expected	+/-	P-value
Transmembrane transport (GO:0055085)	427	3	3.76E-01	+	1.10E-02

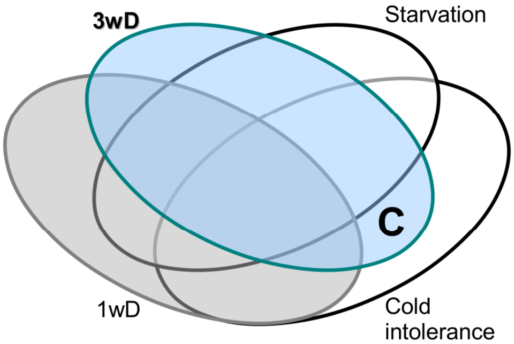

72 genes

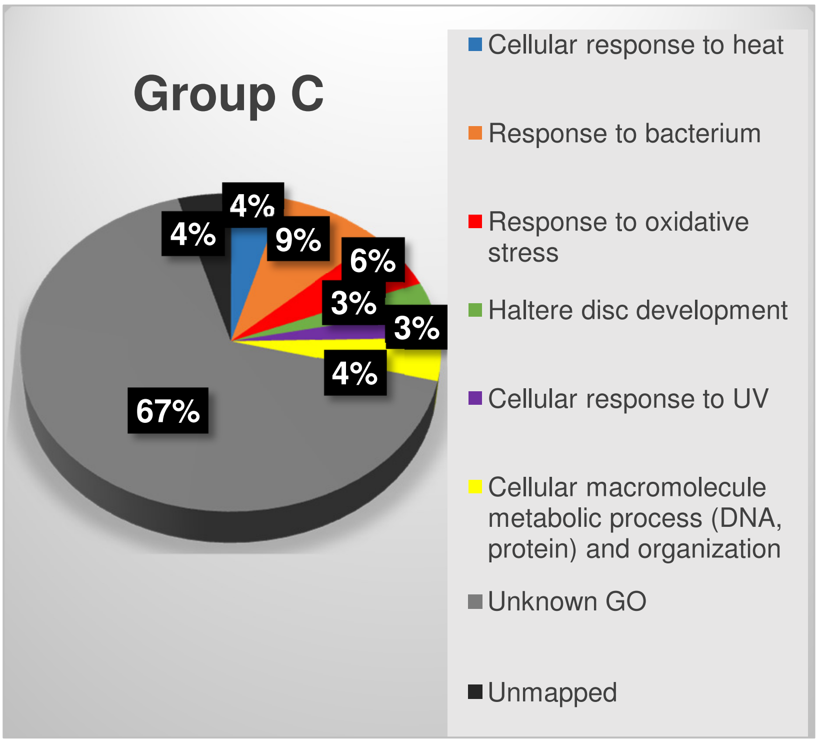

Term	Backgr. frequency	Sample frequency	Expected	+/-	P-value
Cellular response to heat (GO:0034605)	23	3	1.17E-01	+	4.64E-03
Cellular macromolecule metabolic process (GO:0044260)	2232	2	1.13E+01	-	9.10E-03
Response to bacterium (GO:0009617)	202	6	1.02E+00	+	1.15E-02
Response to oxidative stress (GO:0006979)	92	4	4.66E-01	+	2.54E-02
Haltere disc development (GO:0035216)	11	2	5.57E-02	+	2.95E-02
Positive regulation of biosynthetic process of Antibacterial peptides active against Gram-negative bacteria (GO:0006964)	11	2	5.57E-02	+	2.95E-02
Cellular component organization or biogenesis (GO:0071840)	2286	3	1.16E+01	-	3.38E-02
Cellular response to UV (GO:0034644)	13	2	6.58E-02	+	4.09E-02
Regulation of biosynthetic process of Antibacterial peptides active against Gram-negative bacteria (GO:0002813)	13	2	6.58E-02	+	4.09E-02
Cellular component organization (GO:0016043)	2229	3	1.13E+01	-	4.40E-02

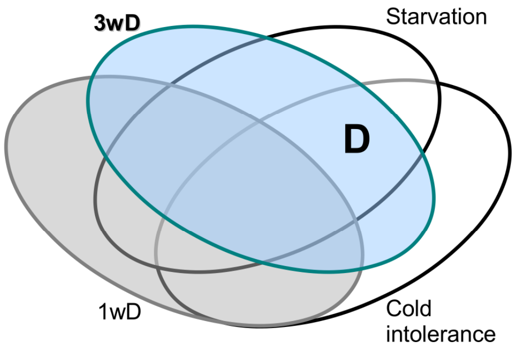

24 genes

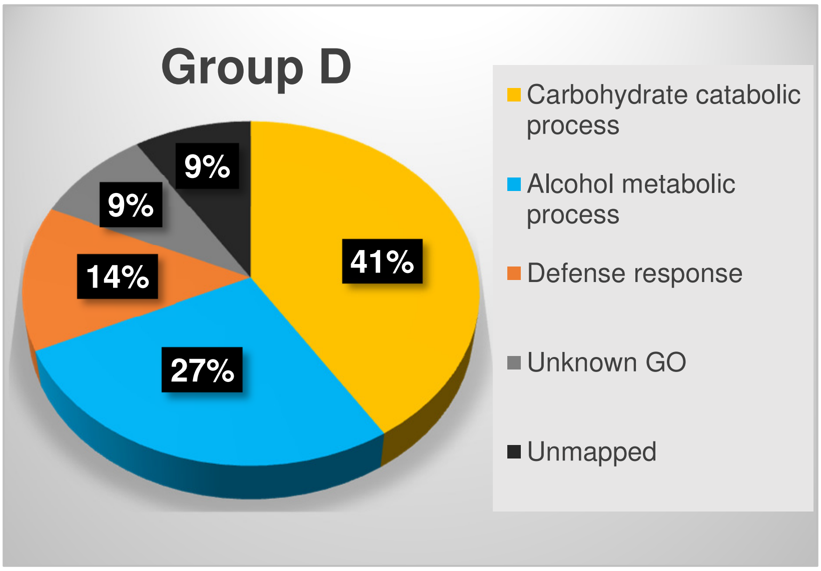

Term	Backgr. frequency	Sample frequency	Expected	+/-	P-value
Cellular carbohydrate catabolic process (GO:0044275)	4	2	6.46E-03	+	5.95E-05
Alcohol metabolic process (GO:0006066)	57	3	9.20E-02	+	3.19E-04
Organic hydroxy compound catabolic process (GO:1901616)	12	2	1.94E-02	+	5.31E-04
Alcohol catabolic process (GO:0046164)	12	2	1.94E-02	+	5.31E-04
Small molecule catabolic process (GO:0044282)	73	3	1.18E-01	+	6.59E-04
Carbohydrate metabolic process (GO:0005975)	273	4	4.41E-01	+	2.65E-03
Small molecule metabolic process (GO:0044281)	739	6	1.19E+00	+	2.68E-03
Single-organism catabolic process (GO:0044712)	286	4	4.62E-01	+	3.15E-03
Organic hydroxy compound metabolic process (GO:1901615)	131	3	2.12E-01	+	3.58E-03
Single-organism metabolic process (GO:0044710)	1859	9	3.00E+00	+	4.53E-03
Single-organism carbohydrate catabolic process (GO:0044724)	38	2	6.14E-02	+	5.20E-03
Carbohydrate catabolic process (GO:0016052)	41	2	6.62E-02	+	6.03E-03
Cellular carbohydrate metabolic process (GO:0044262)	42	2	6.78E-02	+	6.32E-03
Single-organism carbohydrate metabolic process (GO:0044723)	181	3	2.92E-01	+	8.97E-03
Response to ethanol (GO:0045471)	62	2	1.00E-01	+	1.35E-02
Oxidation-reduction process (GO:0055114)	531	4	8.58E-01	+	2.88E-02
Response to alcohol (GO:0097305)	98	2	1.58E-01	+	3.26E-02
Organic substance catabolic process (GO:1901575)	559	4	9.03E-01	+	3.44E-02
Defense response (GO:0006952)	330	3	5.33E-01	+	4.65E-02

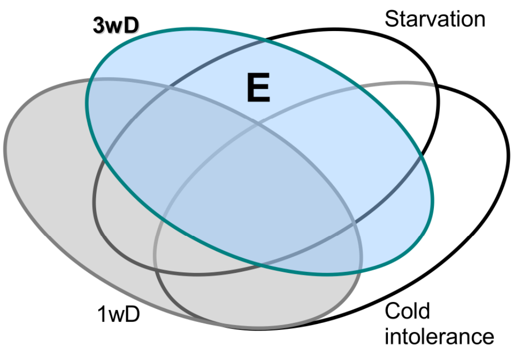

520 genes

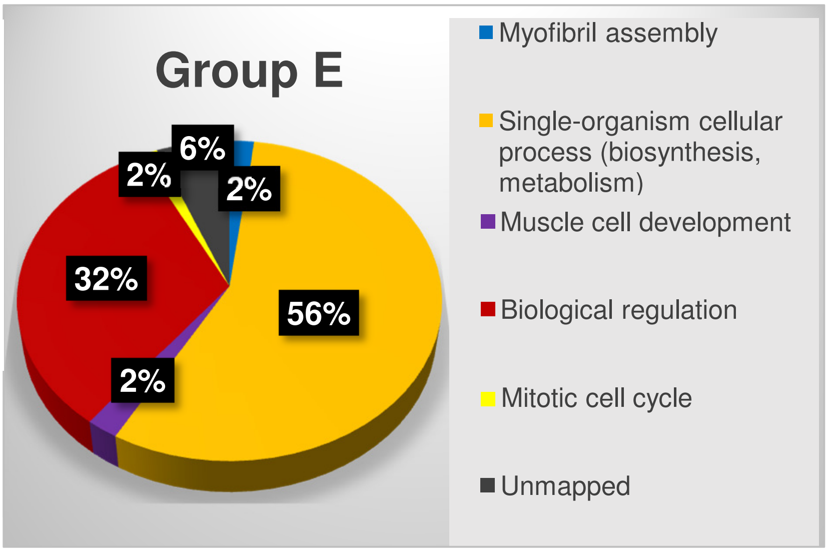

Term	Backgr. frequency	Sample frequency	Expected	+/-	P-value
Myofibril assembly (GO:0030239)	35	10	1.26E+00	+	2.39E-04
Single-organism process (GO:0044699)	6248	276	2.25E+02	+	6.30E-04
Single-organism cellular process (GO:0044763)	4790	220	1.72E+02	+	1.60E-03
Striated muscle cell development (GO:0055002)	46	10	1.65E+00	+	2.59E-03
Muscle cell development (GO:0055001)	46	10	1.65E+00	+	2.59E-03
Actomyosin structure organization (GO:0031032)	59	11	2.12E+00	+	3.86E-03
Biological regulation (GO:0065007)	3256	157	1.17E+02	+	7.63E-03
Cellular process (GO:0009987)	5996	260	2.16E+02	+	1.03E-02
Single-multicellular organism process (GO:0044707)	3203	152	1.15E+02	+	2.53E-02
Single-organism biosynthetic process (GO:0044711)	483	35	1.74E+01	+	2.72E-02
Single-organism metabolic process (GO:0044710)	1859	97	6.69E+01	+	3.00E-02
Mitotic cell cycle, embryonic (GO:0045448)	29	7	1.04E+00	+	3.04E-02

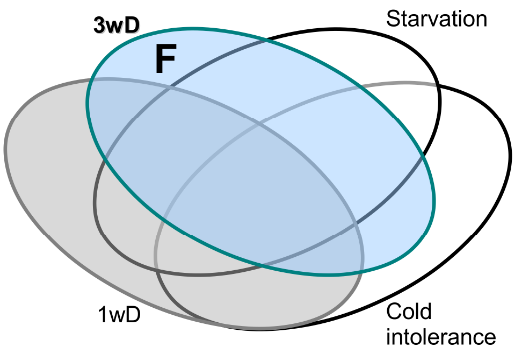

2279 genes

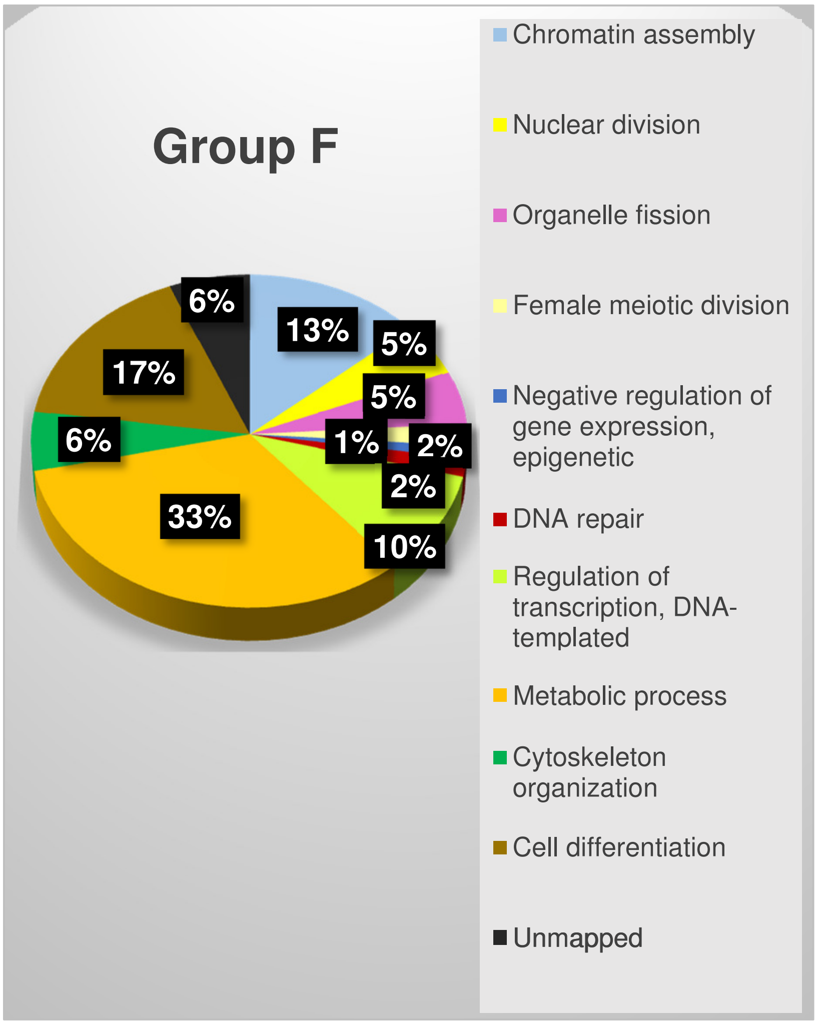

Term	Backgr. frequency	Sample frequency	Expected	+/-	P-value
DNA metabolic process (GO:0006259)	333	168	5.39E+01	+	6.95E-34
DNA packaging (GO:0006323)	120	85	1.94E+01	+	1.74E-25
Nucleic acid metabolic process (GO:0090304)	930	296	1.50E+02	+	3.35E-25
Nucleosome assembly (GO:0006334)	67	62	1.08E+01	+	5.51E-24
DNA conformation change (GO:0071103)	130	86	2.10E+01	+	7.90E-24
Protein-DNA complex assembly (GO:0065004)	85	68	1.37E+01	+	6.29E-23
Chromatin assembly (GO:0031497)	75	63	1.21E+01	+	3.26E-22
Nucleosome organization (GO:0034728)	78	63	1.26E+01	+	2.42E-21
Protein-DNA complex subunit organization (GO:0071824)	102	71	1.65E+01	+	1.31E-20
Chromosome organization (GO:0051276)	454	170	7.34E+01	+	4.78E-20
Nucleobase-containing compound metabolic process (GO:0006139)	1225	337	1.98E+02	+	1.24E-18
Chromosome segregation (GO:0007059)	143	81	2.31E+01	+	2.86E-18
Nuclear division (GO:0000280)	318	129	5.14E+01	+	1.89E-17
Cellular aromatic compound metabolic process (GO:0006725)	1353	358	2.19E+02	+	2.56E-17
Organelle fission (GO:0048285)	323	129	5.22E+01	+	6.56E-17
Organic cyclic compound metabolic process (GO:1901360)	1421	370	2.30E+02	+	6.59E-17

Term	Backgr. frequency	Sample frequency	Expected	+/-	P-value
Cellular nitrogen compound metabolic process (GO:0034641)	1350	355	2.18E+02	+	9.97E-17
Heterocycle metabolic process (GO:0046483)	1319	347	2.13E+02	+	2.67E-16
Female meiotic division (GO:0007143)	70	52	1.13E+01	+	7.60E-16
Negative regulation of histone modification (GO:0031057)	5	18	8.09E-01	+	1.37E-15
Meiotic cell cycle process (GO:1903046)	139	72	2.25E+01	+	4.52E-14
Meiotic nuclear division (GO:0007126)	155	76	2.51E+01	+	1.10E-13
Negative regulation of chromatin modification (GO:1903309)	7	18	1.13E+00	+	4.31E-13
Meiotic cell cycle (GO:0051321)	174	80	2.81E+01	+	5.57E-13
Protein complex assembly (GO:0006461)	264	104	4.27E+01	+	6.44E-13
Nitrogen compound metabolic process (GO:0006807)	1625	392	2.63E+02	+	7.03E-13
Meiotic chromosome segregation (GO:0045132)	61	44	9.86E+00	+	9.68E-13
Protein complex biogenesis (GO:0070271)	266	104	4.30E+01	+	1.04E-12
Cell cycle process (GO:0022402)	617	186	9.98E+01	+	1.29E-12
Female meiosis chromosome segregation (GO:0016321)	33	32	5.34E+00	+	3.20E-12
Cell cycle (GO:0007049)	698	202	1.13E+02	+	3.71E-12
Cellular macromolecule metabolic process (GO:0044260)	2232	502	3.61E+02	+	4.63E-12
Cell division (GO:0051301)	469	151	7.58E+01	+	4.66E-12
Macromolecular complex assembly (GO:0065003)	329	118	5.32E+01	+	4.72E-12
Chromatin organization (GO:0006325)	307	109	4.96E+01	+	9.84E-11
Cellular process (GO:0009987)	5996	1140	9.70E+02	+	1.73E-10
Cellular macromolecular complex assembly (GO:0034622)	221	86	3.57E+01	+	3.98E-10
Negative regulation of chromosome organization (GO:2001251)	24	25	3.88E+00	+	6.88E-10
Chromatin assembly or disassembly (GO:0006333)	148	66	2.39E+01	+	7.26E-10
Organelle organization (GO:0006996)	1440	339	2.33E+02	+	2.09E-09
Single-organism cellular process (GO:0044763)	4790	924	7.75E+02	+	2.39E-08
Single-organism organelle organization (GO:1902589)	972	241	1.57E+02	+	5.33E-08
Regulation of chromosome organization (GO:0033044)	60	36	9.70E+00	+	5.89E-08
Negative regulation of protein modification process (GO:0031400)	35	27	5.66E+00	+	7.03E-08
Regulation of histone modification (GO:0031056)	23	22	3.72E+00	+	7.79E-08
Microtubule organizing center organization (GO:0031023)	126	55	2.04E+01	+	1.22E-07
Cellular component organization (GO:0016043)	2229	473	3.60E+02	+	2.22E-07
Macromolecular complex subunit organization (GO:0043933)	821	208	1.33E+02	+	2.23E-07

Term	Backgr. frequency	Sample frequency	Expected	+/-	P-value
Regulation of chromatin organization (GO:1902275)	32	25	5.17E+00	+	2.69E-07
Protein complex subunit organization (GO:0071822)	546	151	8.83E+01	+	3.01E-07
Sister chromatid segregation (GO:0000819)	74	39	1.20E+01	+	3.79E-07
Mitotic sister chromatid segregation (GO:0000070)	68	37	1.10E+01	+	4.52E-07
Macromolecule metabolic process (GO:0043170)	2870	583	4.64E+02	+	8.00E-07
Centrosome organization (GO:0051297)	122	52	1.97E+01	+	8.51E-07
Cellular metabolic process (GO:0044237)	3194	639	5.17E+02	+	9.56E-07
Regulation of chromatin modification (GO:1903308)	29	23	4.69E+00	+	1.03E-06
Centrosome cycle (GO:0007098)	95	44	1.54E+01	+	1.48E-06
Biological regulation (GO:0065007)	3256	648	5.27E+02	+	1.60E-06
Regulation of biological process (GO:0050789)	3001	601	4.85E+02	+	3.47E-06
Cellular component organization or biogenesis (GO:0071840)	2286	474	3.70E+02	+	4.60E-06
Mitotic nuclear division (GO:0007067)	195	68	3.15E+01	+	8.27E-06
Regulation of cellular process (GO:0050794)	2786	559	4.51E+02	+	1.25E-05
Centrosome duplication (GO:0051298)	81	38	1.31E+01	+	1.33E-05
Microtubule cytoskeleton organization (GO:0000226)	389	111	6.29E+01	+	1.46E-05
Single-organism process (GO:0044699)	6248	1140	1.01E+03	+	1.62E-05
Negative regulation of gene expression, epigenetic (GO:0045814)	53	29	8.57E+00	+	2.85E-05
Cellular component assembly (GO:0022607)	649	163	1.05E+02	+	3.81E-05
Chromatin silencing (GO:0006342)	52	28	8.41E+00	+	6.75E-05
Negative regulation of organelle organization (GO:0010639)	62	31	1.00E+01	+	7.03E-05
Regulation of biosynthetic process (GO:0009889)	939	217	1.52E+02	+	1.23E-04
Primary metabolic process (GO:0044238)	3604	691	5.83E+02	+	1.45E-04
Regulation of macromolecule metabolic process (GO:0060255)	1293	283	2.09E+02	+	1.54E-04
Regulation of cellular biosynthetic process (GO:0031326)	938	216	1.52E+02	+	1.70E-04
Regulation of macromolecule biosynthetic process (GO:0010556)	888	205	1.44E+02	+	3.01E-04
Regulation of organelle organization (GO:0033043)	221	69	3.57E+01	+	3.69E-04
Regulation of cellular macromolecule biosynthetic process (GO:2000112)	887	204	1.43E+02	+	4.16E-04
DNA repair (GO:0006281)	121	45	1.96E+01	+	4.76E-04
Mitotic cell cycle process (GO:1903047)	457	119	7.39E+01	+	4.86E-04
Meiosis I (GO:0007127)	68	31	1.10E+01	+	4.92E-04
Mitotic cell cycle (GO:0000278)	510	129	8.25E+01	+	7.14E-04
Organic substance metabolic process (GO:0071704)	3872	729	6.26E+02	+	8.57E-04

Term	Backgr. frequency	Sample frequency	Expected	+/-	P-value
Regulation of primary metabolic process (GO:0080090)	1287	276	2.08E+02	+	1.14E-03
Regulation of RNA biosynthetic process (GO:2001141)	802	183	1.30E+02	+	2.68E-03
Regulation of transcription, DNA-templated (GO:0006355)	802	183	1.30E+02	+	2.68E-03
Regulation of nucleic acid-templated transcription (GO:1903506)	802	183	1.30E+02	+	2.68E-03
Regulation of metabolic process (GO:0019222)	1535	318	2.48E+02	+	3.01E-03
Nervous system development (GO:0007399)	1491	310	2.41E+02	+	3.07E-03
Regulation of protein modification process (GO:0031399)	143	48	2.31E+01	+	3.24E-03
Chromosome condensation (GO:0030261)	43	22	6.95E+00	+	3.57E-03
DNA replication (GO:0006260)	79	32	1.28E+01	+	3.72E-03
Cellular component biogenesis (GO:0044085)	718	166	1.16E+02	+	3.87E-03
Negative regulation of cellular component organization (GO:0051129)	112	40	1.81E+01	+	5.06E-03
Reciprocal meiotic recombination (GO:0007131)	28	17	4.53E+00	+	5.07E-03
Reciprocal DNA recombination (GO:0035825)	28	17	4.53E+00	+	5.07E-03
Regulation of cellular metabolic process (GO:0031323)	1395	290	2.26E+02	+	6.74E-03
Cytoskeleton organization (GO:0007010)	597	141	9.65E+01	+	7.48E-03
Regulation of gene expression (GO:0010468)	1134	242	1.83E+02	+	7.73E-03
System development (GO:0048731)	2140	421	3.46E+02	+	9.81E-03
Negative regulation of cellular protein metabolic process (GO:0032269)	129	43	2.09E+01	+	1.19E-02
Negative regulation of protein metabolic process (GO:0051248)	129	43	2.09E+01	+	1.19E-02
Spindle assembly (GO:0051225)	81	31	1.31E+01	+	1.53E-02
Metabolic process (GO:0008152)	4438	809	7.18E+02	+	1.96E-02
Gene silencing (GO:0016458)	99	35	1.60E+01	+	2.30E-02
Regulation of nitrogen compound metabolic process (GO:0051171)	1019	217	1.65E+02	+	2.65E-02
Regulation of nucleobase-containing compound Metabolic process (GO:0019219)	981	210	1.59E+02	+	2.66E-02
Regulation of RNA metabolic process (GO:0051252)	873	190	1.41E+02	+	2.66E-02
Microtubule-based process (GO:0007017)	495	118	8.00E+01	+	2.77E-02
Regulation of cell cycle (GO:0051726)	325	84	5.26E+01	+	2.85E-02
Neurogenesis (GO:0022008)	1340	275	2.17E+02	+	2.93E-02
Multicellular organismal development (GO:0007275)	2687	511	4.35E+02	+	3.00E-02
Regulation of mitotic cell cycle (GO:0007346)	212	60	3.43E+01	+	3.46E-02
DNA-dependent DNA replication (GO:0006261)	43	20	6.95E+00	+	3.59E-02
Generation of precursor metabolites and energy (GO:0006091)	149	7	2.41E+01	-	3.81E-02
Cell differentiation (GO:0030154)	2124	412	3.44E+02	+	4.34E-02
Regulation of cellular component organization (GO:0051128)	547	127	8.85E+01	+	4.36E-02
Regulation of cell cycle process (GO:0010564)	191	55	3.09E+01	+	4.59E-02

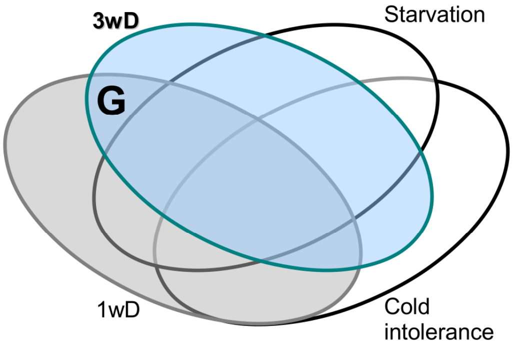

304 genes

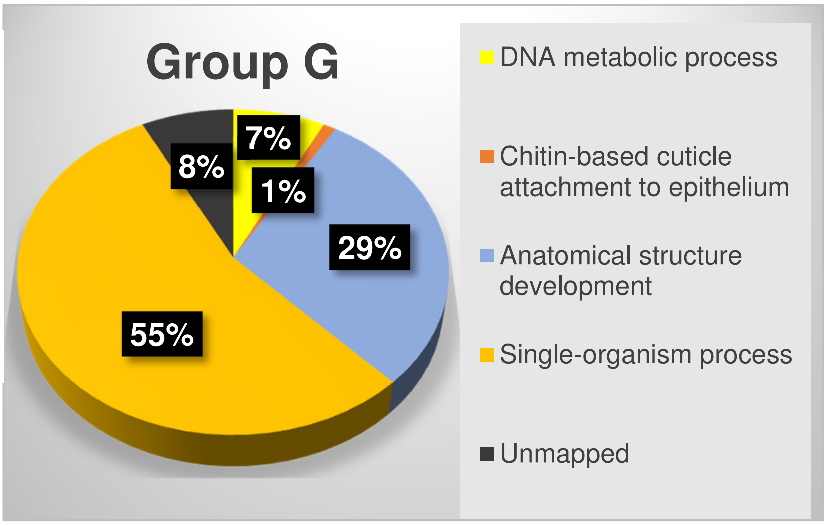

Term	Backgr. frequency	Sample frequency	Expected	+/-	P-value
DNA replication (GO:0006260)	79	14	1.64E+00	+	2.88E-07
DNA metabolic process (GO:0006259)	333	22	6.89E+00	+	3.61E-04
DNA replication initiation (GO:0006270)	14	5	2.90E-01	+	1.97E-03
Chitin-based cuticle attachment to epithelium (GO:0040005)	3	3	6.21E-02	+	5.73E-03
DNA-dependent DNA replication (GO:0006261)	43	7	8.90E-01	+	5.81E-03
Cell cycle DNA replication (GO:0044786)	24	5	4.97E-01	+	2.47E-02
Anatomical structure development (GO:0048856)	2856	85	5.91E+01	+	2.77E-02
Single-organism process (GO:0044699)	6248	159	1.29E+02	+	3.82E-02

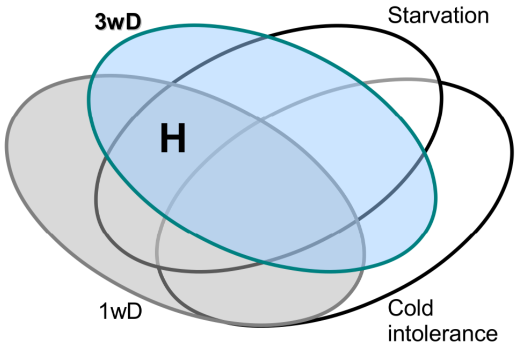

83 genes

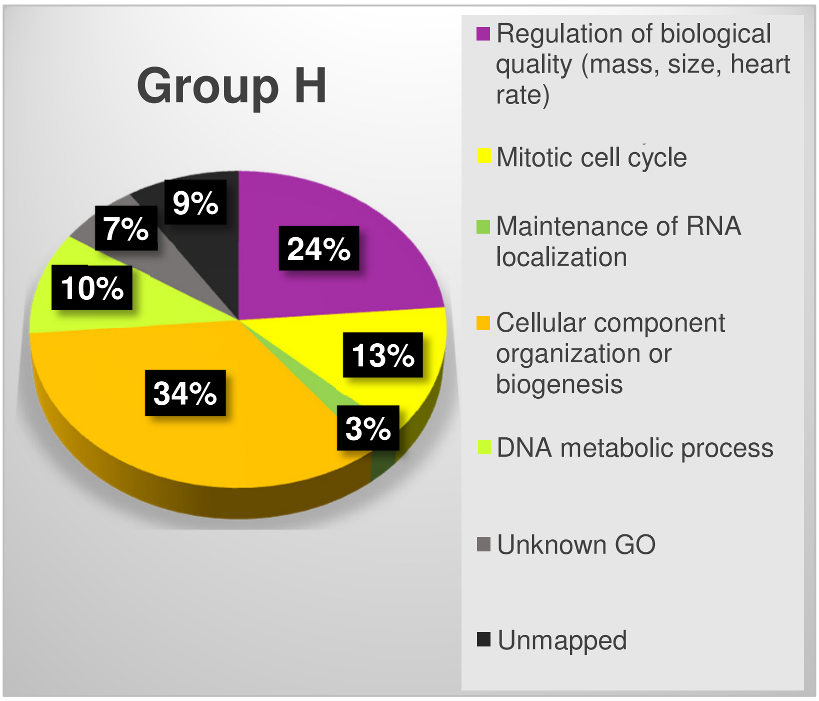

Term	Backgr. frequency	Sample frequency	Expected	+/-	P-value
Regulation of biological quality (GO:0065008)	906	18	5.05E+00	+	7.24E-05
Regulation of mitosis (GO:0007088)	66	5	3.68E-01	+	1.41E-03
Regulation of nuclear division (GO:0051783)	70	5	3.91E-01	+	1.86E-03
Regulation of cell division (GO:0051302)	84	5	4.69E-01	+	4.35E-03
Maintenance of pole plasm mRNA location (GO:0046594)	3	2	1.67E-02	+	5.20E-03
Cellular component organization or biogenesis (GO:0071840)	2286	26	1.28E+01	+	6.57E-03
Regulation of mitotic cell cycle (GO:0007346)	212	7	1.18E+00	+	7.18E-03
Mitotic cell cycle process (GO:1903047)	457	10	2.55E+00	+	8.64E-03
maintenance of RNA location (GO:0051237)	4	2	2.23E-02	+	9.20E-03
Pole cell development (GO:0007277)	27	3	1.51E-01	+	1.87E-02
Mitotic cell cycle (GO:0000278)	510	10	2.85E+00	+	2.04E-02
DNA metabolic process (GO:0006259)	333	8	1.86E+00	+	2.08E-02
Cell cycle process (GO:0022402)	617	11	3.44E+00	+	2.34E-02
Regulation of cell cycle process (GO:0010564)	191	6	1.07E+00	+	2.73E-02
Cellular component organization (GO:0016043)	2229	24	1.24E+01	+	2.95E-02
Mitotic nuclear division (GO:0007067)	195	6	1.09E+00	+	3.04E-02
Regulation of cellular component organization (GO:0051128)	547	10	3.05E+00	+	3.49E-02
