# Supplementary material for: Slowed aging during reproductive dormancy is reflected in genome-wide transcriptome changes in Drosophila melanogaster
Source: BMC Genomics. 2016 Jan 13;17:50. doi: 10.1186/s12864-016-2383-1 (PMC4711038; doi:10.1186/s12864-016-2383-1)
Supplement: Additional file 15: Table S5. — Primers used for quantitative real-time PCR. (PDF 55 kb) [file 12864_2016_2383_MOESM15_ESM.pdf]

Additional file 15: Table S5. Primers used for quantitative real-time PCR

Target	Forward primer	Reverse primer
CG16756	TCGAATTGGATTTGTTTGCTAGA	CCGACTCCCTTAAATTTTCGT
Fatty Acid Desaturase 2	TACCACCATGTCTTTCCCTG	GCTTGACCAAATCCAGTGAG
Heat shock gene 67Bc	GCAGCTGAGATTGTGGATTC	TCTTTGTGTATCTGGCGGAA
Larval Serum Protin 1 gamma	GATTACACCTACTCCTCGGG	TAGGCACAAAGAAGTCGGAT
Necrotic	TCCTATGCCAAGTTCGTACC	CCACGTGACCTATGAACAGA
Neuropeptide receptor F	AGTTAAGACACACGCGACTA	TGGGGGCTCATTCAATTACT
Odorant Binding Protein 19b	GTATAACGAGGACAAGACGGT	TCCTTGATCTCGGGAATCTTG
Period	TGCAAGCCGAGGATAATGTA	CTTAGGGCTGAGAAGGGTG
Actin 88F	AGGGTGTGATGGTGGGTATG	CTTCTCCATGTCGTCCCAGT
Timeless, all isoforms	ACATAACACAGTTTGCCACG	TCATGGTGAAGATGCAGTCA
Timeless, all isoforms except N & O	TCCATGAAGTCCTCGTTCG	CATCGCTCACATACATTCTGG
Timeless, isoforms N & O	TGACGGAAATGACCAAGGAA	TGGTCCACAAATGTTAAAAATGTTA
Vitelline membrane 32E	CGCCTACACAGAACAGGT	TGTTGACCCTGGATTCCTTG
